# Activation of N_2_O, CO_2_, and CO at a sterically protected phosphorus center

**DOI:** 10.1039/d6dt00986g

**Published:** 2026-04-29

**Authors:** John S. Wenger, William J. Rowe, Meera Mehta

**Affiliations:** a Department of Chemistry, University of Oxford 12 Mansfield Road Oxford OX1 3QR UK john.wenger@chem.ox.ac.uk meera.mehta@chem.ox.ac.uk

## Abstract

Functionalization of a sterically encumbered phosphorus precursor enables varied activation pathways for N_2_O, CO_2_, and CO. The potasssium phosphanide salt, [K(crypt)][(M^s^FluInd*)PH] (crypt = 2.2.2.cryptand; M^s^FluInd* = a sterically demanding hydrindacenyl substituent), was synthesized and treated with either N_2_O or ^13^CO_2_ to afford the potassium phosphinate, [K(crypt)][(M^s^FluInd*)PHO_2_], or the potassium phosphacarboxylate, [K(crypt)][(M^s^FluInd*)PH(^13^CO_2_)], respectively. Deprotonation of the TMS-functionalized (TMS = trimethylsilyl) phosphine, (M^s^FluInd*)PTMSH, followed by treatment with either N_2_O or ^13^CO_2_ resulted in the formation of a phoshanorcaradiene, (M^s^FluInd*)P, and an arylphosphaketene, (M^s^FluInd*)P^13^CO, respectively. Reversible CO binding at phosphorus allows for the interconversion between (M^s^FluInd*)P and (M^s^FluInd*)PCO. The mechanism for the formation of (M^s^FluInd*)PCO from (M^s^FluInd*)P and CO was investigated computationally.

## Abstract 2

Low-valent phosphorus precursors activate N_2_O, CO_2_, and CO to access rare chemical species. Our investigation of these reactions ultimately provides insights into the degradation of industrial by-products at Earth-abundant, main-group elements.

## Graphical abstract

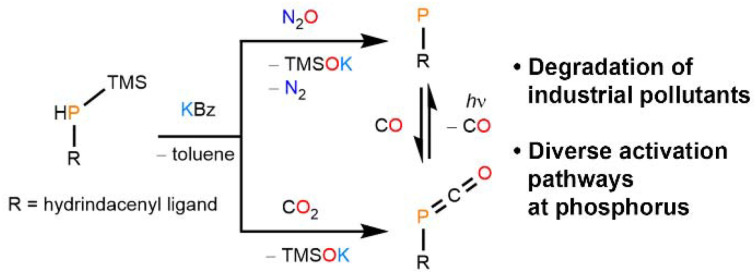

## Introduction

The reactivity of low-valent phosphorus species is currently under intense investigation to access new avenues in small-molecule activation.^1^ Reactivity patterns between functionalized phosphorus reagents and N_2_O are now well-established.^2^ Typically, N_2_O reacts as an O-atom transfer reagent to form highly stable P–O bonds with the generation of either free N_2_ or N_2_-capture products.^3^ Alternatively, the N_2_O molecule may remain intact in the formation of P–N bonded adducts.^2,4^

**Fig. 1:**
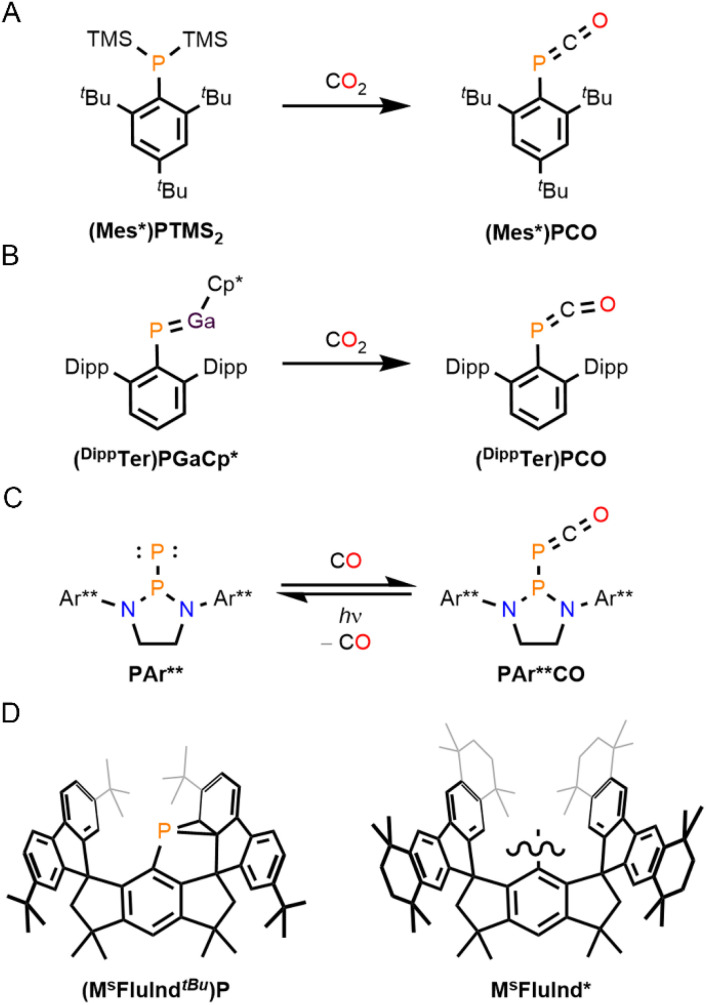
Synthesis of (A) (Mes*)PCO, (B) (^Dipp^Ter)PCO, and (C) P^Ar^**CO. (D) Depictions of (M^s^FluInd^*t*Bu^)P and the hydrindacenyl ligand M^s^FluInd*.

Phosphaketenes represent a versatile class of molecular synthons, and previously reported arylphosphaketenes, (Mes*)PCO (Mes* = 2,4,6-tri-*tert*-butylphenyl) and (^Dipp^Ter)PCO (^Dipp^Ter = 2,6-bis(2,6-diisopropylphenyl)-phenyl) were synthesized *via* O-atom abstraction from CO_2_ by functionalized phosphorus precursors, (Mes*)PTMS_2_ and (^Dipp^Ter)PGaCp* (Cp* = pentamethylcyclopentadiene), respectively (Fig. 1A and B).^5^ The formation of phosphaketenes by direct CO activation has also been observed.^6^ The sterically encumbered monomeric (phosphino)phosphinidene, P^Ar^** (Ar** = 2,6-bis[di(4-*tert*-butylphenyl)methyl]-4-methylphenyl), binds CO at the terminal, monovalent P atom to form the (phosphino)phosphaketene, P^Ar^**CO, which engages in metallomimetic ligand-exchange reactions and loses CO *via* photolysis to form the precursor, P^Ar^** (Fig. 1C).^1*a*–*c*^

Sterically demanding hydrindacenyl substituents, such as M^s^FluInd*, have gained popularity to access highly reactive, unsaturated main group compounds (Fig. 1D).^7^ Notably, the hydrindacenyl phosphanorcaradiene, (M^s^FluInd^*t*Bu^)P, was shown to engage in molecular-strain induced, phosphinidene reactivity in the activation of small molecules, including isocyanides, which represent isoelectronic analogues of CO (Fig. 1D).^1*d*^

We recently reported the sterically encumbered hydrindacenyl phosphines, (M^s^FluInd*)PCl_2_ (1), (M^s^FluInd*)PH_2_ (2) and (M^s^FluInd*)PTMSH (3), which were employed as precursors in the first syntheses of thermally robust arylhalodiphosphenes, (M^s^FluInd*)PPX (X = Cl, Br, I).^8^ We rationalized that compounds containing an anionic phosphorus site within the sterically protected environment created by the M^s^FluInd* ligand could activate gaseous small molecules.

Herein, we report the isolation of the primary phosphanide, [K(crypt)][(M^s^FluInd*)PH] (4). In the presence of excess N_2_O or ^13^CO_2_, compound 4 forms either the oxidation product, [K(crypt)][(M^s^FluInd*)PHO_2_] (5), or the ^13^CO_2_-captured product, [K(crypt)][(M^s^FluInd*)PH(^13^CO_2_)] (^13^6), respectively. In contrast, when compound 3 is reacted with potassium benzylate before being treated with N_2_O or ^13^CO_2_, the phosphanorcaradiene, (M^s^FluInd*)P (7), or the arylphosphaketene, (M^s^FluInd*)P^13^CO (^13^8), are obtained, respectively. Compound 7 is quantitatively converted to (M^s^FluInd*)PCO (8) in the presence of CO gas under mild conditions, and photolysis of compound 8 results in the formation of compound 7*via* the elimination of CO.

## Results and discussion

**Fig. 2:**
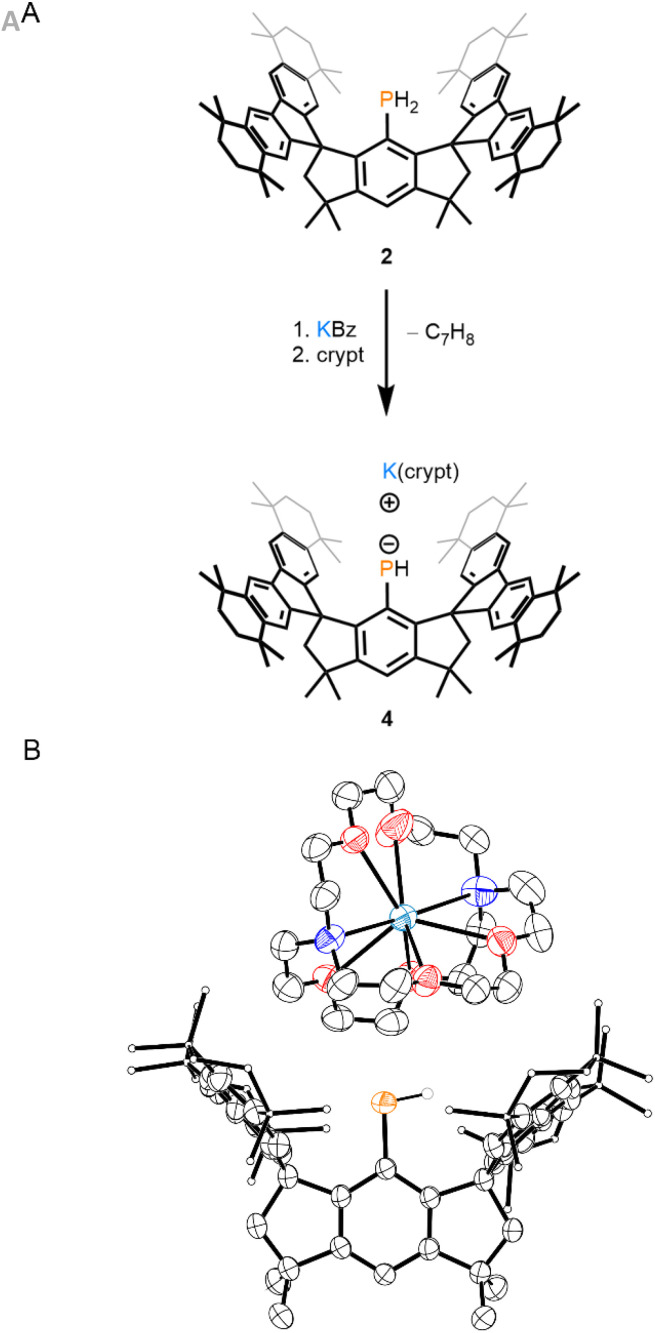
(A) Synthesis of 4. (B) Thermal ellipsoid plot (50% probability) of 4. C-bound H atoms and disordered components are omitted for clarity. Select C atoms and H atoms are shown as spheres of arbitrary radius for clarity. Color code: P orange, O red, C black, H grey, K teal, N blue.

Compound 4 was synthesized by treatment of compound 2 with potassium benzylate followed by crypt in benzene (Fig. 2A). Crypt was included in the reaction mixture to efficiently sequester the K cation from the primary phosphanide and to facilitate the isolation of 4 as a crystalline salt. Fully sequestered, or “naked”, primary phosphanide anions remain rare and often require multiple equivalents of crown ether to synthesize.^9^ Recrystallization from a mixture of tetrahydrofuran (THF)/pentane afforded dark-green crystals of 4·(THF)(pentane)_0.5_ in a 77% yield. ^31^P NMR analysis of the product reveals a doublet resonating at −69.9 ppm with a ^1^*J*_PH_ value of 154 Hz. Single-crystal X-ray diffraction (SC-XRD) analysis of 4 confirms the sequestration of the K cation and no P–K interaction (Fig. 2B). The P–C bond length in 4 is 1.788(2) Å and is significantly shorter than that of 2 [1.8424(15) Å], consistent with delocalization of a P-centered lone pair into the hydrindacenyl substituent.^8^ Furthermore, the M^s^FluInd* ligand in 4 appears to adopt a more open conformation relative to 2 to accommodate the {PH} and {K(crypt)} units. The centroid–centroid distance between the five-membered rings of the fluorenyl substituents in 4 [6.8513(17) Å] is greater than that of 2 [6.5460(7) Å].^8^

**Fig. 3:**
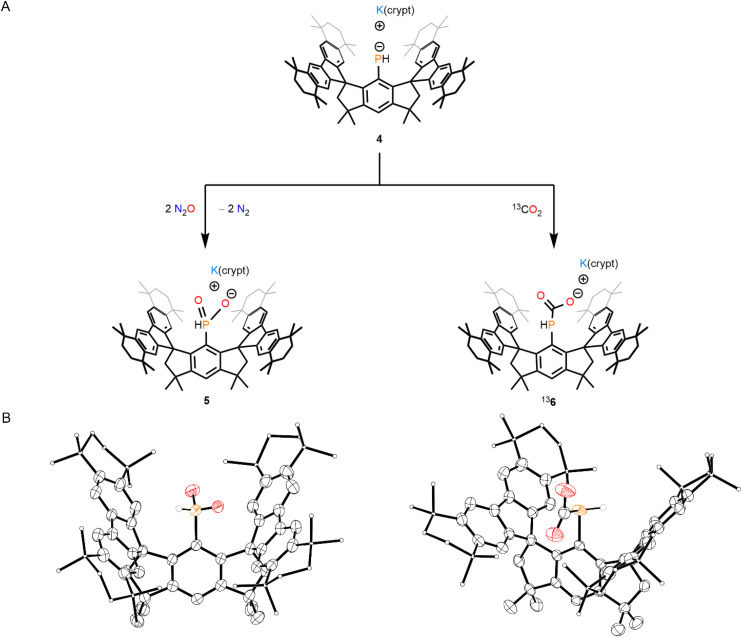
(A) Synthesis of 5 and ^13^6. (B) Thermal ellipsoid plots (50% probability) of 5 (left) and ^13^6 (right). C-bound H atoms, disordered components, and counter cations are omitted for clarity. Select C atoms and H atoms are shown as spheres of arbitrary radius for clarity. Color code: P orange, O red, C black, H grey.

Treatment of 4 with an excess of N_2_O results in the formation of 5 in an 86% yield (Fig. 3A). The ^31^P NMR spectrum of 5 features a single doublet resonating at 1.2 ppm with a large ^1^*J*_PH_ coupling constant of 476 Hz, consistent with the presence of a phosphinate anion.^10^ SC-XRD analysis of the product confirms the presence of two P-bound O atoms, indicating that the P(i) center of 4 had been oxidized by two equivalents of N_2_O to form a pentavalent phosphorus species (Fig. 3B). The {PHO_2_} motif in 5 does not coordinate the K cation and is disordered about two positions, which precludes much meaningful discussion about crystallographic metrics. Notably, the centroid–centroid distance between the five-membered rings of the fluorenyl substituents in 5 [6.9134(16) Å] is greater than that of 4, consistent with the presence of the larger {PHO_2_} motif.

When 4 is treated with 1 atm of ^13^CO_2_ at room temperature, the ^13^CO_2_ capture-product ^13^6 is generated *in situ* (Fig. 3A). Despite attempts to perform the reaction under rigorously dry conditions, compound 2 is also observed in the reaction mixture, which we suggest arises by the protonation of 4 by adventitious water. The ^31^P{^1^H} NMR spectrum of ^13^6 features a singlet at −85.5 ppm. The ^13^C{^1^H} NMR spectrum similarly exhibits a prominent singlet associated with the isotopically labelled ^13^C atom at 168.0 ppm. We suggest that dynamic interaction between the ^13^C-bound O atoms and the sequestered K cation in solution results in the broadening of the ^31^P and ^13^C NMR resonances, such that the ^1^*J*_PC_ coupling is not observed. SC-XRD analysis of ^13^6 confirms the formation of the P–^13^CO_2_ bond, with a bond length of 1.906(2) Å, and coordination of the {PH^13^CO_2_} motif to the K cation in the solid state, with an O–K bond length of 2.741(2) Å (Fig. 3B). The P–C_aryl_ bond length is 1.827(2) Å, and the C–P–^13^C bond angle is 104.41(10)°. The formation of the O–K bond seemingly necessitates a relatively open conformation of the M^s^FluInd* unit to accommodate the {K(crypt)} cation, and the centroid–centroid distance between the five-membered rings of the fluorenyl substituents in ^13^6 is 6.9296(13) Å. Further, a high-resolution electrospray ionization mass spectrometry experiment clearly identified the isotopically labelled anion, ^13^6–K^−^ (^13^6–K^−^ refers to the anion formed upon loss of the K cation from ^13^6). Compound ^13^6 was found to be unstable and could not be separated from the decomposition product, 2.

**Fig. 4:**
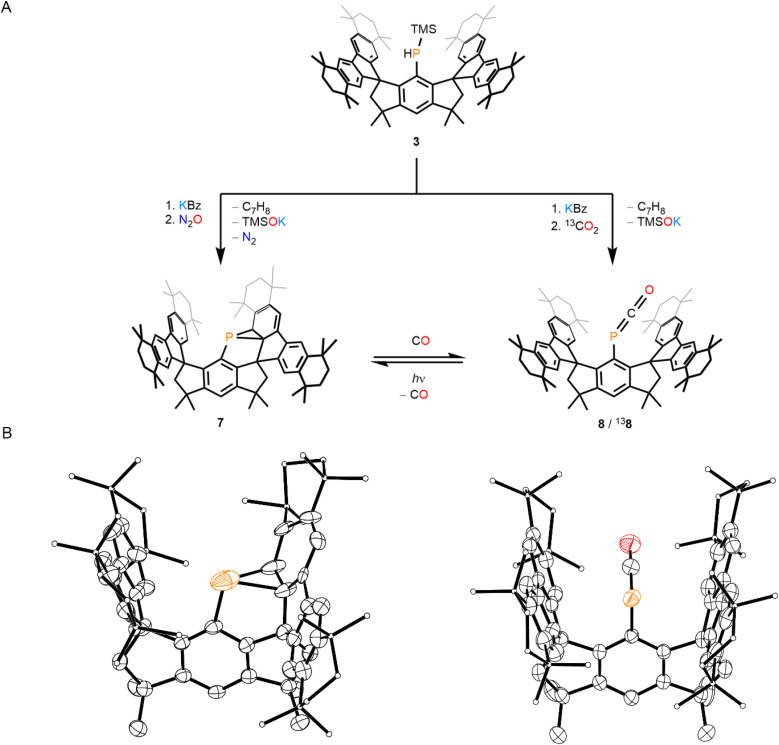
(A) Synthesis of 7, 8, and ^13^8. (B) Thermal ellipsoid plots (50% probability) of 7 (left) and ^13^8 (right). C-bound H atoms, disordered components, and counter cations are omitted for clarity. Select C atoms are shown as spheres of arbitrary radius for clarity. Color code: P orange, O red, C black.

**Fig. 5:**
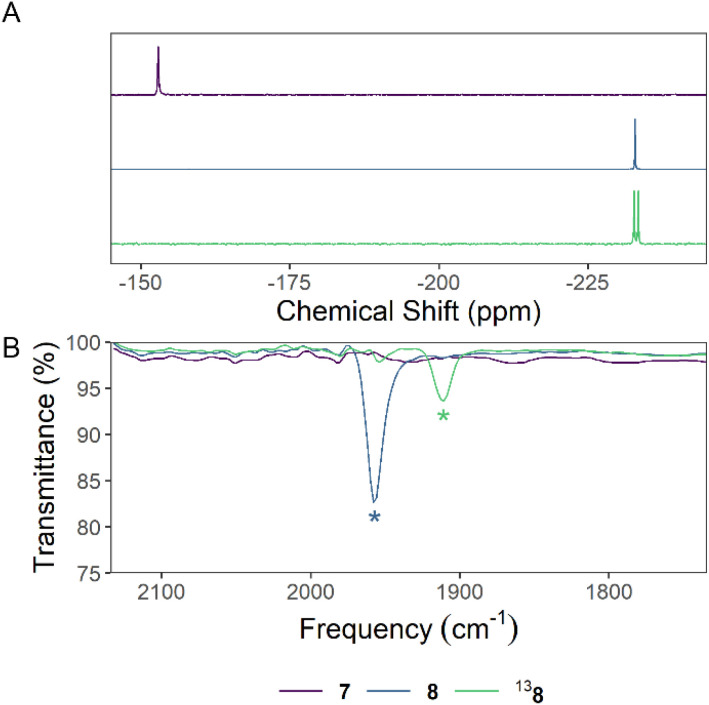
Stacked (A) ^31^P{^1^H} NMR spectra and (B) IR spectra of 7, 8, and ^13^8. Signals in the IR spectra assigned to a carbonyl C–O bond stretching mode are denoted with an asterisk.

Next, we investigated the TMS-functionalized phosphine, 3, as a precursor for analogous reactions involving N_2_O and CO_2_. Treatment of 3 with KBz followed by N_2_O results in the formation of 7 in a 59% yield (Fig. 4A). ^1^H and ^13^C{^1^H} NMR spectra of 7 appear complex, consistent with the desymmetrization of the M^s^FluInd* backbone. The ^31^P{^1^H} NMR spectrum of 7, however, features a single resonance at −153.0 ppm (Fig. 5A). SC-XRD analysis of 7·(toluene)_0.5_ confirms its identity as a hydrindacenyl phosphanorcaradiene, with similar structural characteristics to the literature-known species, (M^s^FluInd^*t*Bu^)P (Fig. 4B).^1*d*^ However, the central P atom is disordered across two positions, precluding much meaningful discussion of bond metrics. In this preparation of 7, N_2_O is activated by the elimination of KOTMS and N_2_ along with dearomatization of a fluorenyl substituent by the resulting low-coordinate P atom. Compound 7 was also prepared independently by treatment of 1 with two equivalents of KC_8_, in an 81% yield, following an adapted literature protocol.^1*d*^

We exposed compound 7 to 1 atm of CO at 50 °C overnight to form the arylphosphaketene, 8, *in situ* (Fig. 4A). ^1^H and ^13^C{^1^H} NMR spectra of 8 reveal a symmetrical M^s^FluInd* environment. The ^31^P NMR spectrum of 8 features a characteristic resonance at −233.1 ppm (Fig. 5A), and the ^13^C{^1^H} NMR spectrum of 8 features a doublet at 203.0 ppm with a ^1^*J*_PC_ coupling constant of 113 Hz. The IR spectrum of 8 features a strong band at 1948 cm^−1^ associated with the carbonyl stretch of the {PCO} unit (Fig. 5B). Crystals of ^13^8 (*vide infra*) were grown from a concentrated mixture of hexane/toluene and feature crystallographic disorder of the {PCO} motif about two positions (Fig. 4B). In ^13^8, the M^s^FluInd* motif adopts a more closed conformation relative to 4, 5, or ^13^6, and features a lower centroid–centroid distance between the five-membered rings of the fluorenyl substituents of 6.1909(14) Å.

Exposure of 8 to 390 nm light for 2 h results in nearly quantitative conversion back to 7 (Fig. 4A and SI Fig. S42).^5*c*^ Despite working under dark conditions, we were unable to isolate 8 as a pure bulk material due to rapid decomposition to form 7 during workup procedures.

We exposed a reaction mixture containing KBz and 3 to 1 atm of ^13^CO_2_ (Fig. 4A). The resulting solution contains a major product with spectral data that match those of 8, but with the expected variations arising from ^13^C enrichment at the phosphaketene motif, and we assign these signals to ^13^8. The ^31^P nucleus of ^13^8 resonates as a doublet in the ^31^P NMR spectrum and the coupled resonance in the ^13^C NMR spectrum appears with dramatically enhanced intensity (Fig. 5A). The reaction mixture containing ^13^8 was stripped of solvent and the IR spectrum of the resulting solid residue features a carbonyl stretch at lower wavenumber than 8 at 1911 cm^−1^ (Fig. 5B), as expected.

**Fig. 6:**
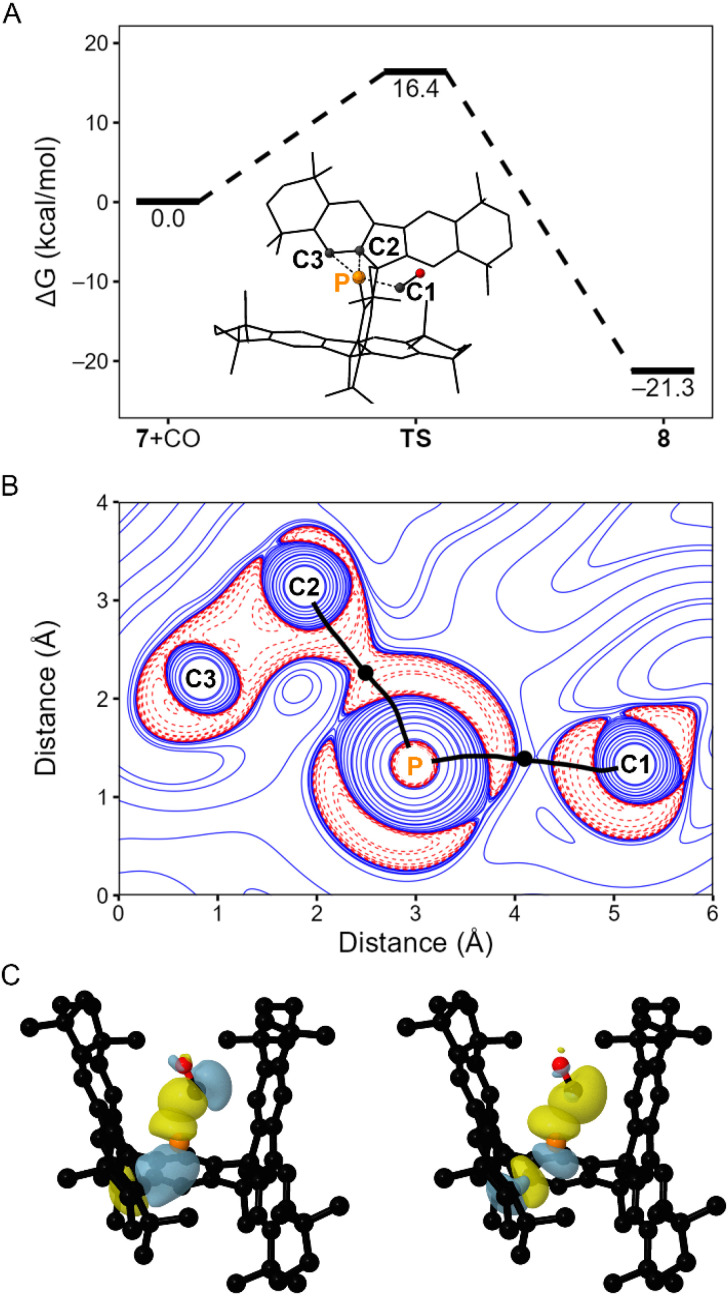
(A) Gibbs free energy profile (r^2^SCAN-3c) for the synthesis of 8 from 7 and CO *via* transition state, TS. A ball-and-stick diagram of TS is shown in the inset, with atoms P, C1, C2, and C3 labelled. (B) Two-dimensional plot of ∇^2^*ρ* (X2C-PBE0-D3BJ/x2c-TZVPPall//r^2^SCAN-3c) of TS in the P–C1–C2 plane; bond paths are shown as black lines, (3, −1) critical points are shown as black circles, positive contour lines are shown as blue solid lines, and negative contour lines are shown as red dashed lines. (C) Surface plots (PBE0-D3BJ/def2-TZVP//r^2^SCAN-3c) (isovalue = 0.06) depicting overlap between the C1-centered vacant p-orbital and a filled P–C3 NBO (left) and overlap between a filled C1-centered lone pair and the P–C3 σ* NBO (right). Further details are provided in the SI.

In a prior report, transition-state calculations for the activation of substrates including an alkene, alkyne, and silane by (M^s^FluInd^*t*Bu^)P revealed a reaction mechanism in which concerted breaking of the PC_2_ ring with the activation of the C=C, C≡C, or Si–H bonds, respectively.^1*d*^ In contrast, activation of an amine by (M^s^FluInd^*t*Bu^)P proceeded through the breaking of the PC_2_ ring upon coordination of the amine to the P center, followed by activation of the N–H bond.^1*d*^ To build on these previous insights, we investigated the reaction mechanism for the formation of 8 from 7 and CO computationally. A simultaneous, two-dimensional relaxed surface scan (r^2^SCAN-3c) was performed, in which the P–C1 (C1 refers to the carbonyl carbon) bond was extended and the P–C3 (C3 refers to a C atom in a fluorenyl group) bond was contracted, starting from the theoretical coordinates for 8 (Fig. 6A and SI Fig. S44, 45). The optimized coordinates obtained near the maximum of the scanned potential energy surface were used as an input for a transition-state search, which identified the transition state, TS (Fig. 6A). The Δ*G* of formation of TS is 16.4 kcal mol^−1^ higher than that of the starting materials and 37.7 kcal mol^−1^ higher than that of 8.

Topological analysis (X2C-PBE0-D3BJ/x2c-TZVPPall//r^2^SCAN-3c) of the electron density (*ρ*) of TS identified (3, −1) critical points along the P–C1 and P–C2 interatomic vectors (Fig. 6B), and no bond critical point along the P–C3 interatomic vector.^11^ Inspection of the Laplacian of *ρ* (∇^2^*ρ*) of TS in the plane defined by the P, C1, and C3 atoms finds negative values along the P–C2 interatomic vector, consistent with the presence of a covalent bonding interaction (Fig. 6B). The P–C1 bonding region does not feature similar continuity of negative values and is consistent with dative interactions between the P and C1 atoms.

Natural bond orbital (NBO) analysis (PBE0-D3BJ/def2-TZVP//r^2^SCAN-3c) of TS identified a C1-centered lone pair and P–C2 and P–C3 bonding and antibonding orbitals (SI Fig. S46 and Table S7). Second order perturbation theory (E^2^) analysis reveals delocalization of electron density from the P–C3 bonding orbital into a vacant p-orbital at C1 to afford an energy of stabilization of 57.9 kcal mol^−1^ (Fig. 6C). Additionally, the lone pair at C1 delocalizes into the P–C3 σ* orbital to afford an energy of stabilization of 50.6 kcal mol^−1^ (Fig. 6C).

These computational data collectively suggest that the formation of 8 from 7 and CO proceeds through a concerted mechanism in which coordination of the CO unit to the P center results in the breaking of the PC_2_ ring, in agreement with previous results.^1*d*^ Topological and NBO analyses of TS suggest that the P–C3 bond is cleaved prior to the breaking of the P–C2, whilst the P–C1 bond can still be considered dative in nature.

## Conclusions

In summary, we have investigated sterically encumbered phosphanides in the activation of N_2_O, CO_2_, and CO. Efforts to expand the reactivity patterns reported herein to catalytic processes involving these small molecules are currently underway.

## Author contributions

J. S. W.: conceptualization, data curation, funding acquisition, investigation, methodology, visualization, writing – original draft, writing – review and editing. W. J. R.: investigation, writing – review and editing. M. M.: conceptualization, funding acquisition, project administration, resources, supervision, writing – review and editing.

## Conflicts of interest

There are no conflicts to declare.

## Supplementary Material

DT-055-D6DT00986G-s001

DT-055-D6DT00986G-s002

## Data Availability

The data supporting this article have been included as part of the supplementary information (SI). Supplementary information is available. See DOI: https://doi.org/10.1039/d6dt00986g. CCDC 2537503 (7·(toluene)_0.5_), 2537504 (4), 2537505 (^13^8), 2537506 (^13^6) and 2537507 (5) contain the supplementary crystallographic data for this paper.^12*a*–*e*^
