# Supplementary material for: Activation of N2O, CO2, and CO at a sterically protected phosphorus center
Source: Dalton Trans. 2026 Apr 29;55(20):7884–90. doi: 10.1039/d6dt00986g (PMC13140154; doi:10.1039/d6dt00986g)
Supplement: DT-055-D6DT00986G-s001 [file DT-055-D6DT00986G-s001.pdf]

Supplementary Information (SI) for Dalton Transactions.
This journal is © The Royal Society of Chemistry 2026

Supporting Information for

Activation of N_2O, CO_2, and CO at a sterically protected phosphorus center

John S. Wenger,*[a] *William J. Rowe,* [a] *and Meera Mehta**[a]

[a] Department of Chemistry, University of Oxford, 12 Mansfield Road, Oxford, OX1 3QR, U.K. john.wenger@chem.ox.ac.uk, meera.mehta@chem.ox.ac.uk

Contents

1. Experimental Methods ... 3

2. Synthesis and characterization of novel compounds ... 9

2.1 Synthesis of [K(crypt)][(M^{s}FluInd*)PH]•(THF)(pentane)$_{0.5}$ (4•(THF)(pentane)$_{0.5}$). ... 9

2.2 Synthesis of [K(crypt)][(M^{s}FluInd*)PO$_2$H] (5). ... 15

2.3 Synthesis of [K(crypt)][(M^{s}FluInd*)P(CO$_2$)H] (^{13}6) *in situ*. ... 21

2.4 Synthesis of (M^{s}FluInd*)P (7). ... 28

2.5 Synthesis of (M^{s}FluInd*)PCO (8 and ^{13}8) *in situ*. ... 35

2.6 Photolysis of 8. ... 42

3. Crystallographic Tables ... 44

4. Computational Data ... 47

5. References. ... 74

1. Experimental Methods ... 3

2. Synthesis and characterization of novel compounds. ... 9

2.1 Synthesis of [K(2.2.2.crypt)][(M^{s}FluInd*)PH]•(THF)(pentane)$_{0.5}$ (4•(THF)(pentane)$_{0.5}$). ... 9

2.2 Synthesis of [K(2.2.2.crypt)][(M^{s}FluInd*)PO$_2$H] (5). ... 15

2.3 Synthesis of [K(2.2.2.crypt)][(M^{s}FluInd*)P(CO$_2$)H] (^{13}6) *in situ*. ... 21

2.4 Synthesis of (M^{s}FluInd*)P (7). ... 28

2.5 Synthesis of (M^{s}FluInd*)PCO (8 and ^{13}8) *in situ*. ... 35

2.6 Photolysis of 8. ... 42

3. Crystallographic Tables ... 44

4. Computational Data ... 47

5. References. ... 74

1. Experimental Methods

General Methods. Dimethyl isophthalate, *tert*-butyl lithium (1.7 M in pentane), PCl_3, $AlCl_3$, BCl_3 (1M in hexane mixed isomers), $LiAlH_4$, and $Et_4NOH•(H_2O)_5$ were purchased from Sigma-Aldrich. Fluorene, sulfuric acid, hydrochloric acid (aq.), methylmagnesium bromide (3 M in diethyl ether), and 2.2.2.cryptand (crypt) were purchased from Thermo Fisher Scientific. 2,5-Dimethyl-2,5-hexanediol was purchased from Fluorochem. Trimethylsilyl chloride (TMSCl) was purchased from ChemCruz. N-Bromosuccinimide was purchased from Alfa Aesar. N_2O and CO were purchased from CK Isotopes Limited. $^{13}CO_2$ was purchased from Cambridge Isotope Laboratories, Inc. Reagents purchased from commercial vendors were used as received, unless otherwise stated. (M^sFluInd)*Br was synthesized as previously reported;[1] however, we provide the overall synthetic route below with literature references for the synthesis of each precursor employed in this work. Potassium benzylate (KBz) was synthesized as previously reported.[2] (M^sFluInd*)PCl_2 (**1**), (M^sFluInd*)PH_2 (**2**), and (M^sFluInd*)PTMSH (**3**) were synthesized as previously reported.[3] All manipulations were performed under an inert atmosphere using standard Schlenk line, and glovebox (MBraun Unilab). Glassware was flame dried prior to use. Glass filter papers were oven-dried prior to use. Solvents diethyl ether (Et_2O), benzene, toluene, hexane, dichloromethane (DCM), pentane, and tetrahydrofuran (THF) were purified using an Innovative Technologies anhydrous engineering solvent purification system and degassed prior to being stored on 3 Å molecular sieves. C_6D_6 was degassed and stored on 3 Å molecular sieves. Gaseous N_2O, $^{13}CO_2$, and CO were transferred from purchased cylinders into ampoules containing 3 Å molecular sieves to dry for at least 6 h prior to use.

NMR Spectroscopy. ^1H, $^{13}C\{^1H\}$, ^{31}P, and $^{31}P\{^1H\}$ were recorded on a Bruker AVIII 400 (operating frequencies: 400.20 MHz, 100.64 MHz, and 162.00 MHz for ^1H, ^{13}C, and ^{31}P respectively) or Bruker AVIII 500 (operating frequencies: 499.94, 125.71, and 202.37 MHz for ^1H, ^{13}C, and ^{31}P respectively) spectrometer. ^1H and $^{13}C\{^1H\}$ NMR spectra were referenced internally to residual solvent signals ^1H δ = 7.16 ppm, $^{13}C\{^1H\}$ δ = 128.02 ppm for C_6D_6. ^{31}P and $^{31}P\{^1H\}$ spectra were referenced externally to H_3PO_4. Solution phase NMR samples were prepared under an inert atmosphere in 5 mm J Young NMR tubes. NMR data were analyzed using MestReNova software.

X-ray Crystallography – Instrument. X-ray diffraction data for **4**, **5**, ^{13}**6**, and ^{13}**8** were collected with an Oxford Diffraction Supernova dual-source diffractometer equipped with a 135 mm Atlas CCD area detector. X-ray diffraction data for **7**•(toluene)$_{0.5}$ were collected on a dual wavelength Rigaku FR-X rotating anode diffractometer equipped with an AFC-11 4-circle kappa geometry goniometer, a Hypix-6000HE detector, VariMAXTM microfocus optics, and an Oxford Cryosystems Cryostream 800 nitrogen flow gas system. Data were collected and reduced using Rigaku CrysAlisPro (version 43).[4]

X-ray Crystallography – Structure Solution and Refinement. The structures were solved using SHELXT and refined using SHELXL within the suite of programs provided by Olex2,[5] following established strategies.[6] All non-H atoms were refined anisotropically. C-bound H atoms were placed at calculated positions and refined with a riding model and coupled isotropic displacement parameters (1.2 × Ueq for non-methyl C-H atoms and 1.5 × Ueq for methyl groups). In the case of **4** and ^{13}**6**, the P-bound H atom was located in the Fourier difference map and treated with a distance (DFIX) restraint. In the case of **5**, the {PHO_2} unit is disordered, and the location of the P-bound H atom could not be reliably located in the Fourier difference map and was placed at a calculated, chemically reasonable position with a distance (DFIX) restraint. Crystallographic data for **4**, **5**, ^{13}**6**, **7**•(toluene)$_{0.5}$, and ^{13}**8** have been deposited *via* the joint CCDC/FIZ Karlsruhe deposition service under 2537504, 2537507, 2537506, 2537503, and 2537505, respectively.

X-ray Crystallography – Treatment of Crystallographic Disorder. Disordered components were modelled with similarity (SIMU), rigid bond (RIGU), and distance (SADI) restraints where appropriate. Non-default restraint values were employed when chemically reasonable and necessary for stable refinement. In the case of **5**, **7**•(toluene)$_{0.5}$, and **8**, the {PHO_2}, {PC_2}, and {PCO} units, respectively, are disordered about two positions in each case, and this disorder precludes meaningful discussion of bond metrics.

X-ray Crystallography – Use of Solvent Masks. The crystal structures of ^{13}**6**, **7**•(toluene)$_{0.5}$, and ^{13}**8** contained severely disordered solvent, which could not be reliably modelled, and, as such, these structures were refined with solvent masks in *Olex2*. The solvent mask applied in the case of ^{13}**6** identified electron-containing voids in the unit cell

with a total electron count of 82.2 electrons in a volume of 598.6 Å^3. The solvent mask applied in the case of **7**•(toluene)$_{0.5}$ identified electron-containing voids in the unit cell with a total electron count of 196.9 electrons in a volume of 877.8 Å^3. The solvent mask applied in the case of ^{13}**8** identified an electron-containing void in the unit cell with a total electron count of 152.0 electrons in a volume of 1006.3 Å^3. In all cases, the voids are consistent with the presence of highly disordered hydrocarbon molecules in the crystal. The reported chemical formula and properties in ^{13}**6**, **7**•(toluene)$_{0.5}$, and ^{13}**8** do not include the disordered solvent, which was not modelled.

Elemental analysis. Elemental analyses were performed by the analytical service of London Metropolitan University, where samples were weighed using a Mettler Toledo high precision scale and analyzed using a ThermoFlash 2000.

Mass spectrometry. Samples for mass spectrometry were prepared by diluting 100 µL of a 1 mg/mL stock solution of analyte in THF with 900 µL of THF under inert atmosphere. The resulting mixture was filtered through glass filter paper before being injected into an electrospray ionization (ESI) equipped Waters RDa bench-top time of flight mass spectrometer provided by the mass spectrometry service of the University of Oxford. Mass spectra were simulated using the online Prot Pi Mass Spectrum Simulator.

Infrared spectroscopy. ATR-IR spectra were recorded on microcrystalline solids using a Bruker Alpha II under a dry N_2 atmosphere.

UV-Vis spectroscopy. Ultraviolet-visible (UV-Vis) electronic absorption spectra were recorded using a Mettler Toledo UV5Bio spectrophotometer. Samples were prepared under inert atmosphere and analyzed in 10 mm path length quartz J Young cuvettes.

Computational Methods. ORCA (version 6.1.0) was used for all quantum chemistry calculations.[7] The experimental coordinates obtained for **4**, ^{13}**6**, **7**•(toluene)$_{0.5}$, and ^{13}**8** by SC-XRD were loaded into Mercury (version 2024.2.0) and edited by removing K(~~2.2.2.~~crypt) (in the case of **4** and ^{13}**6**), solvent molecules (in the case of **7**•(toluene)$_{0.5}$), and disordered components, and normalizing H atoms, to obtain the initial input coordinates for **4**–K(~~2.2.2.~~crypt)$^{-}$, **6**–K(~~2.2.2.~~crypt)$^{-}$, **7**, and **8** (where **4**–K(~~2.2.2.~~crypt)$^{-}$ and **6**–K(~~2.2.2.~~crypt)$^{-}$ refer to the anionic components of **4** and **6** which do not include the

K(~~2.2.2.~~crypt) unit). Initial coordinates for CO_2 and CO were obtained from ^{13}**6** and ^{13}**8**, respectively. Coordinates were optimized with the r^{2}SCAN-3c composite electronic structure method.[8] Frequency calculations were performed on the resulting optimized coordinates, and no imaginary modes were identified. The optimized coordinates for **8** were used as the start point for a simultaneous, two-dimensional relaxed surface scan along the proposed reaction coordinate for the conversion of **7**+CO to **8**, in which the P–C1 (where C1 is the carbonyl carbon) is extended from 1.675 Å to 2.8 Å, while the P–C3 distance (where C3 is the secondary fluorenyl carbon that binds P in **7**, as displayed in Figure 4) is contracted from 3.53 Å to 1.97 Å. The optimized coordinates near the energy maximum, where the P–C1 distance is 2.425 Å and the P–C3 distance is 2.49 Å was used for a transition state search. A transition state, **TS**, was found; a frequency calculation of **TS** identified a single imaginary mode at –335 cm^{-1} which corresponds with the expected reaction coordinate. A single point energy calculation was performed on **TS** for a topological analysis, employing the PBE0 hybrid density functional, Grimme's D3 dispersion correction with Becke-Johnson damping (D3BJ), treatment of scalar relativistic effects with the exact two-component method (X2C), the x2c-TZVPPall basis set, and the X2C/J auxiliary basis set.[7, 8b, h, 9] Topological analysis was performed within MultiWFN (version 3.7).[10] A second single point energy calculation was performed on **TS** for Natural Bond Orbital (NBO) analysis, employing the PBE0 hybrid density functional, Grimme's D3 dispersion correction with Becke-Johnson damping (D3BJ), the def2-TZVP basis set, the RIJCOSX approximation, and the def2/J auxiliary basis set.[7] Optimized geometries were visualized in Mercury. Data were visualized in R (version 4.5.2). The following R packages were used in data visualization: ggplot2, tidyverse, gridExtra, ggtext, grid, scales, colorspace, dplyr, patchwork. NBO analysis was performed with the NBO program (version 7.0.7).[11] NBOs were visualized in Jmol (Version 16.3.49). In Figure 4B, positive contour lines (displayed as solid lines) are set at 0.001, 0.002, 0.004, 0.008, 0.01, 0.02, 0.04, 0.08, 0.1, 0.2, 0.4, 0.8, 1, 2, 4, 8, 10, 20, 40, 80, 100, 200, 400, 800, 1000, 2000, 4000, 8000, 10000, 20000, 40000, 80000, and negative contour lines (displayed as dashed lines) set at -0.001, -0.002, -0.004, -0.008, -0.01, -0.02, -0.04, -0.08, -0.1, -0.2, -0.4, -0.8, -1, 2, -4, -8, -10, -20, -40, -80, -100, -200, -400, -800, -1000, -2000, -4000, -8000, -10000, 20000, -40000, -80000.

Synthesis of 1, 2, and 3. Dimethyl 5-bromoisophthalate,[12] 1-bromo-3,5-bis(1-hydroxy-1-methylethyl)benzene,[13] 1-bromo-3,5-bis(1-chloro-1-methylethyl)benzene,[14] 2,5-dichloro-2,5-dimethyl-hexane[15], octamethyloctahydrodibenzofluorene,[1] the ketonic (M^sFluInd*)Br precursor (**a**),[1] the olefinic (M^sFluInd*)Br precursor (**b**),[1] (M^sFluInd*)Br[1], **1**, **2**, and **3** were prepared following established literature protocols following the overall scheme below (Figure S1).[3]

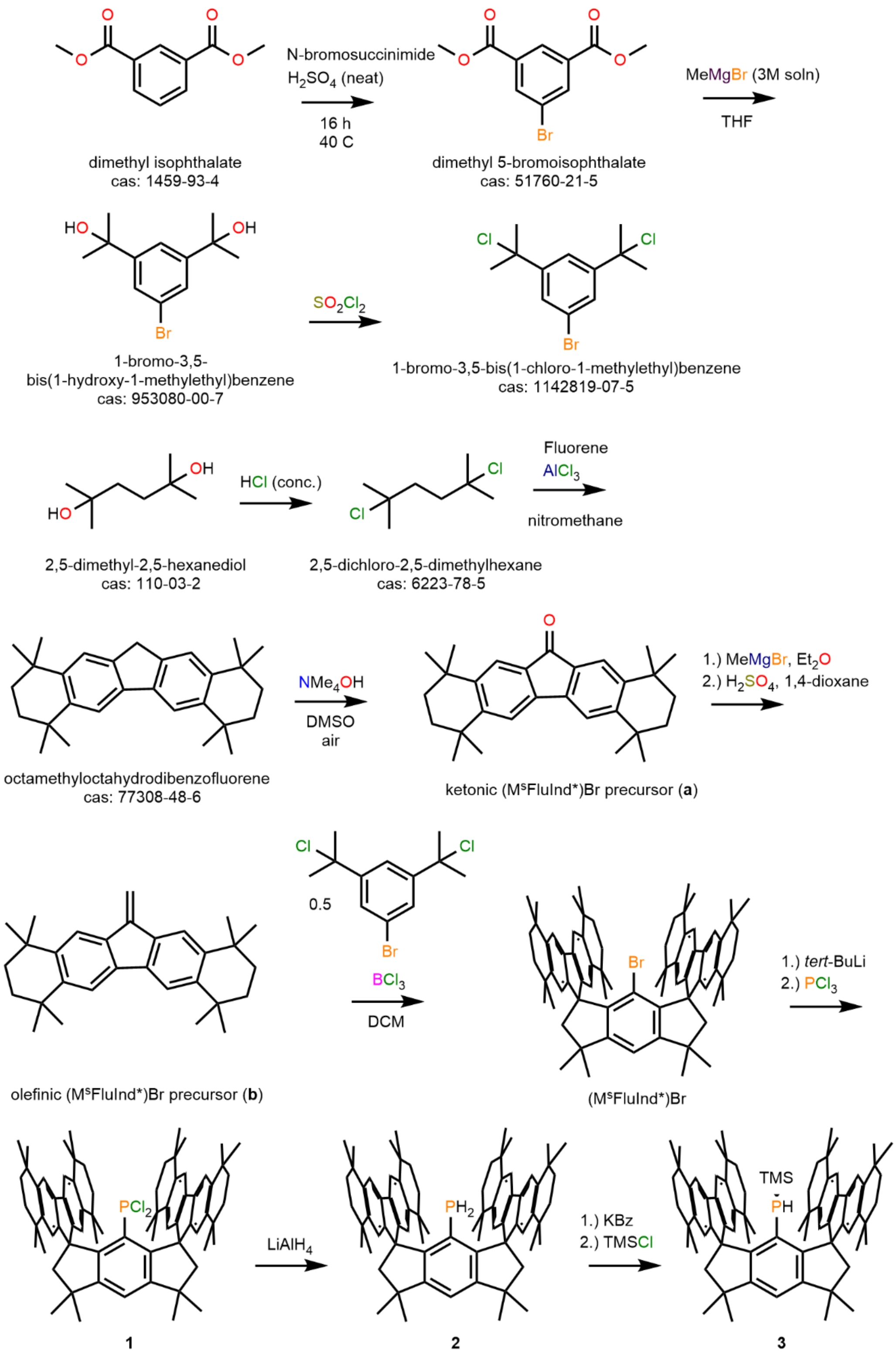

Figure S1. Synthetic route to **1**, **2**, and **3**.[1, 3]

2. Synthesis and characterization of novel compounds.

2.1 Synthesis of [K(~~2.2.2.~~crypt)][(M^{s}FluInd*)PH]•(THF)(pentane)$_{0.5}$ (4•(THF)(pentane)$_{0.5}$).

A solution of **2** (246 mg, 249 μmol) in benzene (8 mL) was added to a suspension of KBz (37 mg, 285 μmol) in benzene (0.6 mL) and stirred for 2 h. The reaction mixture was filtered through a glass filter pad into a vial containing ~~2.2.2.cryptand~~crypt (94 mg, 249 μmol). The resulting dark-green solution was stirred for 1 h before being stripped of solvent. The resulting residue was dissolved in THF, and the resulting solution layered with pentane. After 24 h, the vial was cooled to –30 °C for an additional 24 h. A large crop of dark-green crystals had formed in the vial, and the supernatant was decanted. The remaining solid was washed with pentane (3 × 0.6 mL) before being dried under vacuum. Yield: 289 mg (77%). Crystals of **4** suitable for SC-XRD were obtained by vapor diffusion of pentane into a solution of **4** in THF.

Elemental analysis, Found: C, 73.83; H, 9.17; N, 1.78%. **Calc.** for $C_{96.5}H_{140}KN_2O_7P$: C, 76.75; H, 9.34; N, 1.85%. Compound **4**•(THF)(pentane)$_{0.5}$ is highly sensitive and elemental analyses were consistently unsuccessful; best results are provided. Bulk purity of freshly prepared material was determined by ^{1}H, ^{13}C{^{1}H}, and ^{31}P{^{1}H} NMR spectroscopy.

HR-ESI-MS (m/z) [(M^{s}FluInd*)PH$_3$]$^+$ 987.705 (calc. 987.693).

HR-ESI-MS (m/z) [K(~~2.2.2.~~crypt)]$^+$ 415.229 (calc. 415.220).

^{1}H NMR (400 MHz, C_6D_6): δ = 7.76 (s, 4H), 7.63 (s, 4H), 6.69 (s, 1H), 3.04 (s, 12H), 2.99 – 2.91 (m, 12H), 2.49 (s, 4H), 2.06 – 1.99 (m, 12H), 1.90 – 1.60 (m, 28H), 1.53 – 1.35 (m, 48H) ppm.

^{13}C{^{1}H} NMR (101 MHz, C_6D_6): δ = 155.2, 142.4, 139.7, 138.2, 122.7, 115.9, 70.4, 67.7, 66.9, 63.2, 54.4, 42.3, 36.5, 36.4, 34.9, 34.7, 33.5, 33.1, 32.7, 32.7, 32.5 ppm.

^{31}P NMR (162 MHz, C_6D_6): δ = –69.9 (d, $^{1}J_{PH}$ = 154.2 Hz) ppm.

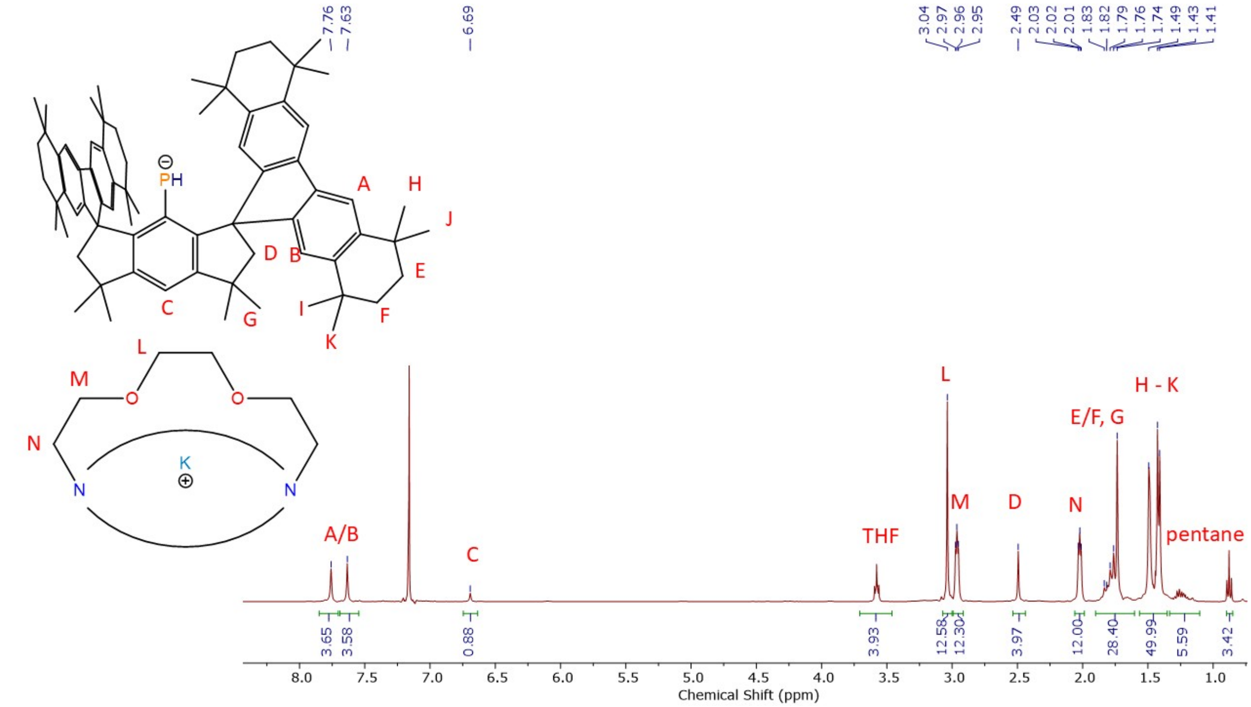

Figure S2. ^{1}H NMR spectrum (C_6D_6, 400 MHz) of **4**•(THF)(pentane)$_{0.5}$ at room temperature.

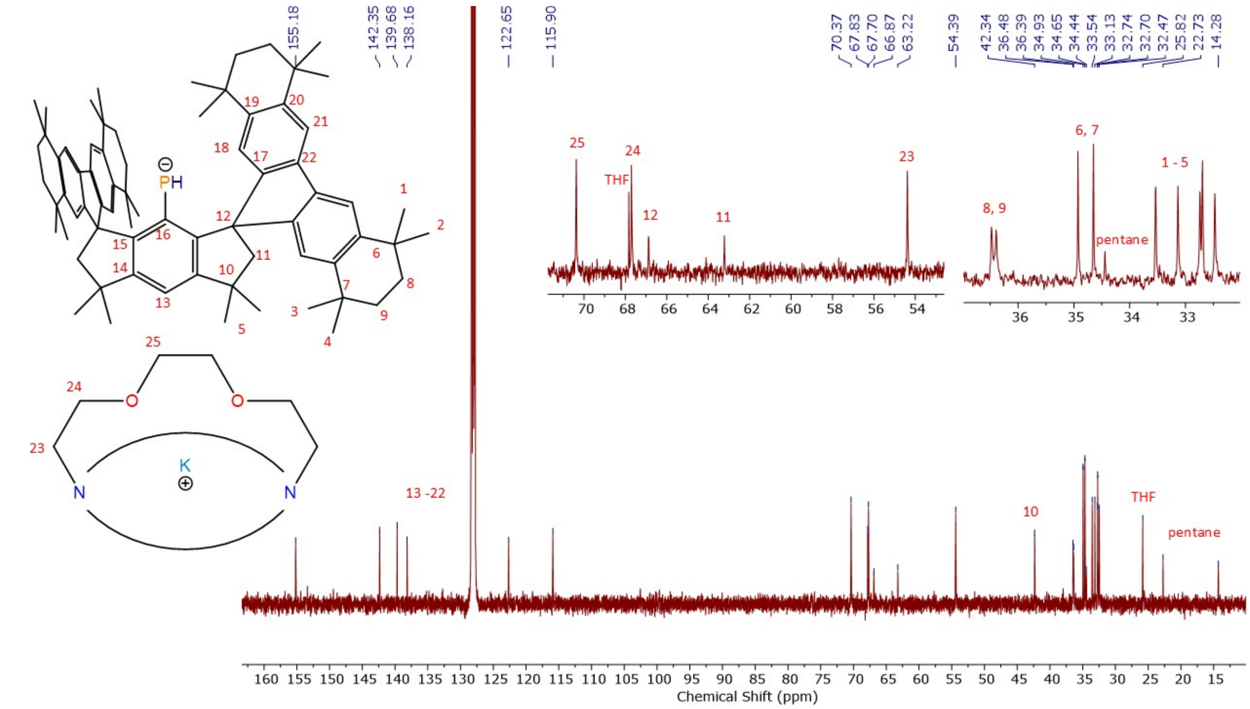

Figure S3. $^{13}C\{^{1}H\}$ NMR spectrum (C_6D_6, 101 MHz) **4**•(THF)(pentane)$_{0.5}$ at room temperature.

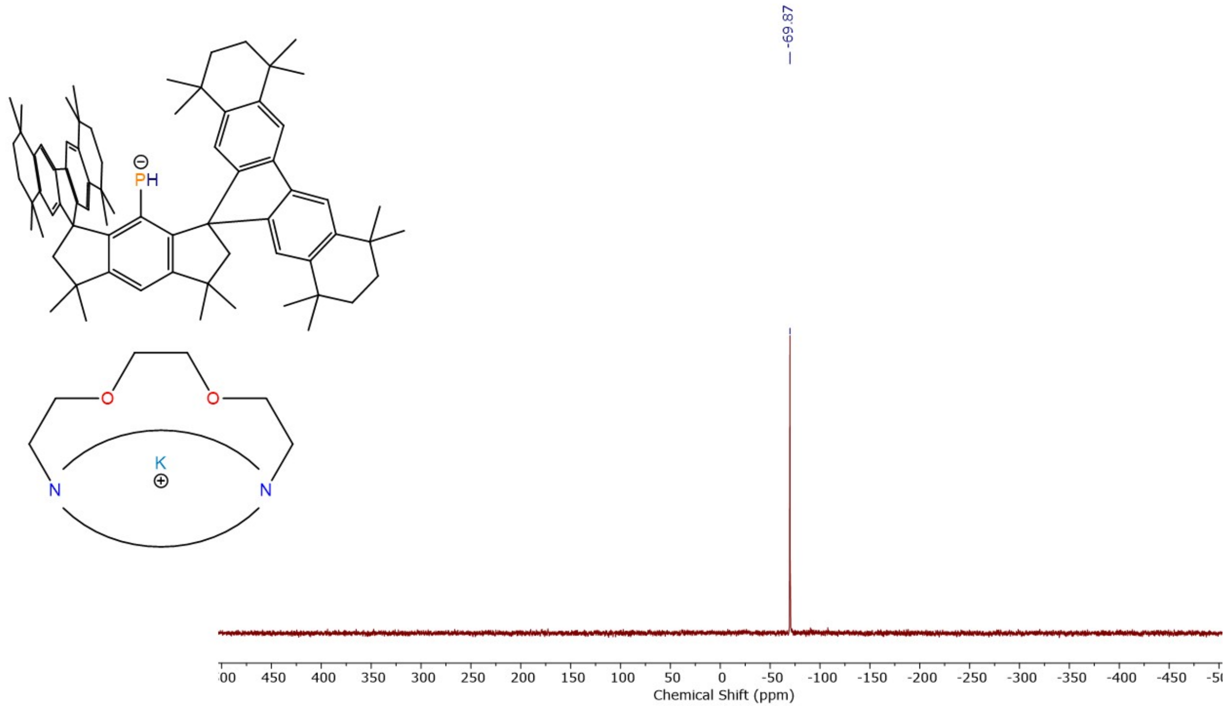

Figure S4. $^{31}P\{^{1}H\}$ NMR spectrum (C_6D_6, 162 MHz) of **4**•(THF)(pentane)$_{0.5}$ at room temperature.

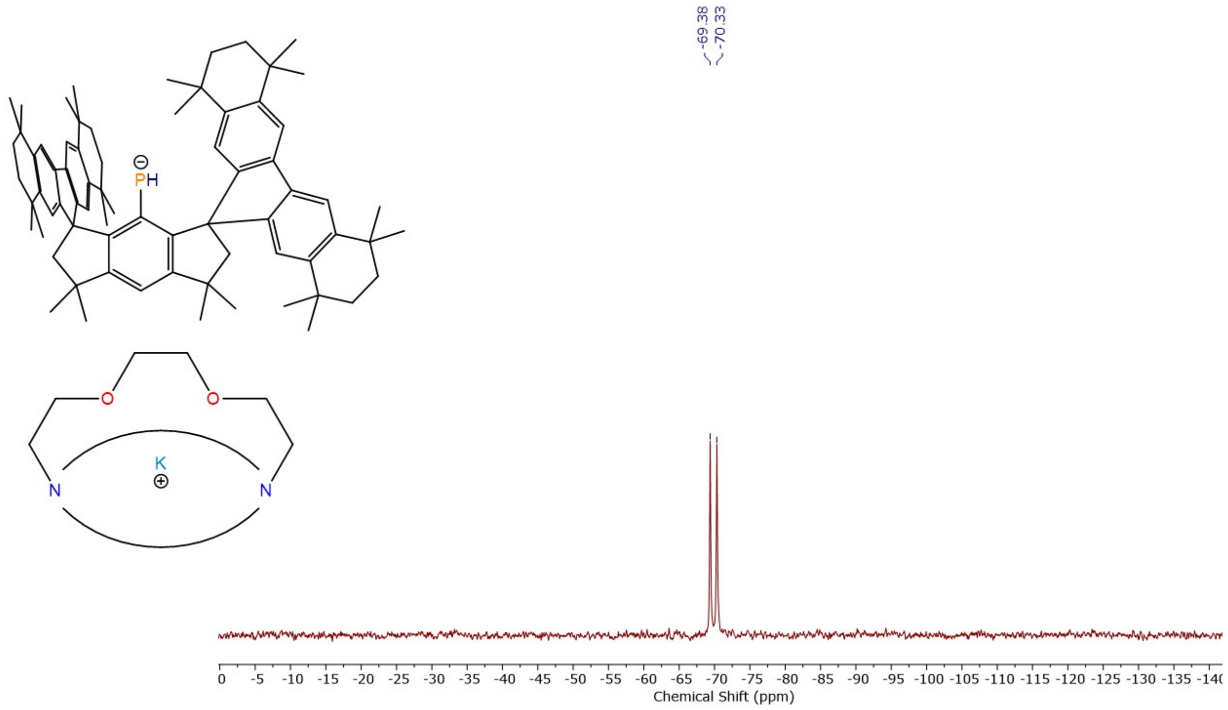

Figure S5. ^{31}P NMR spectrum (C_6D_6, 162 MHz) of **4**•(THF)(pentane)$_{0.5}$ at room temperature.

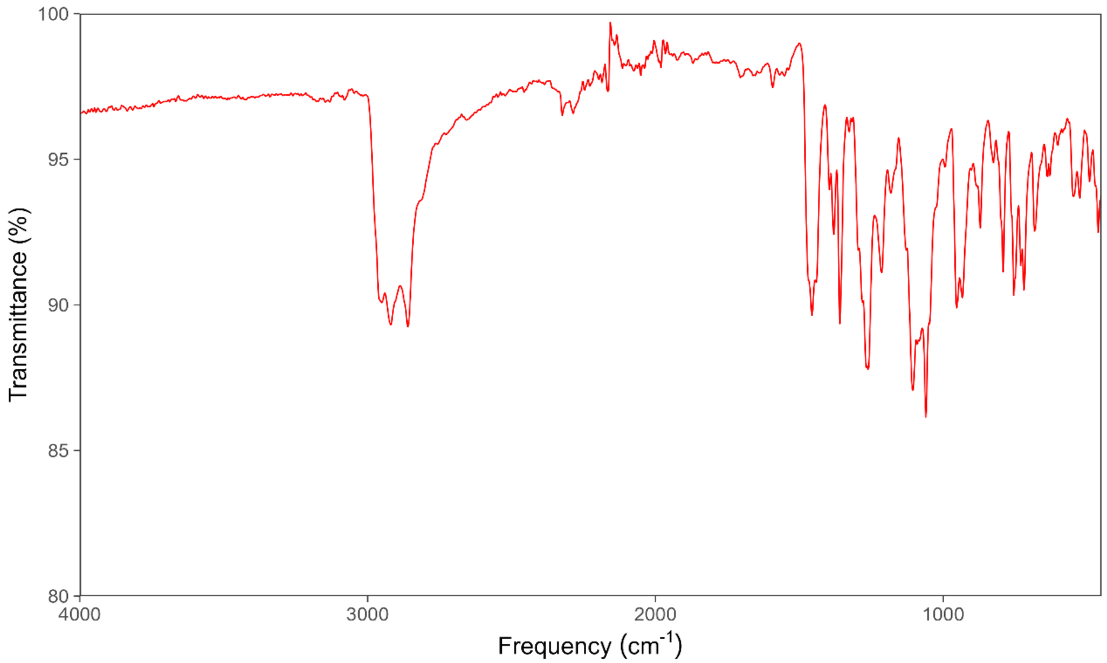

Figure S6. Experimental IR spectrum of **4**•(THF)(pentane)$_{0.5}$.

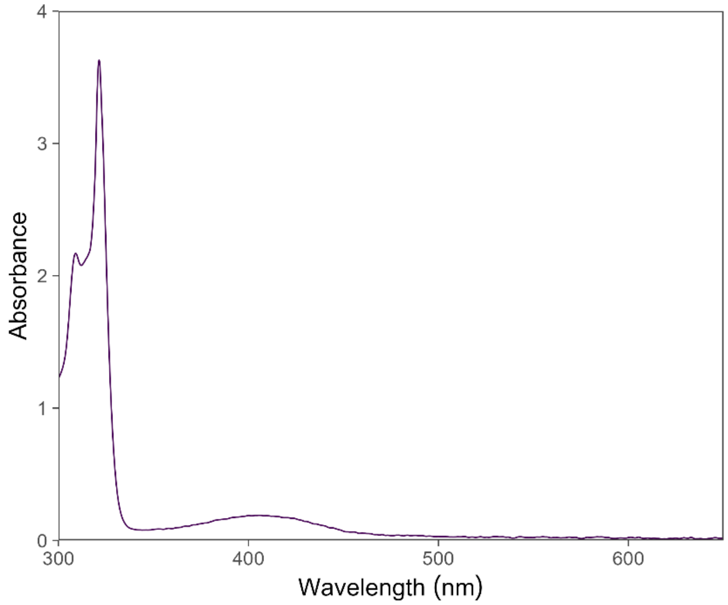

Figure S7. Experimental UV-Vis spectrum of **4**•(THF)(pentane)$_{0.5}$ (83 μM).

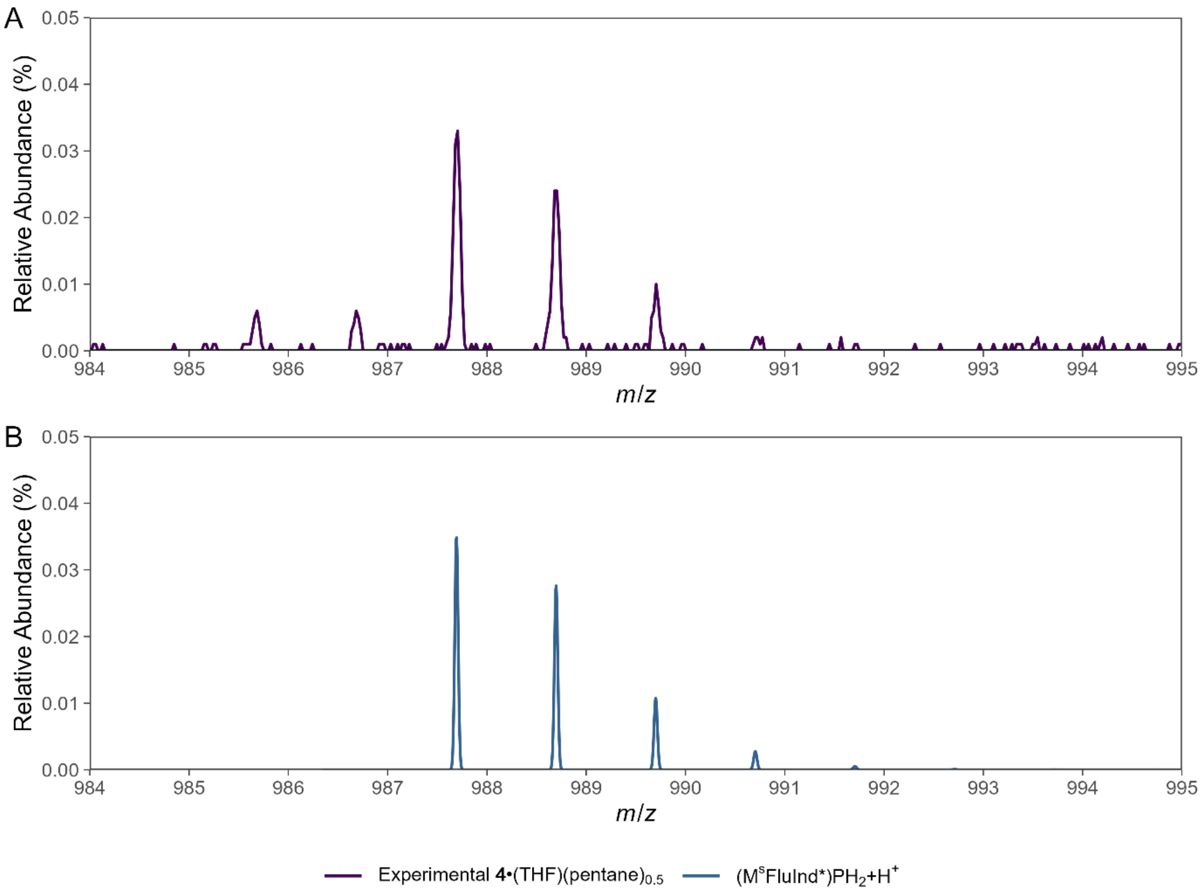

Figure S8. (A) Experimental HR-ESI-MS spectrum for **4**•(THF)(pentane)$_{0.5}$. (B) Simulated HR-ESI-MS spectrum for (M^{s}FluInd*)PH_2+H^+.

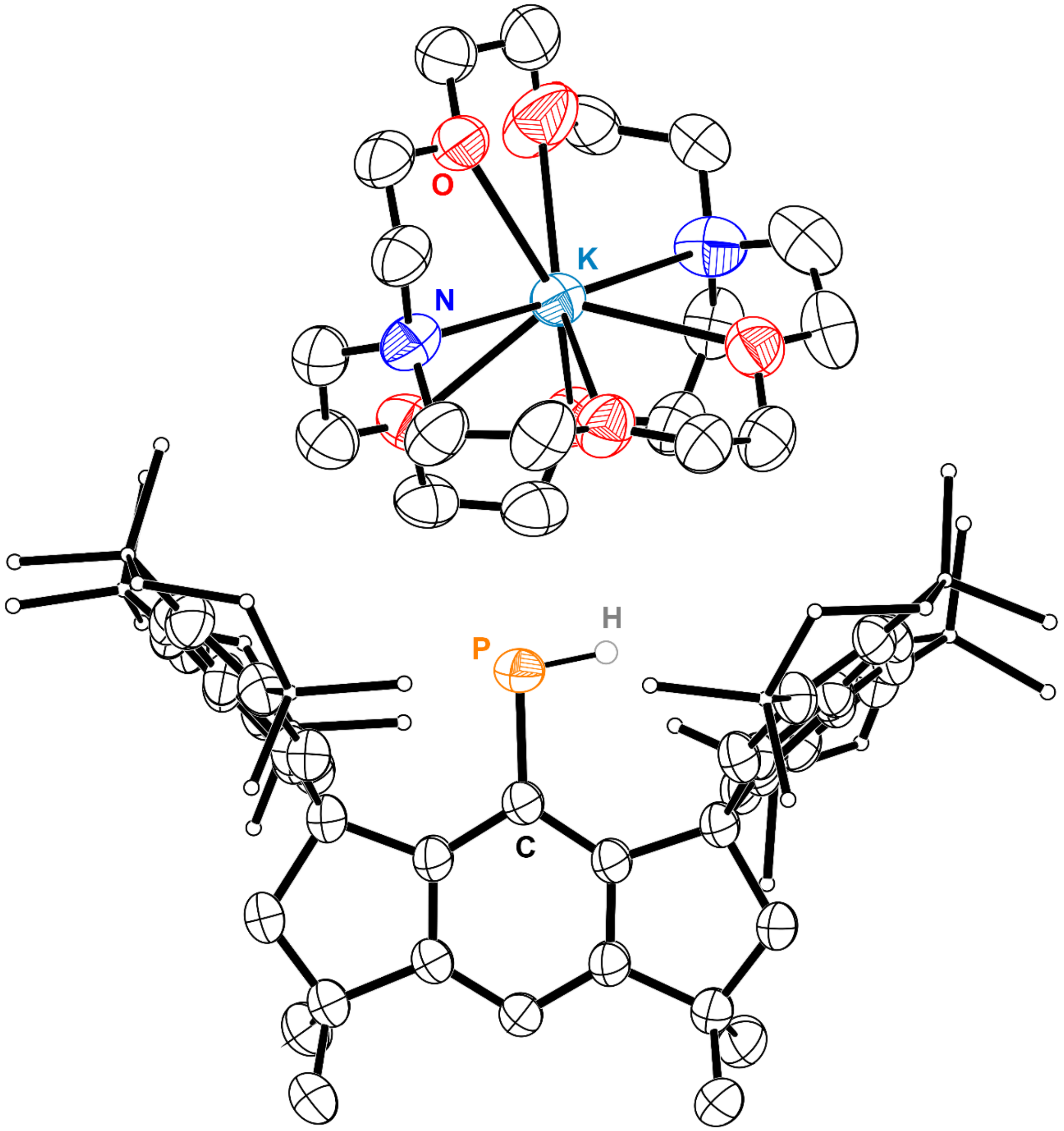

Figure S9. Thermal ellipsoid plot (50% probability) of **4**. C-bound H atoms and disordered components are omitted for clarity. Select alkyl C atoms are shown as spheres of arbitrary size for clarity. Color code: P orange, C black, N blue, K teal, H grey.

2.2 Synthesis of [K(2.2.2.crypt)][(M^sFluInd*)PO$_2$H] (5).

A suspension of **4**•(THF)(pentane)$_{0.5}$ (44 mg, 29 µmol) in benzene (0.6 mL) was filtered and transferred into a J-Young tube. The sample was degassed *via* freeze-pump-thaw three times before being frozen in a liquid N_2 cooling bath. An excess of gaseous N_2O was transferred to the tube. The reaction mixture was allowed to warm to room temperature, and the tube was inverted three times. The solvent was stripped to afford **5** as a colorless solid. Yield: 36 mg (86%). Crystals suitable for X-ray diffraction were grown from a mixture of THF/pentane.

Elemental analysis, Found: C, 74.31; H, 8.87; N, 1.95%. **Calc.** for $C_{90}H_{126}KN_2O_8P$: C, 75.38; H, 8.86; N, 1.95%.

HR-ESI-MS (m/z) [5–K(2.2.2.crypt)]$^{-}$ 1017.661 (calc. 1017.668).

^{1}H NMR (500 MHz, C_6D_6): δ = 7.76 (br s, 4H), 7.66 (br s, 4H), 7.47 (br s, 1H), 3.17 (br s, 12H), 3.11 (br s, 12H), 2.31 (br s, 4H), 2.18 (br s, 12H), 1.85 – 1.65 (br m, 16H), 1.59 – 1.29 (br m, 60H) ppm.

^{13}C{^{1}H} NMR (126 MHz, C_6D_6): δ = 155.9, 142.6, 140.5, 137.1, 123.5, 116.2, 70.3, 67.8, 66.4, 65.3, 55.0, 41.2, 36.3, 35.1, 34.7, 33.3, 32.9, 32.5, 32.4, 32.1 ppm.

^{31}P NMR (202 MHz, C_6D_6): δ = 1.2 (d, $^{1}J_{PH}$ = 476.1 Hz) ppm.

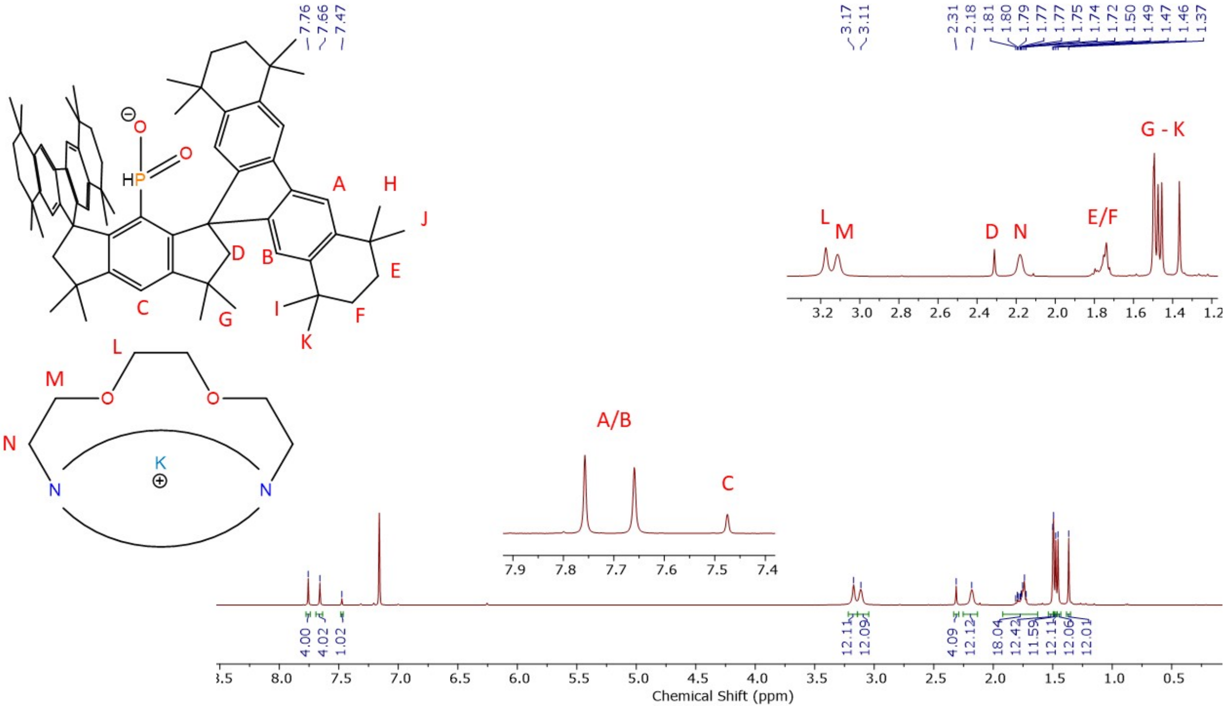

Figure S10. ^{1}H NMR spectrum (C_6D_6, 500 MHz) of **5** at room temperature.

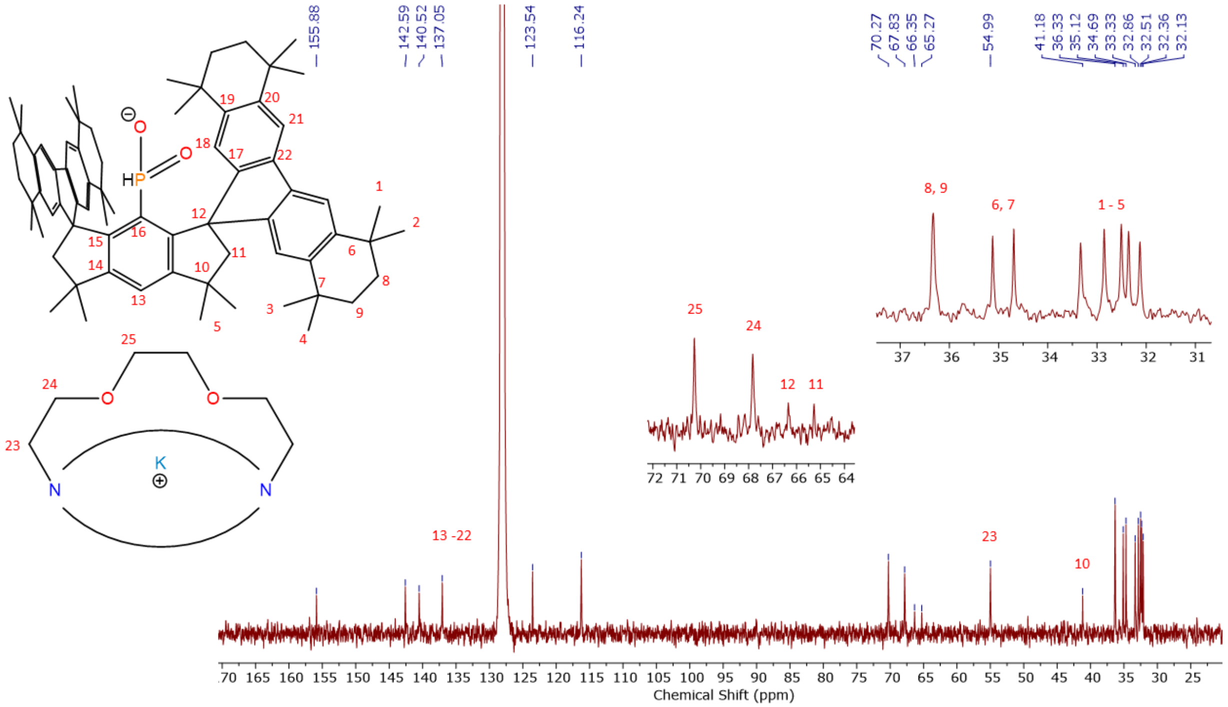

Figure S11. $^{13}C\{^{1}H\}$ NMR spectrum (C_6D_6, 126 MHz) **5** at room temperature.

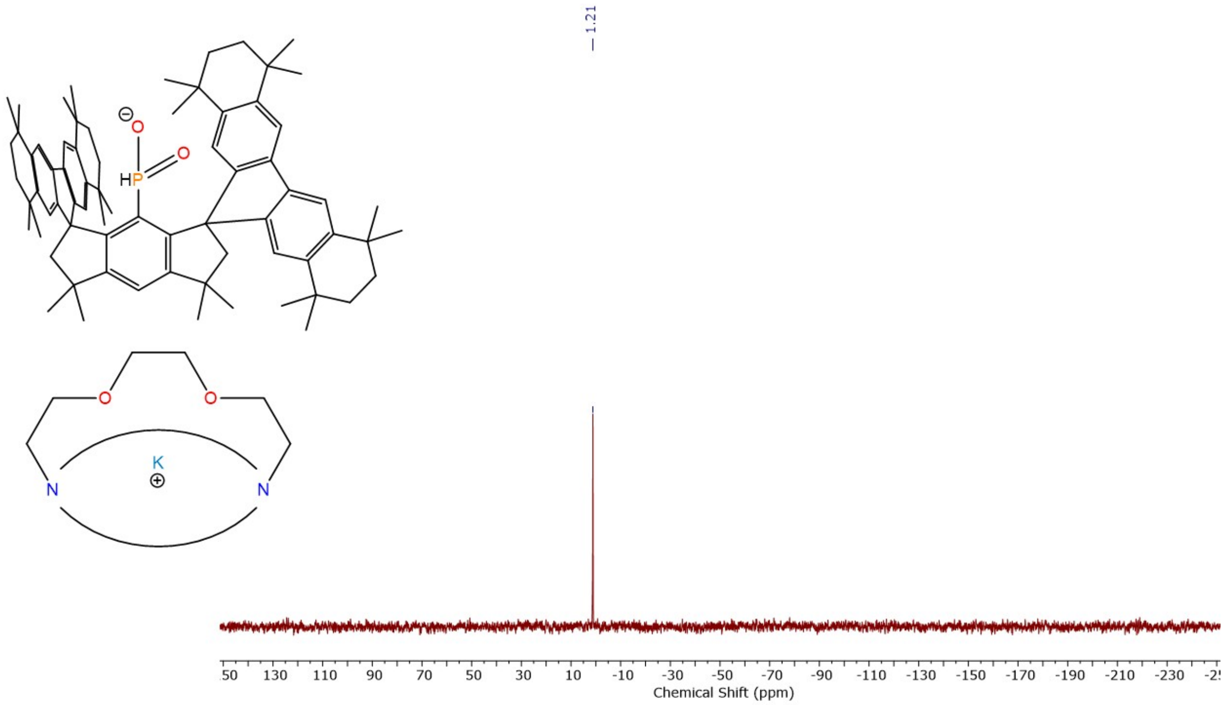

Figure S12. $^{31}P\{^{1}H\}$ NMR spectrum (C_6D_6, 202 MHz) of **5** at room temperature.

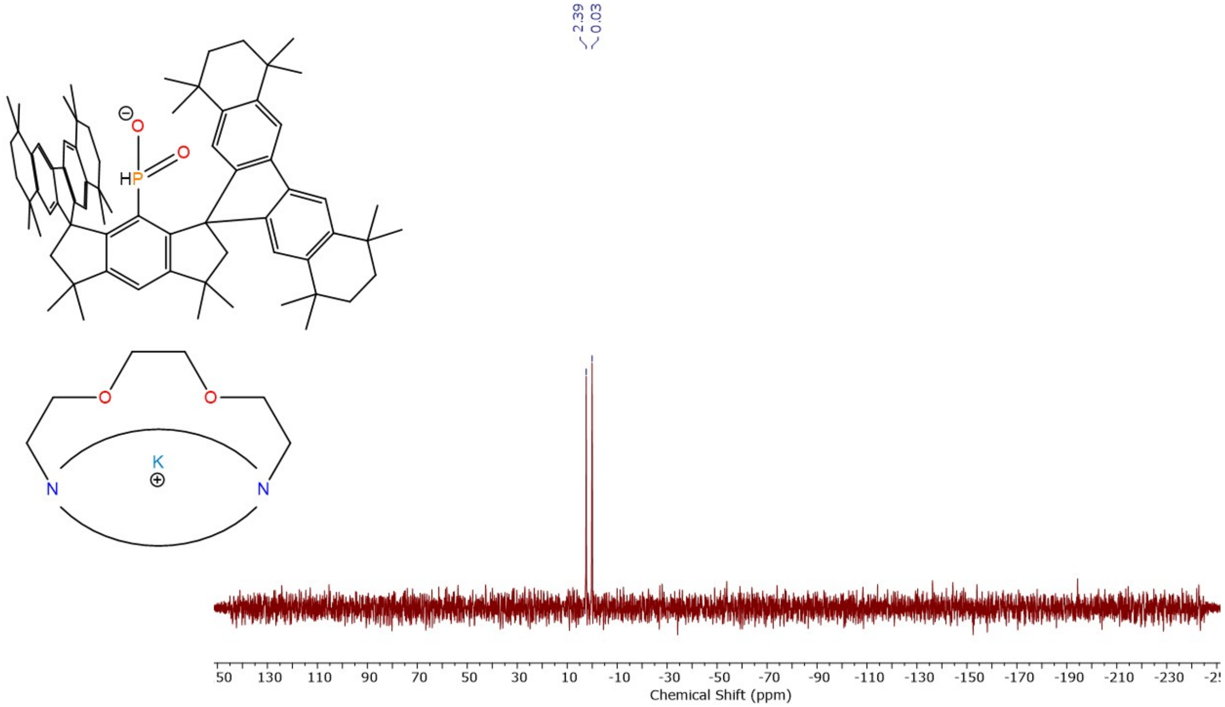

Figure S13. ^{31}P NMR spectrum (C_6D_6, 202 MHz) of **5** at room temperature.

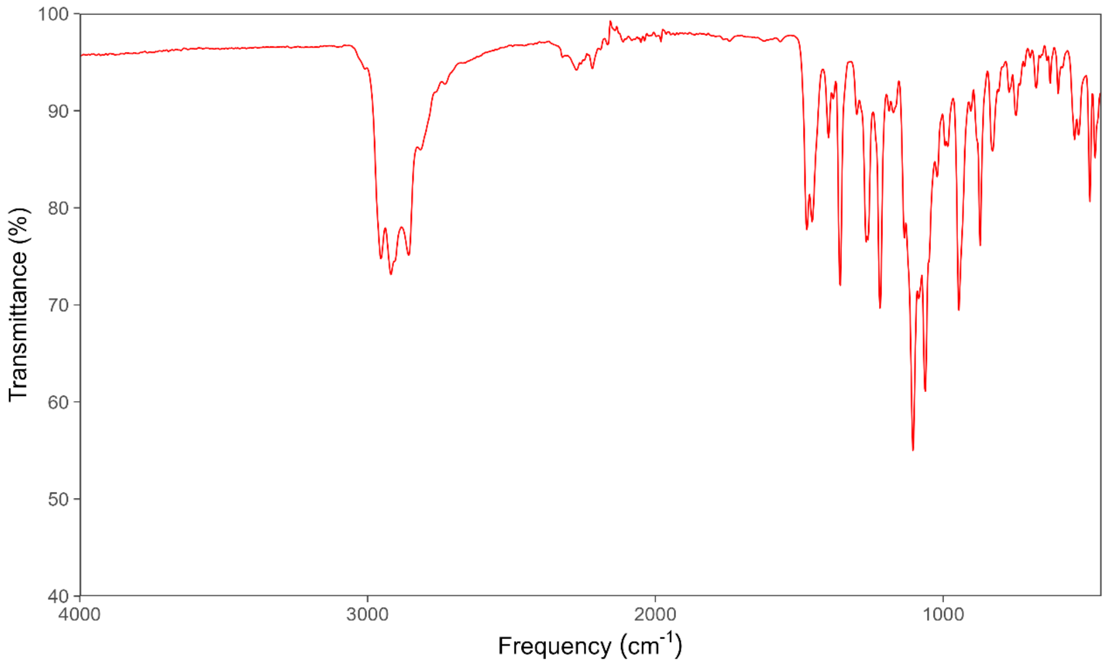

Figure S14. Experimental IR spectrum of **5**.

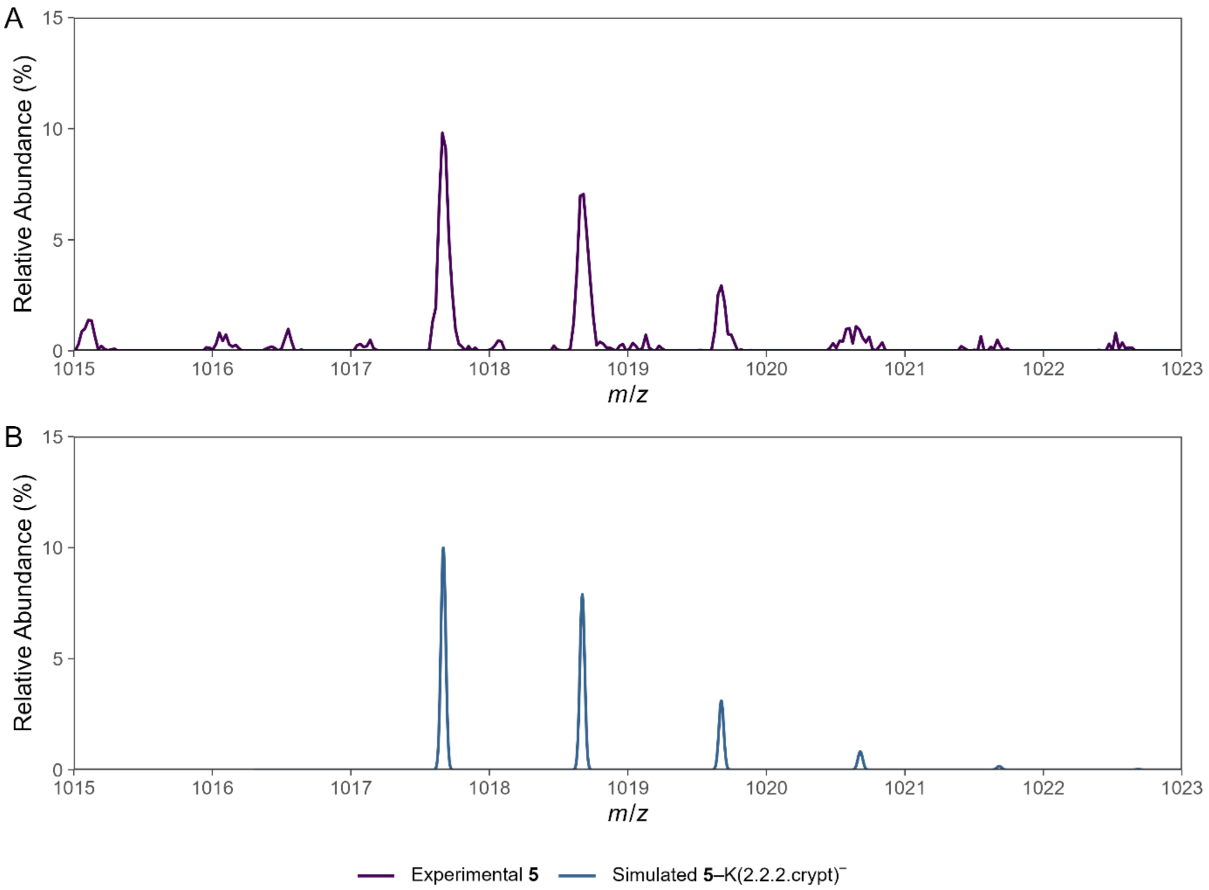
A
Relative Abundance (%)
15
10
5
0
1015
1016
1017
1018
1019
1020
1021
1022
1023
m/z
B
Relative Abundance (%)
15
10
5
0
1015
1016
1017
1018
1019
1020
1021
1022
1023
m/z
Experimental 5
Simulated 5–K(2.2.2.crypt)⁻

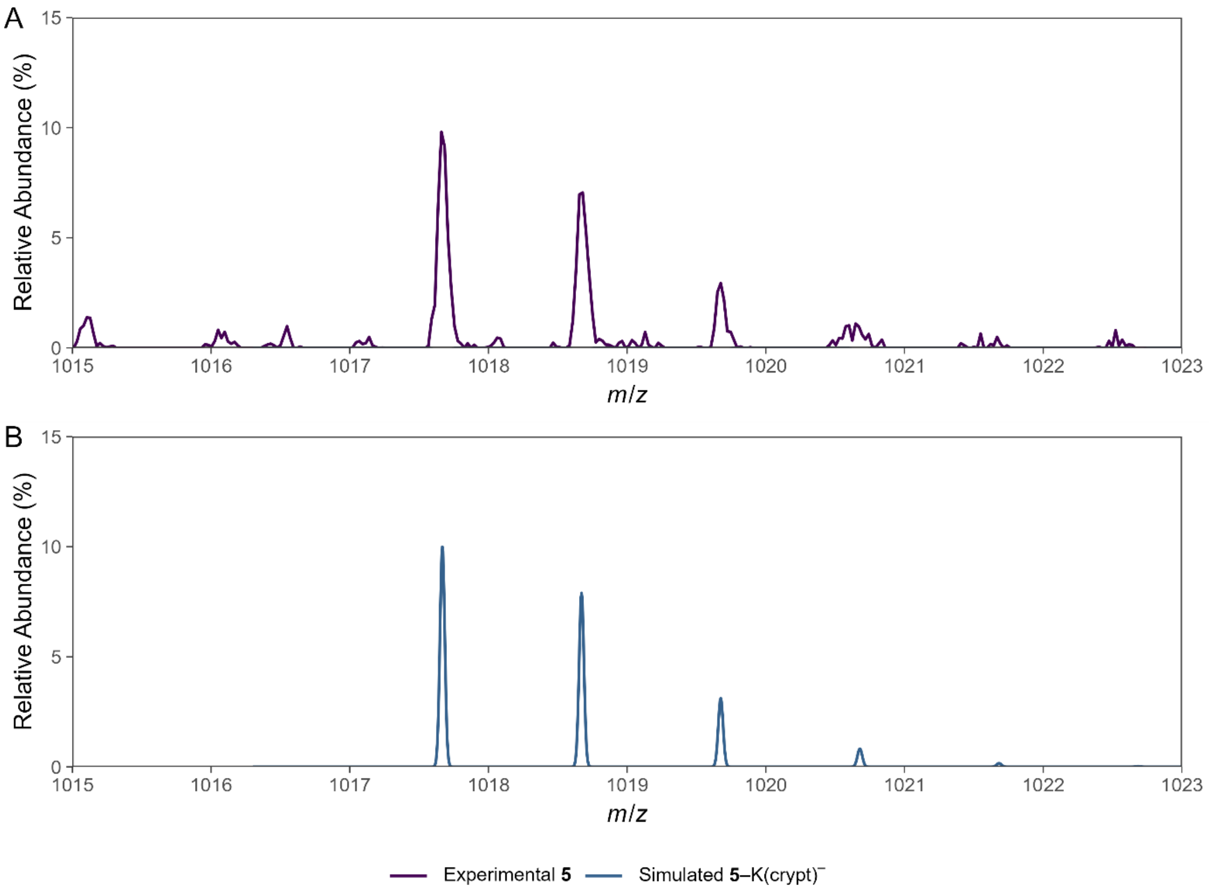

Figure S15. (A) Experimental HR-ESI-MS spectrum of **5**. (B) Simulated HR-ESI-MS spectrum for **5**–K(~~2.2.2.~~crypt)⁻.

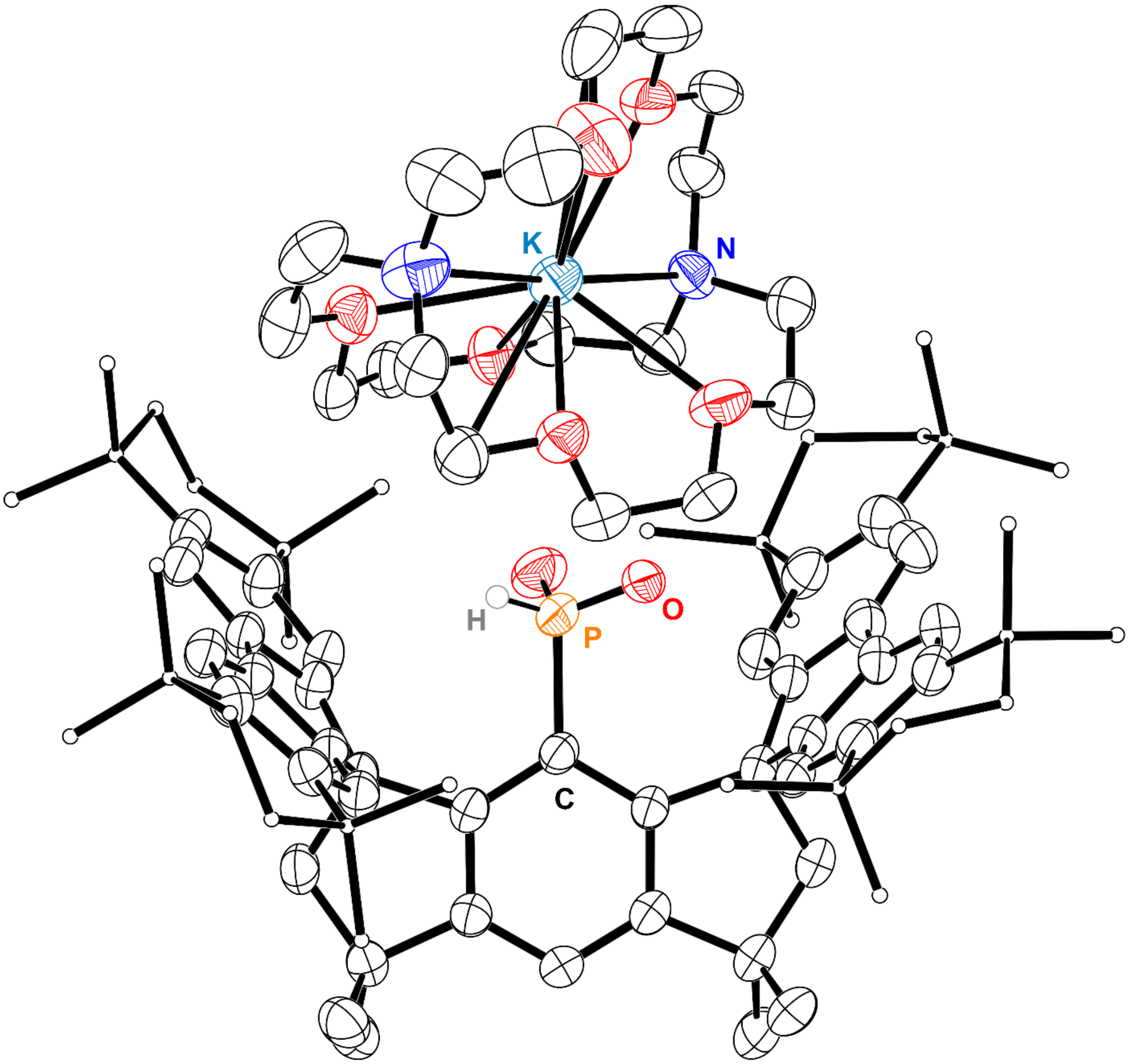

Figure S16. Thermal ellipsoid plot (50% probability) of **5**. C-bound H atoms and disordered components are omitted for clarity. Select alkyl C atoms are shown as spheres of arbitrary size for clarity. Color code: P orange, C black, N blue, K teal, H grey, O red.

2.3 Synthesis of [K(~~2.2.2.~~crypt)][(M^sFluInd*)P(CO_2)H] (^{13}6) *in situ*.
A suspension of **4**•(THF)(pentane)$_{0.5}$ (48 mg, 32 μmol) in benzene was filtered and transferred into a J-Young tube. The sample was degassed *via* freeze-pump-thaw three times. Gaseous $^{13}CO_2$ (1 atm) was transferred to the sample at room temperature. The tube was inverted three times. The resulting sample was analyzed by ^{1}H, ^{31}P{^{1}H}, ^{31}P, and ^{13}C{^{1}H} NMR, revealing the presence of both ^{13}**6** and **2**. The solvent was stripped to afford a solid mixture containing ^{13}**6** and **2**, which was analyzed by IR spectroscopy and HR-ESI-MS. Attempts to isolate ^{13}**6** as a pure bulk material were unsuccessful, and all bulk samples contained significant amounts of the decomposition product, **2**. Crystals of ^{13}**6** suitable for X-ray diffraction were grown from a mixture of pentane/toluene.

Note: ^{13}**6** was generated *in situ* and was not isolated as a pure, bulk solid due to partial decomposition to form **2**.

HR-ESI-MS (m/z) [6–K]$^-$ 1406.939 (calc. 1406.933).

^{1}H NMR (400 MHz, C_6D_6): δ = 7.77 (s, 4H), 7.70 – 7.55 (br m, 4H), 7.44 (s, 1H), 3.27 (s, 12H), 3.16 (br s, 12H), 2.24 (br s, 12H), 1.90 – 1.76 (br m, 8H), 1.74 – 1.65 (br m, 8H), 1.57 – 1.35 (br m, 65 H) ppm.

^{13}C{^{1}H} NMR (101 MHz, C_6D_6): δ = 168.0, 156.8, 156.2, 154.2, 141.1, 138.8, 137.3, 122.3, 117.4, 116.3, 116.1, 70.5, 68.1, 66.4, 64.2, 55.2, 42.0, 36.3, 36.3, 35.2, 34.8, 33.4, 33.3, 32.9, 32.4, 32.3, 32.3 ppm.

^{31}P NMR (162 MHz, C_6D_6): δ = –85.5 (d, $^1J_{PH}$ = 221.7 Hz) ppm.

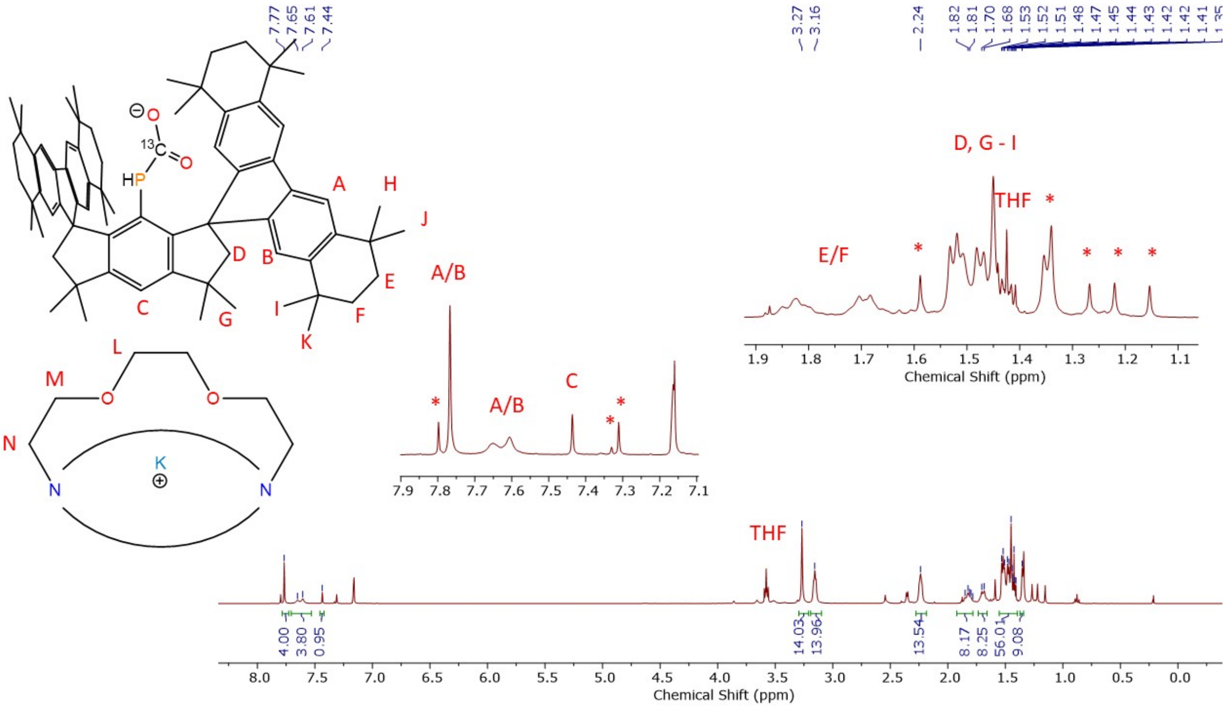

Figure S17. ^{1}H NMR spectrum (C_6D_6, 400 MHz) of a mixture of ^{13}**6** and **2** at room temperature, formed *in situ* by treatment of **4**•(THF)(pentane)$_{0.5}$ with $^{13}CO_2$. Signals arising from compound **2** are labelled with an asterisk.

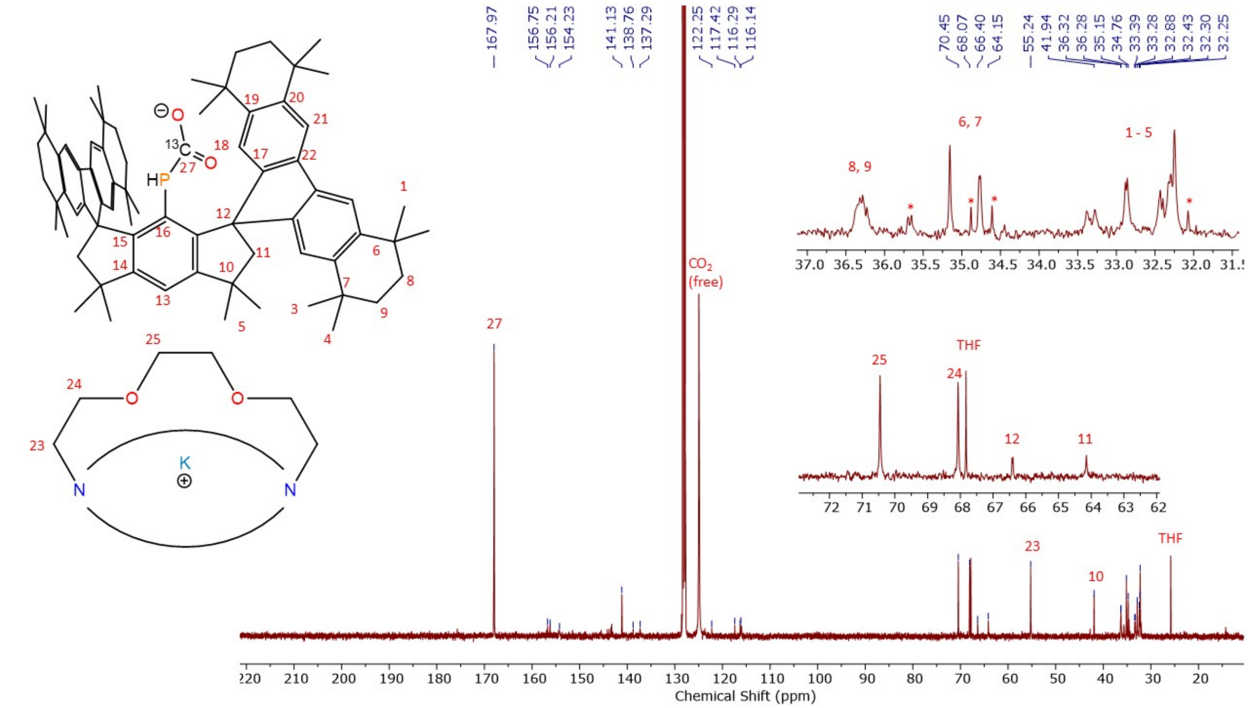

Figure S18. $^{13}C\{^1H\}$ NMR spectrum (C_6D_6, 101 MHz) of a mixture of ^{13}**6** and **2** at room temperature, formed *in situ* by treatment of **4**•(THF)(pentane)$_{0.5}$ with $^{13}CO_2$. Signals arising from compound **2** are labelled with an asterisk.

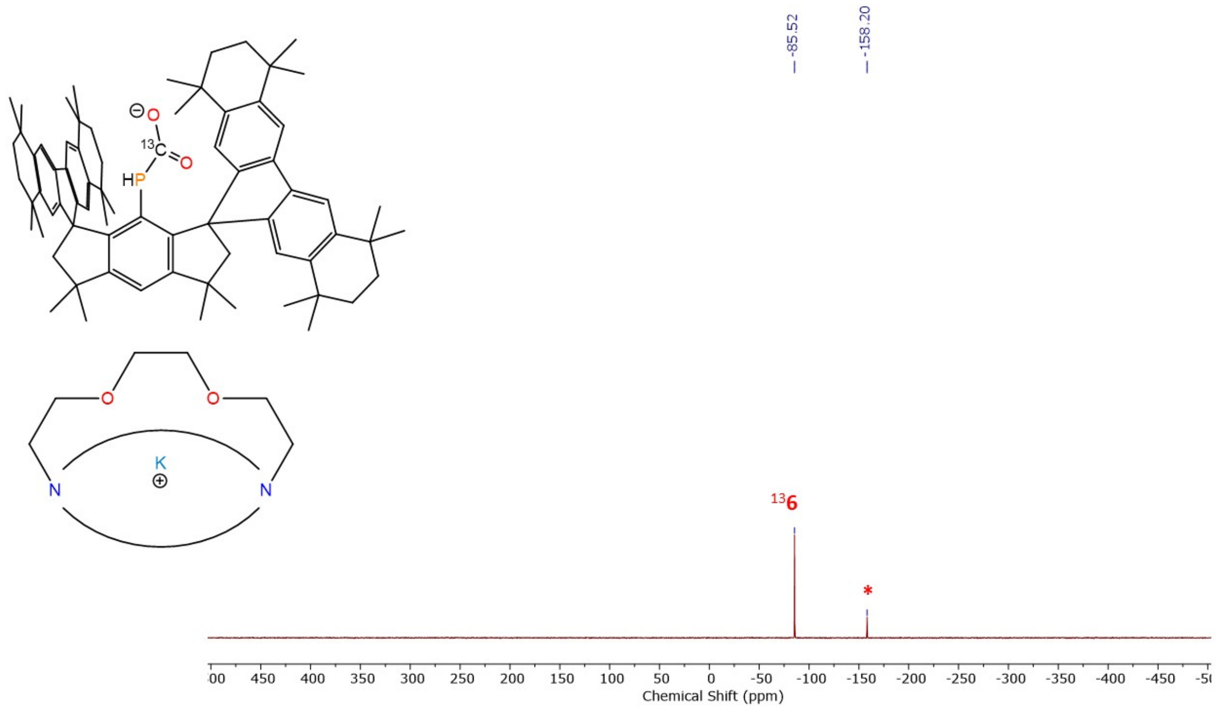

Figure S19. $^{31}P\{^{1}H\}$ NMR spectrum (C_6D_6, 162 MHz) of a mixture of ^{13}**6** and **2** at room temperature, formed *in situ* by treatment of **4**•(THF)(pentane)$_{0.5}$ with $^{13}CO_2$. The signal arising from compound **2** is labelled with an asterisk.

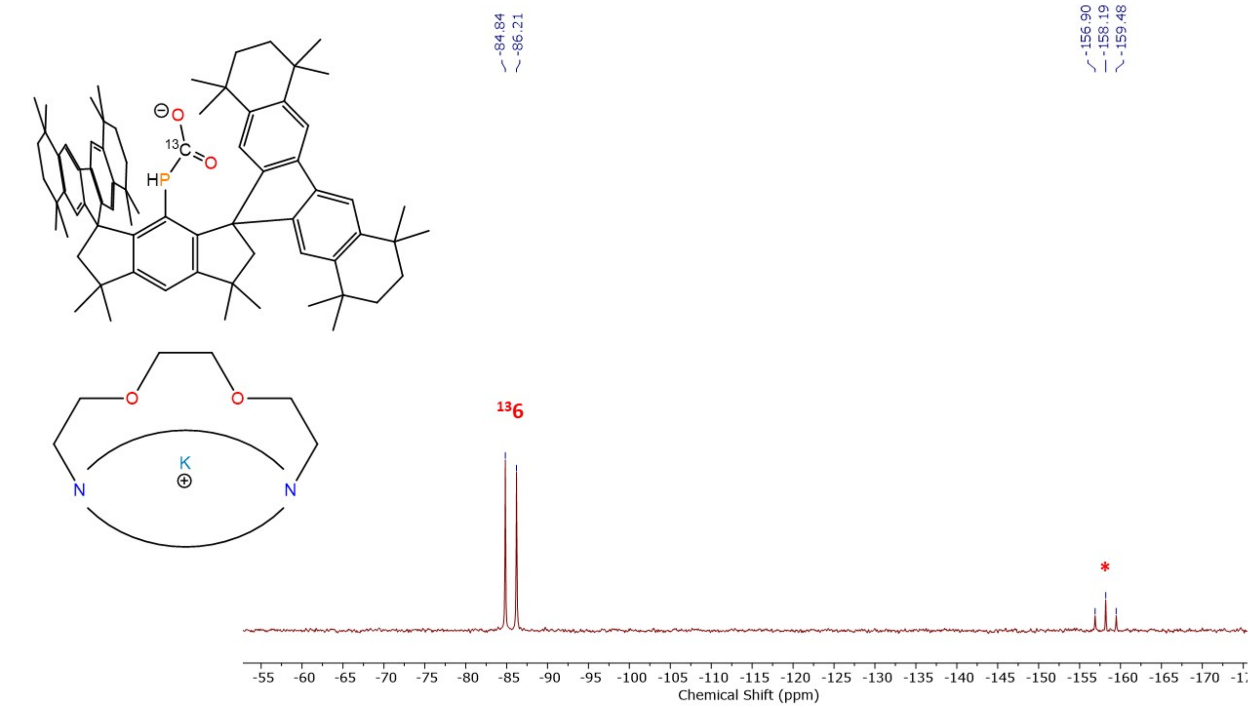

Figure S20. ^{31}P NMR spectrum (C_6D_6, 162 MHz) of a mixture of ^{13}**6** and **2** at room temperature, formed *in situ* by treatment of **4**•(THF)(pentane)$_{0.5}$ with $^{13}CO_2$. The signal arising from compound **2** is labelled with an asterisk.

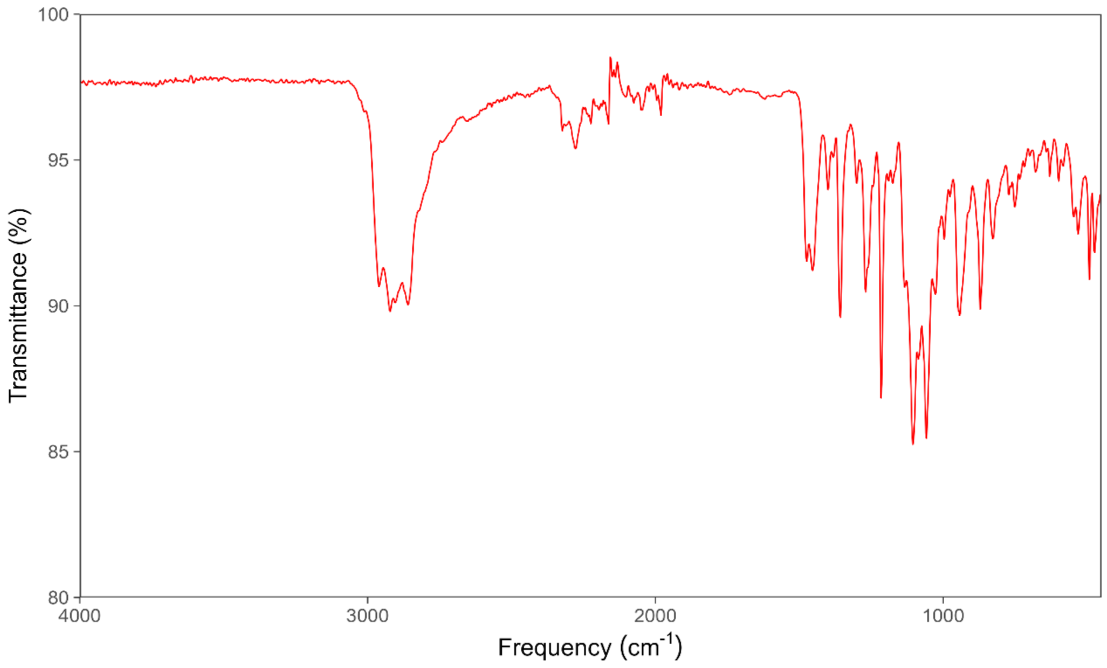

Figure S21. Experimental IR spectrum of a mixture of ^{13}**6** and **2** at room temperature, formed by treatment of **4**•(THF)(pentane)$_{0.5}$ with $^{13}CO_2$.

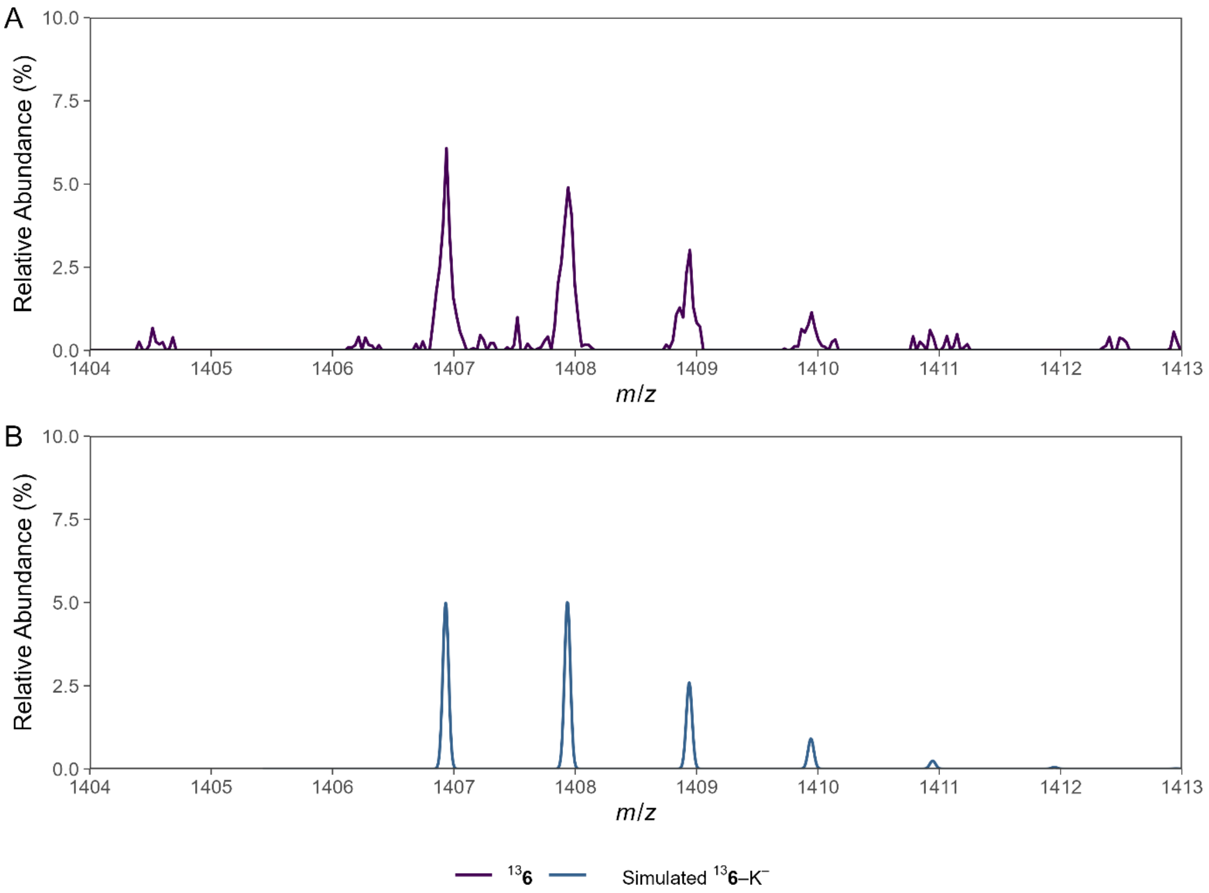

Figure S22. (A) Experimental HR-ESI-MS spectrum of a mixture of ^{13}**6** and **2** at room temperature, formed by treatment of **4**•(THF)(pentane)$_{0.5}$ with $^{13}CO_2$. (B) Simulated HR-ESI-MS spectrum for ^{13}**6**–K^{-}.

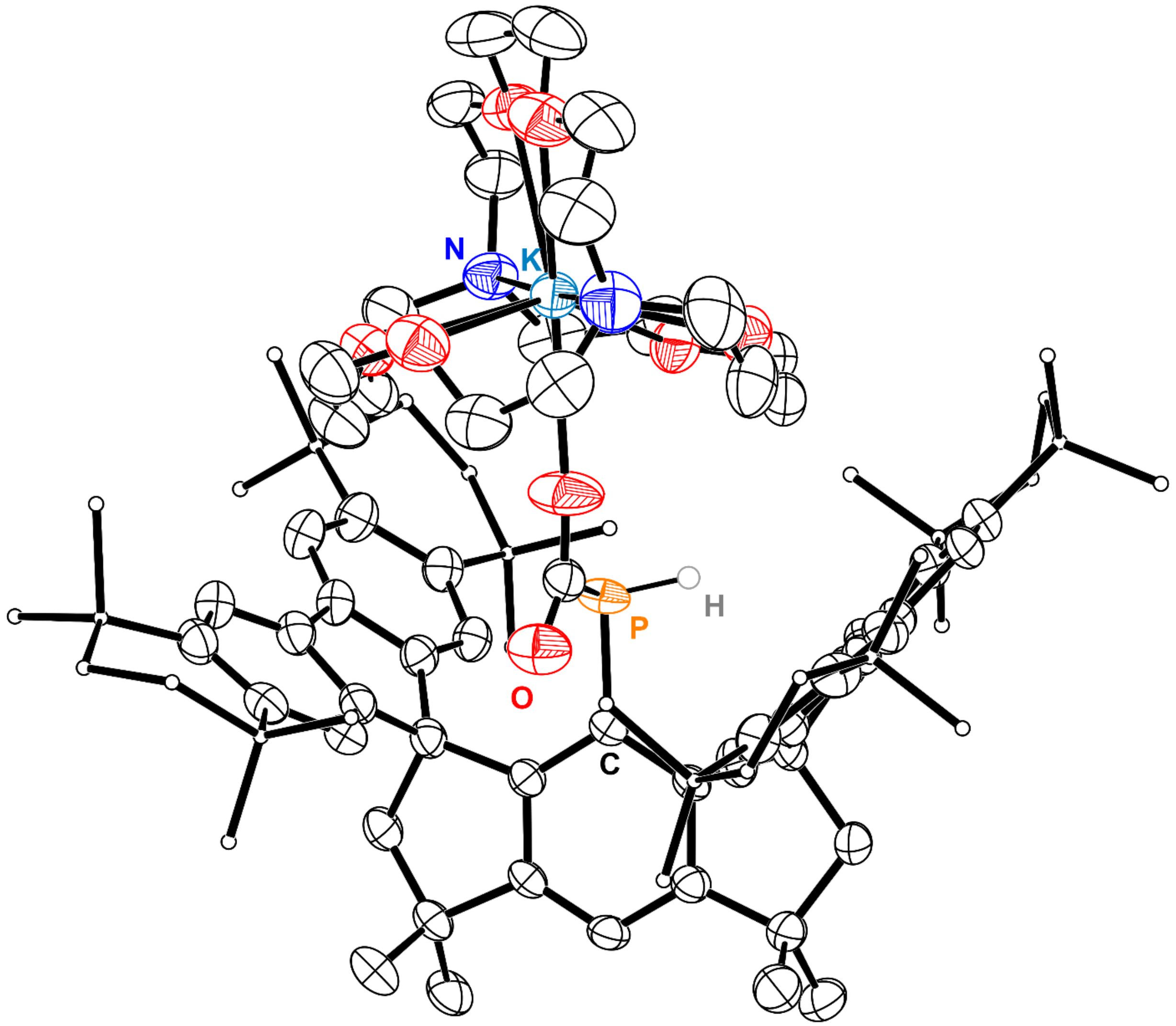

Figure S23. Thermal ellipsoid plot (50% probability) of [13]**6**. C-bound H atoms and disordered components are omitted for clarity. Select alkyl C atoms are shown as spheres of arbitrary size for clarity. Color code: P orange, C black, N blue, K teal, H grey, O red.

2.4 Synthesis of (M^sFluInd*)P (7).

Method A. A solid mixture of **3**•$(Et_2O)_2$ (33 mg, 27.3 μmol) and KBz (6.1 mg, 47 μmol) was suspended in C_6D_6 (1 mL) and stirred for 2 h. The reaction mixture was filtered through a glass filter pad and transferred to a J-Young tube. The sample was degassed via freeze-pump-thaw before being frozen in a liquid N_2 cooling bath. An excess of gaseous N_2O was transferred to the tube. The reaction mixture was allowed to warm to room temperature, and the tube was inverted three times. The resulting sample was analyzed with ^1H and $^{31}P\{^1H\}$ NMR spectroscopy, confirming the quantitative formation of **7**, *in situ* (Supplementary Figures S27, S28). The solvent was then removed and the solid was dissolved in hexane, filtered, and stripped of solvent to afford **7** as a yellow-orange powder. Yield: 16 mg (59%). Crystals of **7**•$(toluene)_{0.5}$ suitable for X-ray diffraction were grown from a concentrated mixture of hexane/toluene.

Method B. A vial was loaded with **1** (300 mg, 284 μmol), KC_8 (78 mg, 577 μmol), and a stir bar before being cooled to –30 °C. An aliquot of THF (6 mL) at –30 °C was transferred to the solids and the mixture was stirred at room-temperature for 20 h. The solvent was stripped and the dark residue was extracted with benzene (5 mL) and filtered through a glass filter pad. The resulting filtrate was stripped of solvent to afford **7** as a yellow-orange solid. Yield: 226 mg (81%).

Elemental analysis, Found: C, 82.55; H, 8.44%. **Calc.** for $C_{72}H_{89}P$: C, 87.75; H, 9.10%. Compound **7** is highly sensitive, and elemental analyses were consistently unsuccessful; best results are provided. Bulk purity of freshly prepared material was determined by ^1H, $^{13}C\{^1H\}$, and ^{31}P NMR spectroscopy and the composition of **7** was confirmed by HR-ESI-MS.

HR-ESI-MS (m/z) [7+H]$^+$ 985.683 (calc. 985.677).

^1H NMR (400 MHz, C_6D_6): δ = 7.96 (s, 1H), 7.84 (s, 1H), 7.61 (s, 1H), 7.60 (s, 1H), 7.40 (s, 1H), 7.34 (s, 1H), 7.06 (s, 1H), 6.43 (s, 1H), 2.95 (s, 1H), 2.68 (d, J = 13.8 Hz, 1H), 2.44 (d, J = 13.8 Hz, 1H), 2.12 (d, J = 12.8 Hz, 1H), 1.97 (d, J = 13.0 Hz, 1H), 1.80 (s, 3H), 1.75 (s, 3H), 1.73-1.40 (m, 19H), 1.39 (s, 3H), 1.38 (s, 3H), 1.33 (s, 3H), 1.32 (s, 3H), 1.30 (s, 3H), 1.27 (s, 3H), 1.23 (s, 3H), 1.19 (s, 3H), 1.12 (s, 3H), 1.12 (s, 3H), 1.11 (s,

3H), 1.11 (s, 3H), 1.06 (s, 3H), 1.04 (s, 3H), 0.97 (s, 3H), 0.95 (s, 3H), 0.84 (s, 3H), 0.79 (s, 3H) ppm.

$^{13}C\{^1H\}$ NMR (101 MHz, C_6D_6): δ = 156.9, 155.9, 153.8, 152.7, 152.7, 152.5, 149.5, 144.8, 144.0, 143.8, 143.8, 143.7, 143.4, 139.0, 139.0, 137.7, 137.1, 137.0, 134.0, 122.6, 122.4, 122.3, 119.9, 117.7, 117.3, 115.9, 111.5, 67.3, 62.8, 57.9, 54.8, 49.9, 43.7, 36.1, 35.9, 35.8, 35.8, 35.7, 35.7, 35.6, 35.3, 35.0, 35.0, 34.9, 34.8, 34.8, 34.6, 34.5, 34.4, 33.7, 33.1, 32.8, 32.6, 32.5, 32.5, 32.4, 32.3, 32.2, 32.2, 32.1, 31.8, 31.8, 31.7, 31.6, 29.5, 29.5, 29.2, 28.4, 27.1 ppm.

^{31}P NMR (162 MHz, C_6D_6): δ = –153.0 (s) ppm.

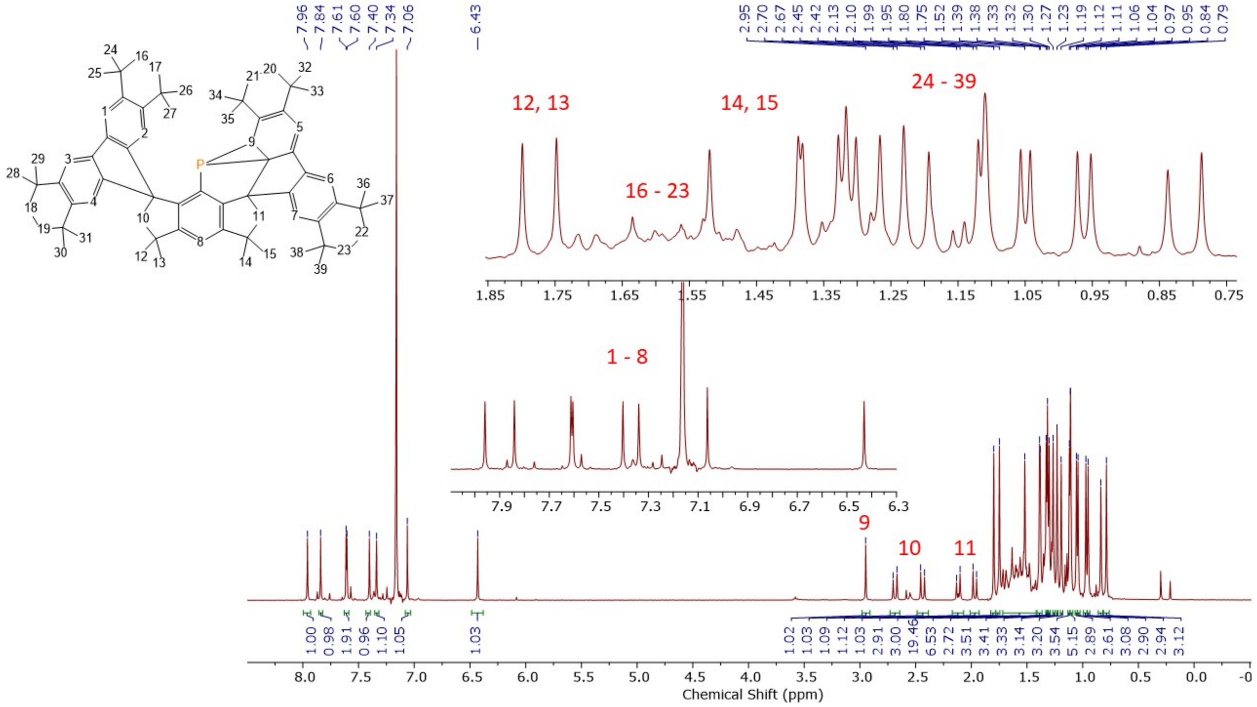

Figure S24. ^1H NMR spectrum (C_6D_6, 400 MHz) of **7** at room temperature.

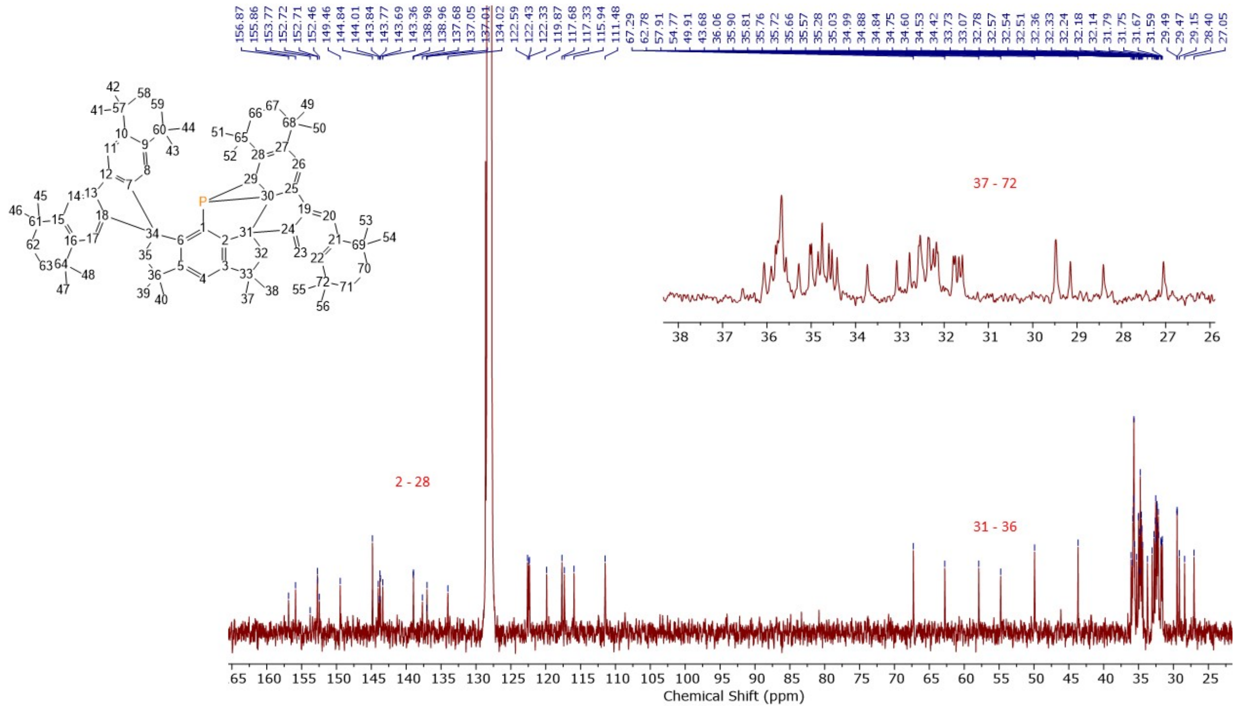

Figure S25. $^{13}C\{^1H\}$ NMR spectrum (C_6D_6, 101 MHz) **7** at room temperature. Signals arising from atoms 1, 29, and 30 were not identified.

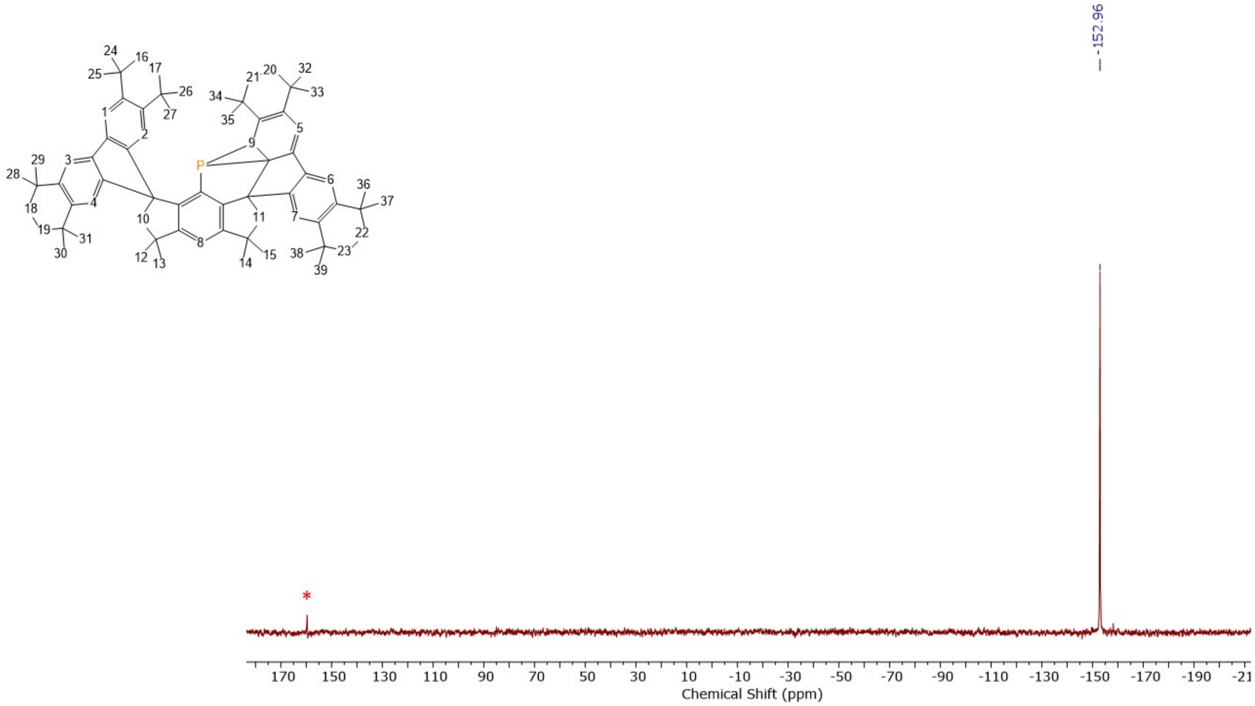

Figure S26. $^{31}P\{^1H\}$ NMR spectrum (C_6D_6, 162 MHz) of **7** at room temperature. The asterisk denotes a signal arising from unreacted precursor, **1**.

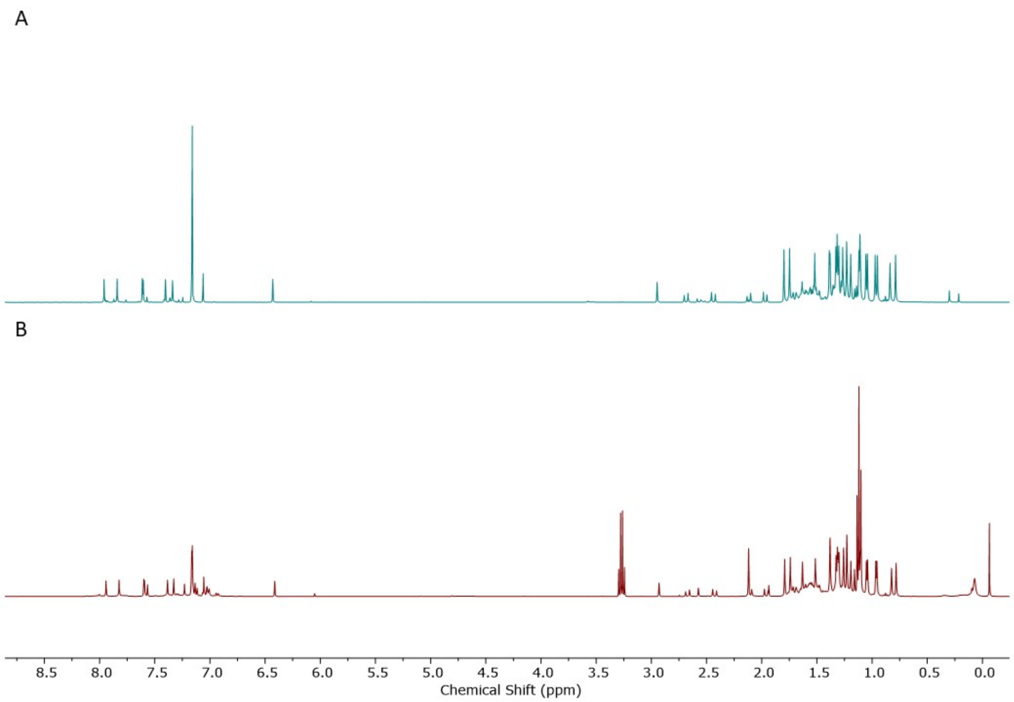

Figure S27. ^{1}H NMR spectrum (C_6D_6, 400 MHz) of (A) isolated **7** and (B) **7**, generated *in situ* from a reaction mixture between **3**•$(Et_2O)_2$, KBz, and N_2O at room temperature.

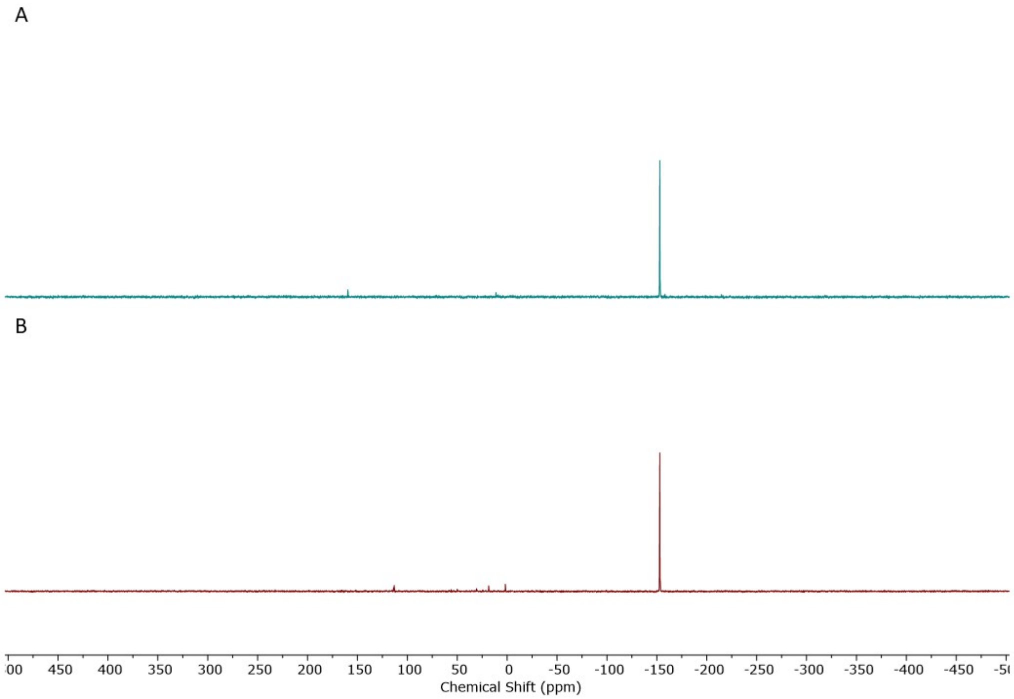

Figure S28. $^{31}P\{^{1}H\}$ NMR spectrum (C_6D_6, 162 MHz) of (A) isolated **7** and (B) a reaction mixture between **3**•$(Et_2O)_2$, KBz, and N_2O at room temperature.

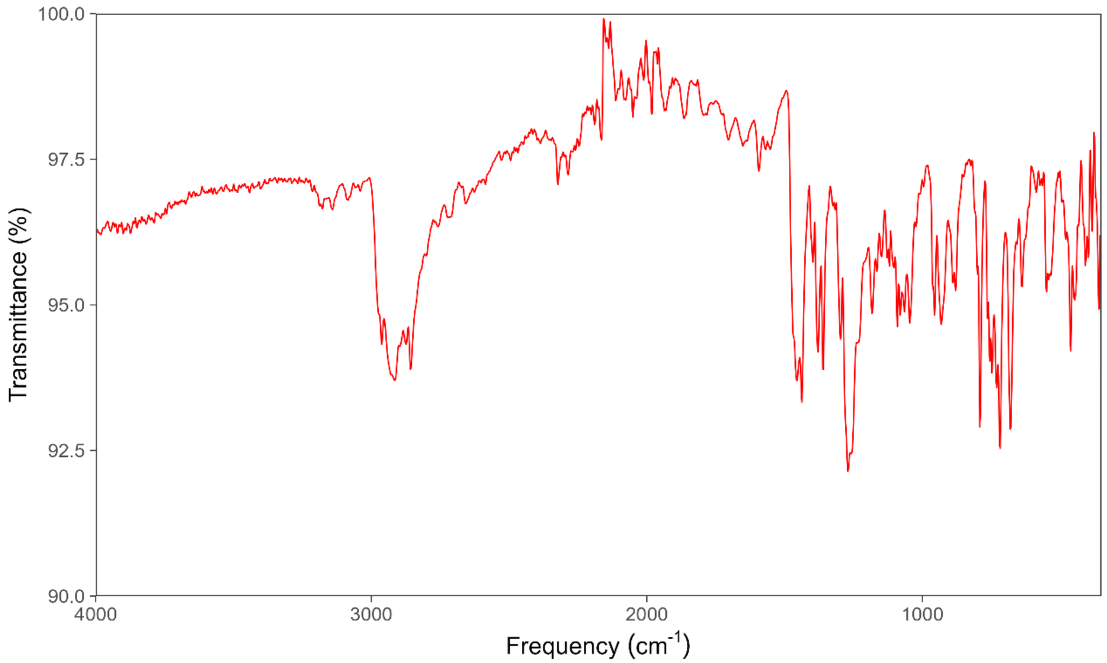

Figure S29. Experimental IR spectrum of **7**.

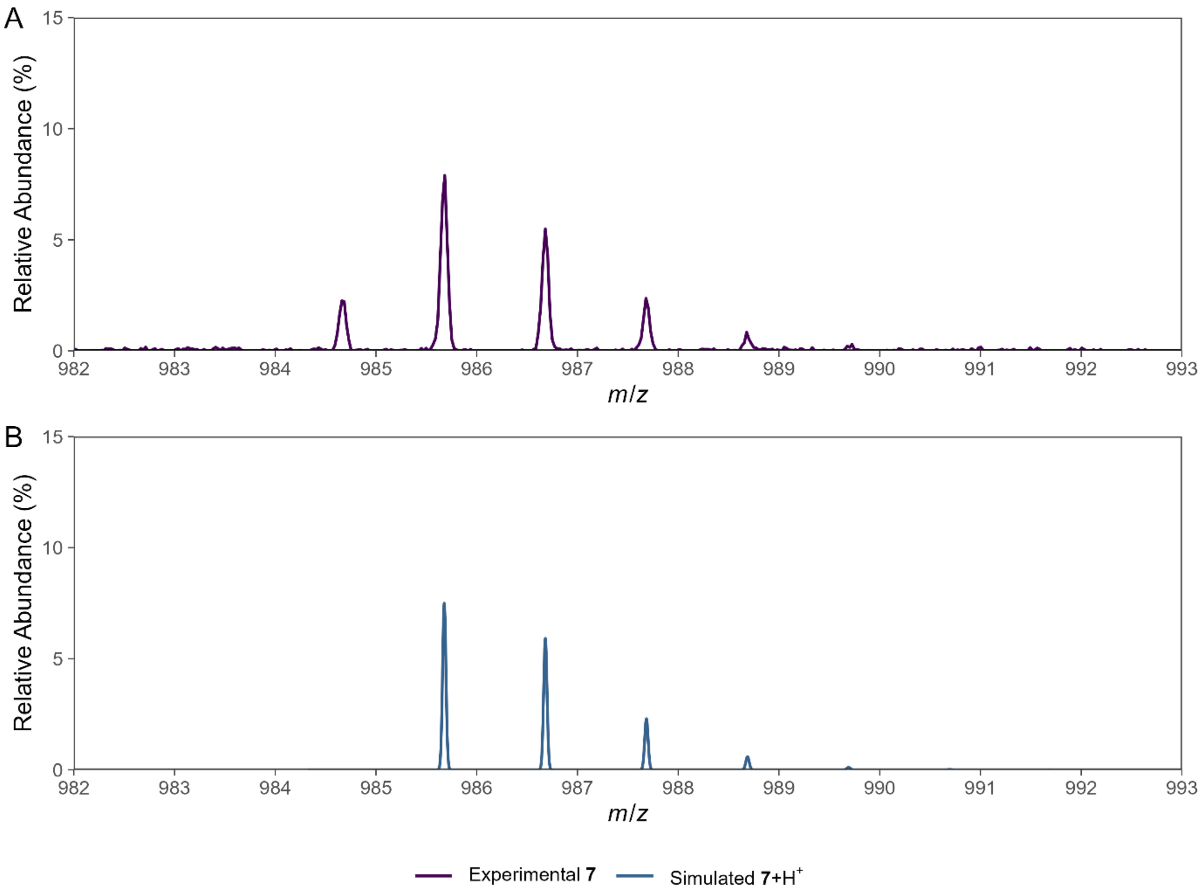

Figure S30. (A) Experimental HR-ESI-MS spectrum of **7**. (B) Simulated HR-ESI-MS spectrum for **7**+H⁺.

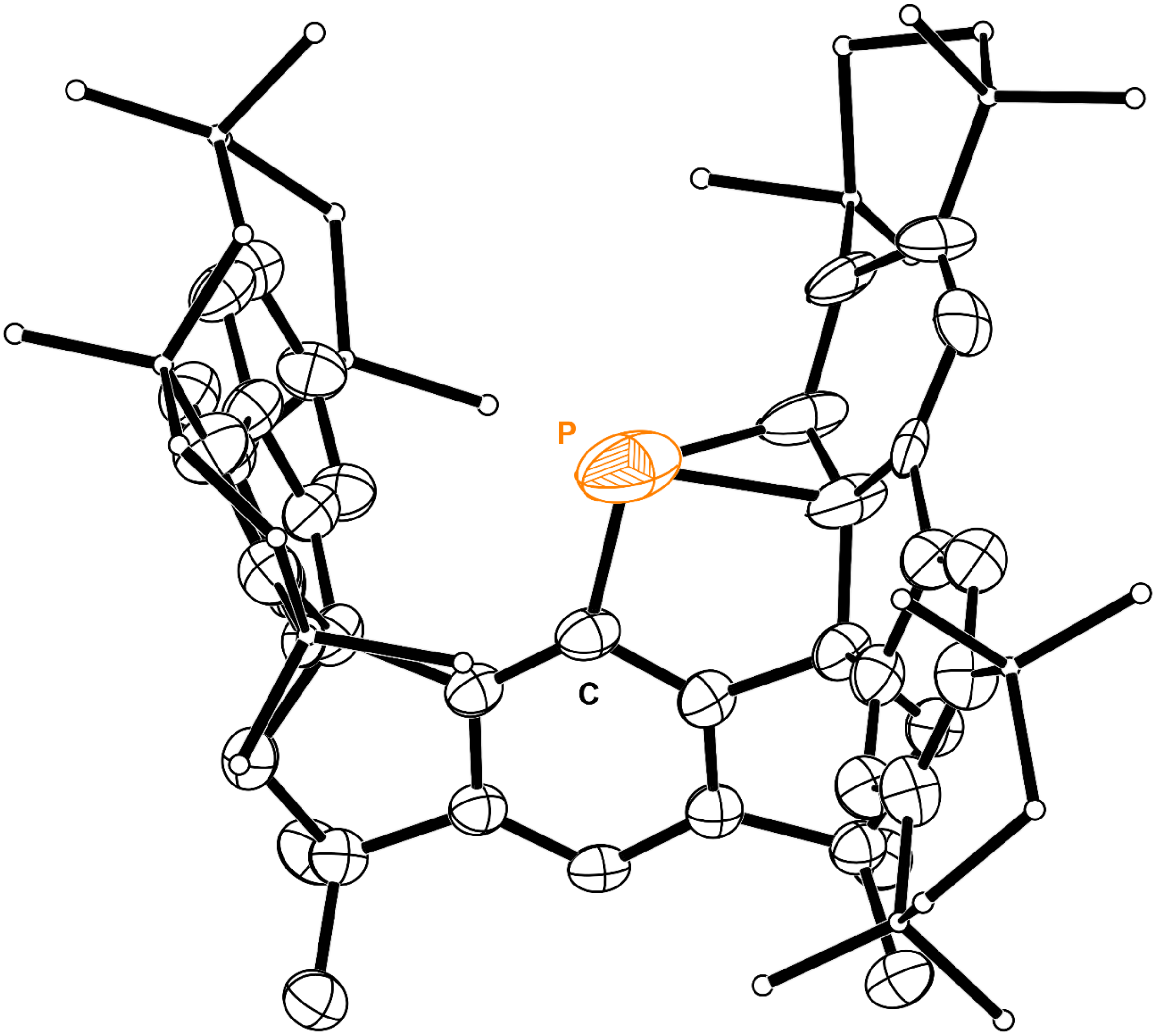

Figure S31. Thermal ellipsoid plot (50% probability) of **7**•(toluene)$_{0.5}$. Solvent molecules, H atoms, and disordered components are omitted for clarity. Color code: P orange, C black. Select alkyl C atoms are shown as spheres of arbitrary size for clarity.

2.5 Synthesis of (M^{s}FluInd*)PCO (8 and ^{13}8) *in situ*.

Method A (Synthesis of 8). A solution of **7** (25 mg, 25 μmol) in C_6D_6 (0.6 mL) was transferred to an amber-glass J-Young tube. The solution was degassed via freeze-pump-thaw before being treated with CO (1 atm) at room temperature. The sample was heated to 50 °C overnight before being analyzed by ^{1}H, ^{13}C{^{1}H}, and ^{31}P{^{1}H}, confirming the quantitative formation of **8** in solution. The solution of **8** was stripped of solvent and the resulting solid was analyzed by IR spectroscopy and HR-ESI-MS.

Method B (Synthesis of ^{13}8). A solution of **3**•$(Et_2O)_2$ (31 mg, 26 μmol) in C_6D_6 (1 mL) was transferred to a vial containing KBz (7.0 mg, 54 μmol) and stirred for 2 h. The reaction mixture was filtered through a glass filter pad and transferred to an amber-glass J-Young tube. The sample was degassed *via* freeze-pump-thaw three times before being treated with gaseous $^{13}CO_2$ (1 atm) at room temperature, and the tube was inverted three times. The sample was analyzed by ^{1}H, ^{13}C{^{1}H}, and ^{31}P NMR spectroscopy to confirm the presence of ^{13}**8**. Crystals of ^{13}**8** suitable for X-ray diffraction were grown from a concentrated mixture of hexane/toluene.

Note: 8 and ^{13}**8** were generated *in situ* and were not isolated as pure, bulk solids due to partial decomposition to form **7** during workup.

^{1}H NMR (400 MHz, C_6D_6): δ = 7.79 (s, 4H), 7.35 (s, 1H), 7.23 (s, 4H), 2.57 (s, 4H), 1.72 – 1.49 (m, 38H), 1.33 (s, 12H), 1.29 (s, 12H), 1.25 (s, 12H), 1.20 (s, 12H) ppm.

^{13}C{^{1}H} NMR (126 MHz, C_6D_6): δ = 203.0 (d, $^1J_{PC}$ = 113.4 Hz), 155.6, 153.4, 153.4, 152.1, 144.2, 143.7, 139.6, 121.8, 118.3, 117.3, 115.5, 115.2, 64.2, 58.1, 42.9, 35.7, 35.7, 34.9, 34.6, 32.9, 32.8, 32.6, 32.3, 32.3 ppm.

^{31}P NMR (8) (162 MHz, C_6D_6): δ = –232.9 (s) ppm.

^{31}P NMR (^{13}8) (162 MHz, C_6D_6): δ = –233.1 (d, $^1J_{PC}$ = 113.4 Hz) ppm.

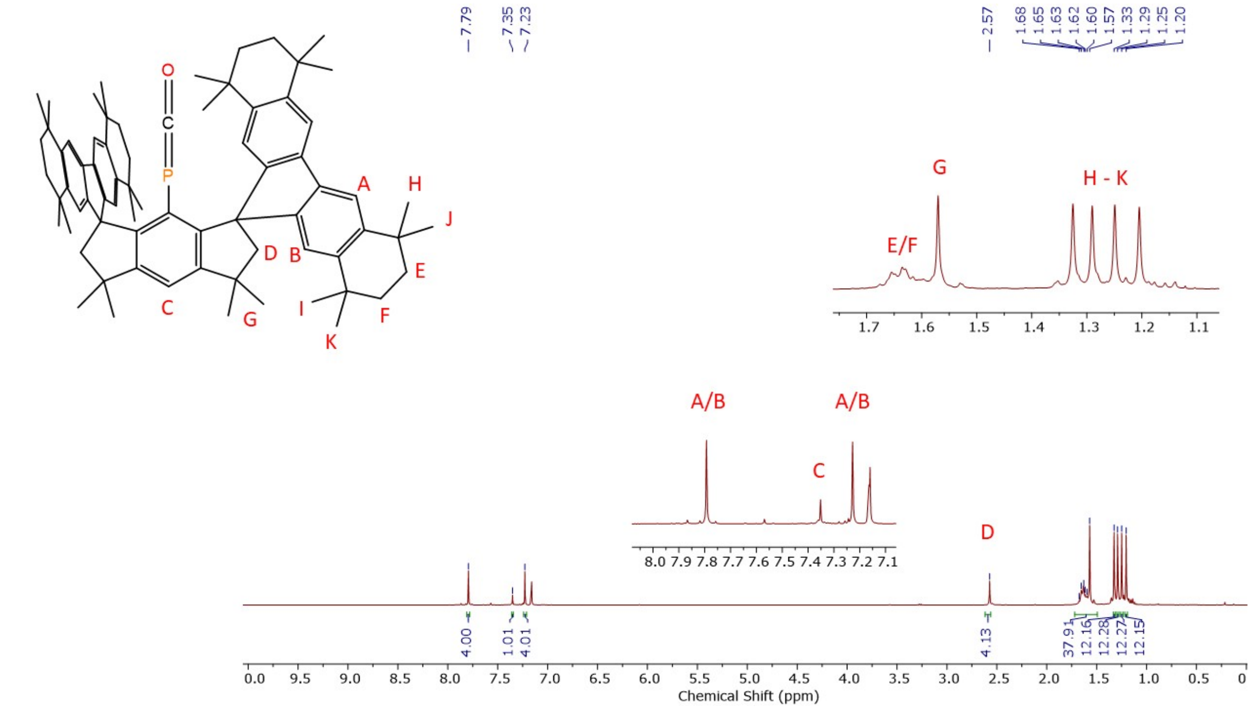

Figure S32. ^{1}H NMR spectrum (C_6D_6, 400 MHz) of **8** at room temperature, generated *in situ* from a mixture of **7** and ^{12}CO.

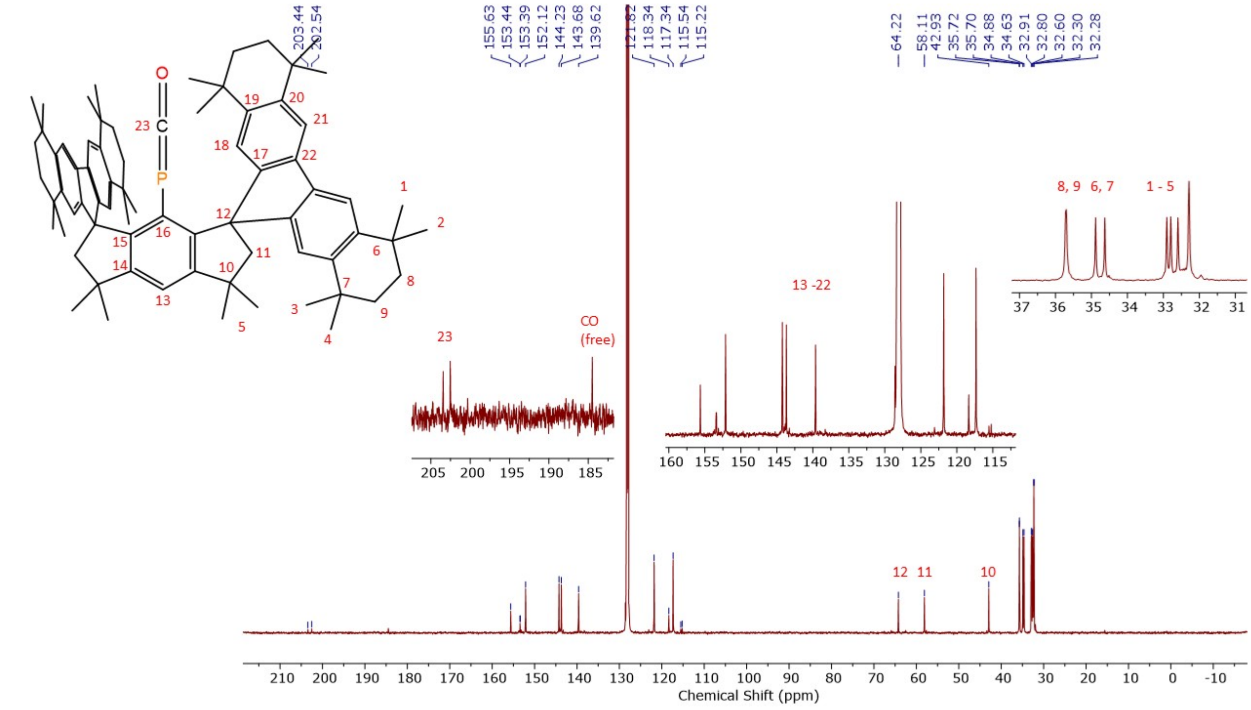

Figure S33. $^{13}C\{^{1}H\}$ NMR spectrum (C_6D_6, 126 MHz) **8** at room temperature, generated *in situ* from a mixture of **7** and ^{12}CO.

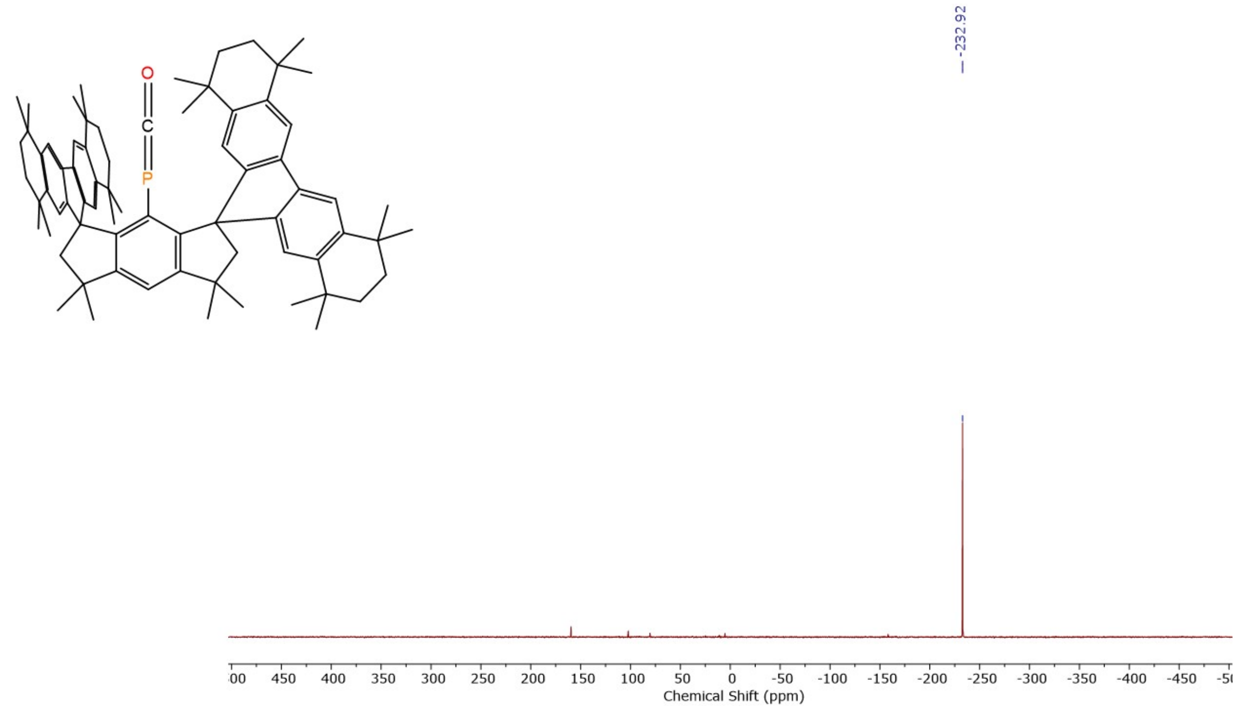

Figure S34. $^{31}P\{^{1}H\}$ NMR spectrum (C_6D_6, 162 MHz) of **8** at room temperature, generated *in situ* from a mixture of **7** and ^{12}CO.

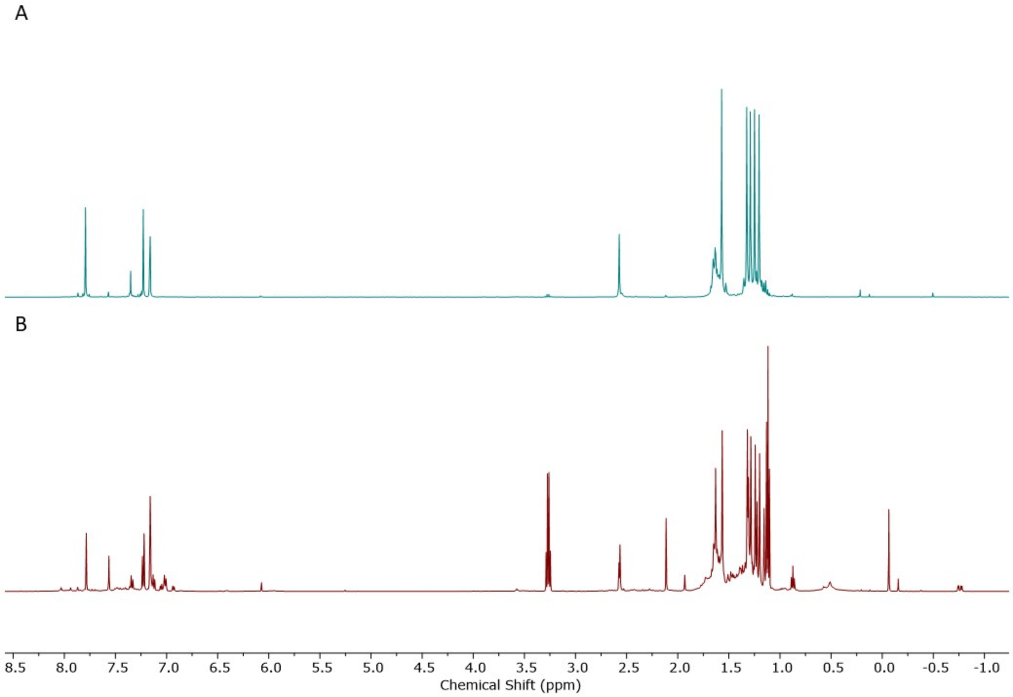

Figure S35. (A) ^{1}H NMR spectrum (C_6D_6, 400 MHz) of **8**, generated *in situ* from a mixture of **7** and ^{12}CO. (B) ^{1}H NMR spectrum (C_6D_6, 500 MHz) of ^{13}**8**, generated *in situ* from a reaction mixture between **3**•$(Et_2O)_2$, KBz, and $^{13}CO_2$ at room temperature.

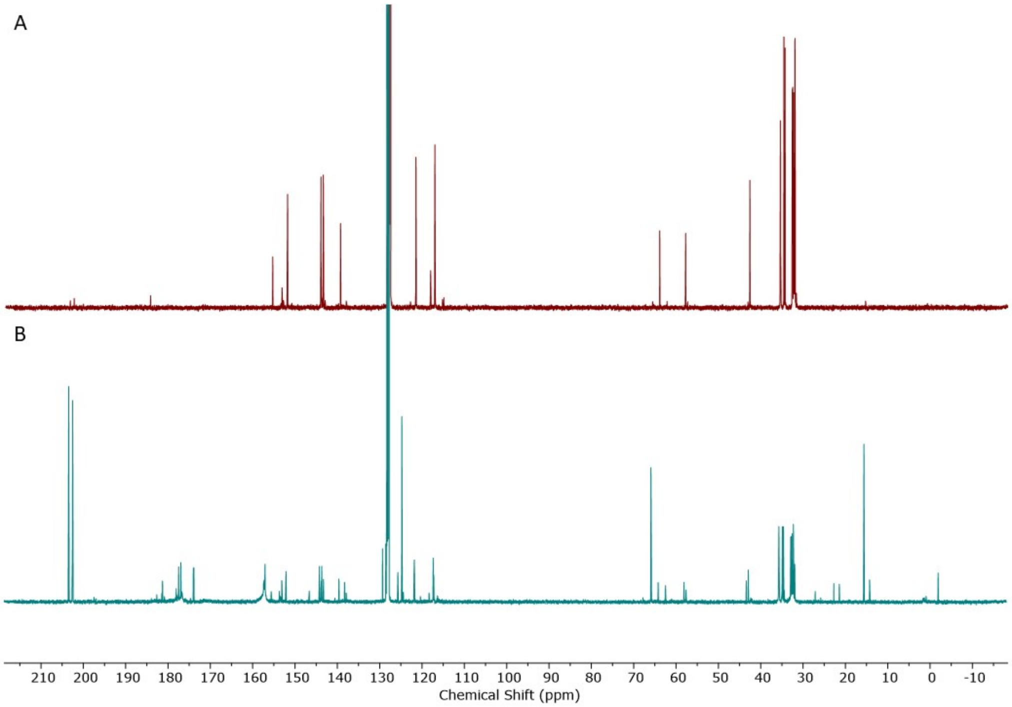

Figure S36. $^{13}C\{^{1}H\}$ NMR spectrum (C_6D_6, 126 MHz) of (A) **8**, generated *in situ* from a mixture of **7** and ^{12}CO, and (B) ^{13}**8**, generated *in situ* from a reaction mixture between **3**•$(Et_2O)_2$, KBz, and $^{13}CO_2$ at room temperature.

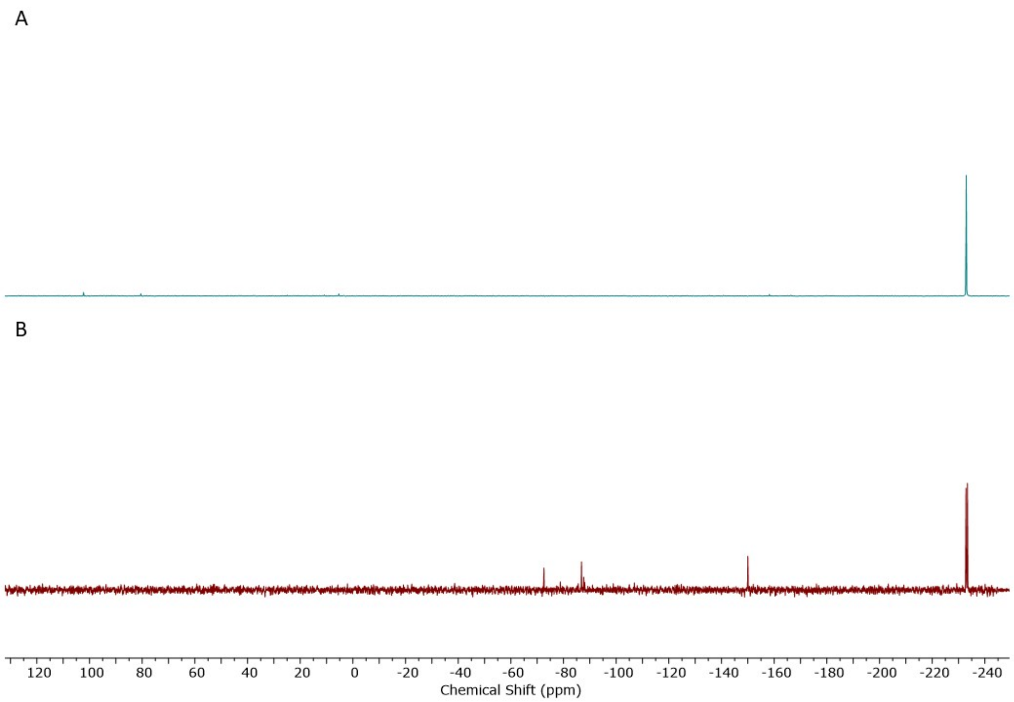

Figure S37. (A) $^{31}P\{^{1}H\}$ NMR spectrum (C_6D_6, 162 MHz) of **8**, generated *in situ* from a mixture of **7** and ^{12}CO. (B) $^{31}P\{^{1}H\}$ NMR spectrum (C_6D_6, 202 MHz) of ^{13}**8**, generated *in situ* from a reaction mixture between **3**•$(Et_2O)_2$, KBz, and $^{13}CO_2$ at room temperature.

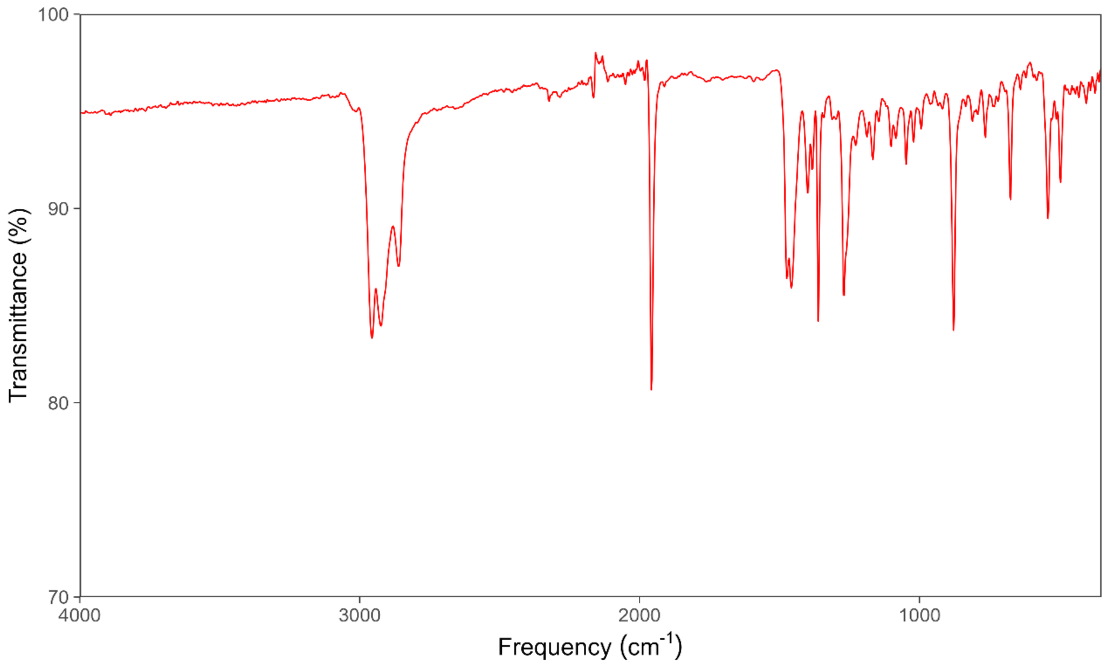

Figure S38. Experimental IR spectrum of **8**, generated *in situ* from a mixture of **7** and ^{12}CO.

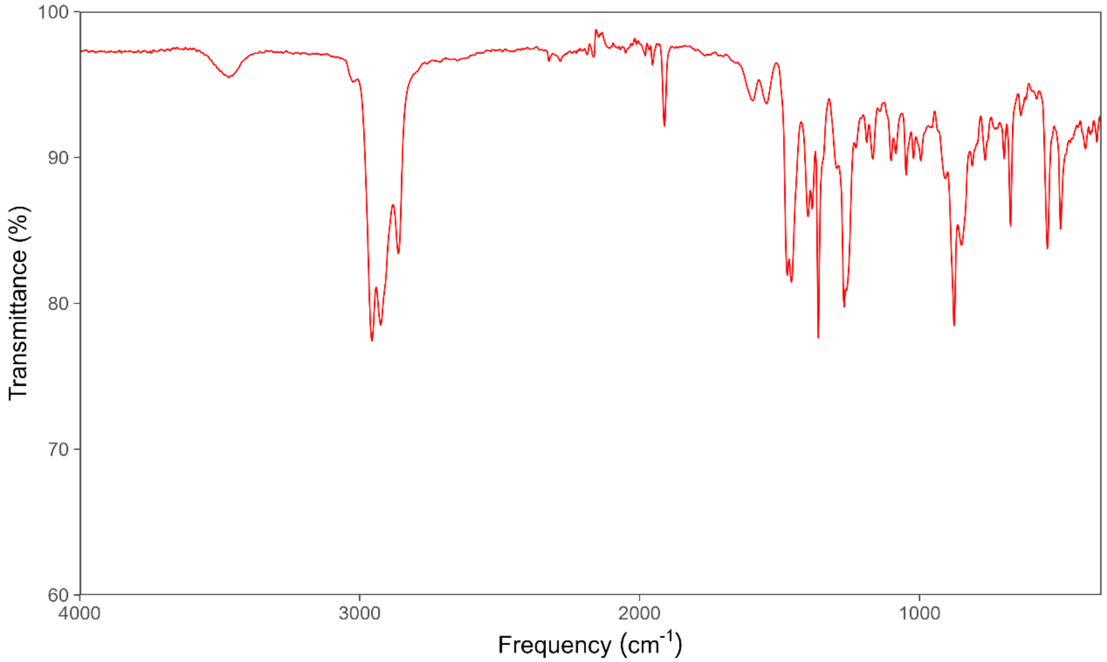

Figure S39. Experimental IR spectrum of ^{13}**8**, generated *in situ* from a reaction mixture between **3**•$(Et_2O)_2$, KBz, and $^{13}CO_2$.

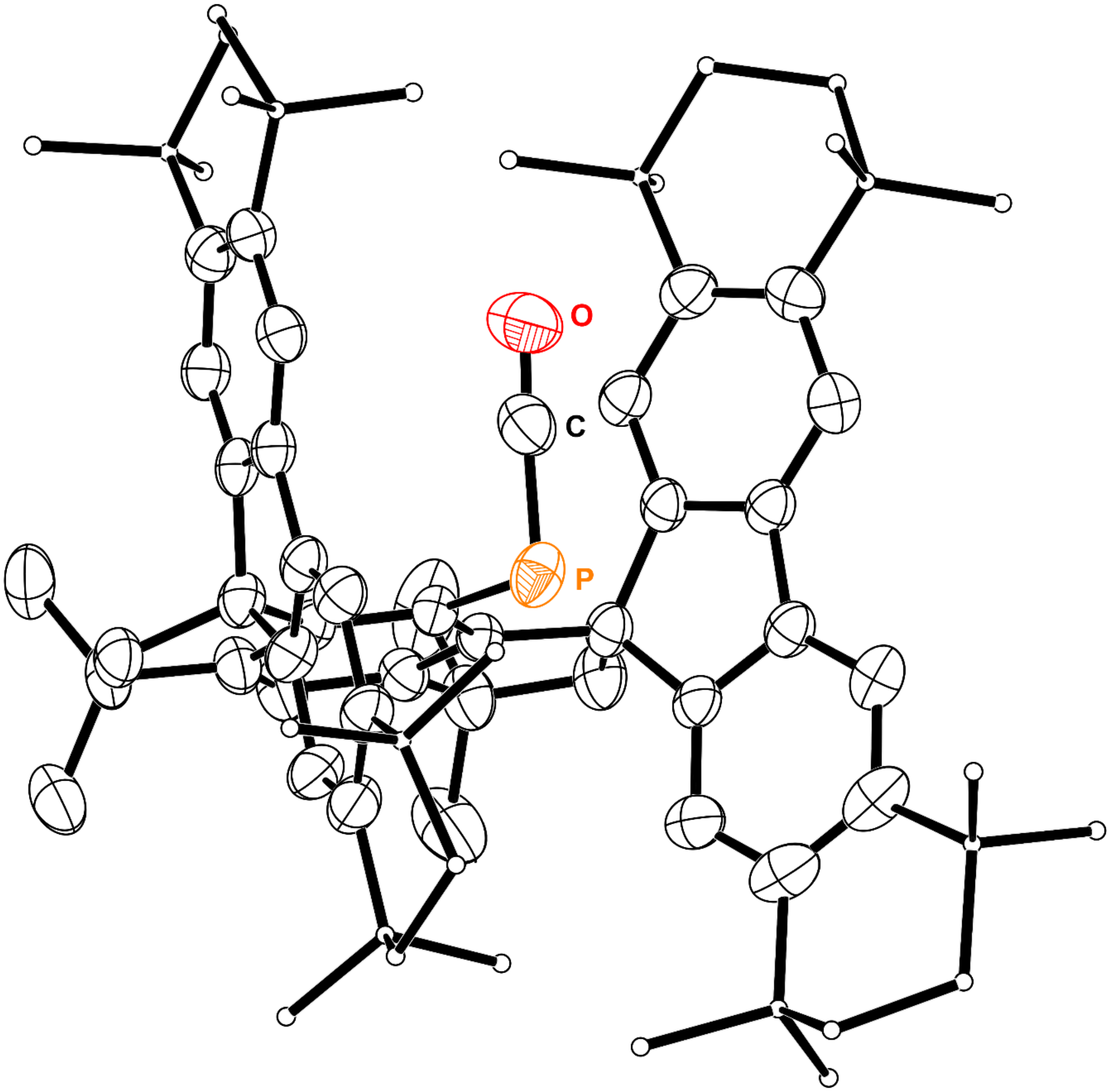

Figure S40. Thermal ellipsoid plot (50% probability) of ^{13}**8**. Solvent molecules, H atoms, and disordered components are omitted for clarity. Color code: P orange, C black, O red.

2.6 Photolysis of 8.

Procedure. A solution of **7** (18 mg, 18 µmol) in C_6D_6 (0.6 mL) was transferred to an amber-glass J-Young tube. The sample was analyzed by ^1H and $^{31}P\{^1H\}$ NMR (Supplementary Figure S41A, S42A). The solution was degassed *via* freeze-pump-thaw three times before being treated with CO (1 atm) at room temperature. The sample was heated to 50 °C overnight before being analyzed by ^1H and $^{31}P\{^1H\}$, confirming the quantitative formation of **8** in solution (Supplementary Figure S41B, S42B). The sample was then transferred to a translucent J-Young tube and irradiated with light (390 nm) for 2 h before being analyzed by ^1H and $^{31}P\{^1H\}$, confirming the near quantitative formation of **7** in solution (Supplementary Figure S41C, S42C).

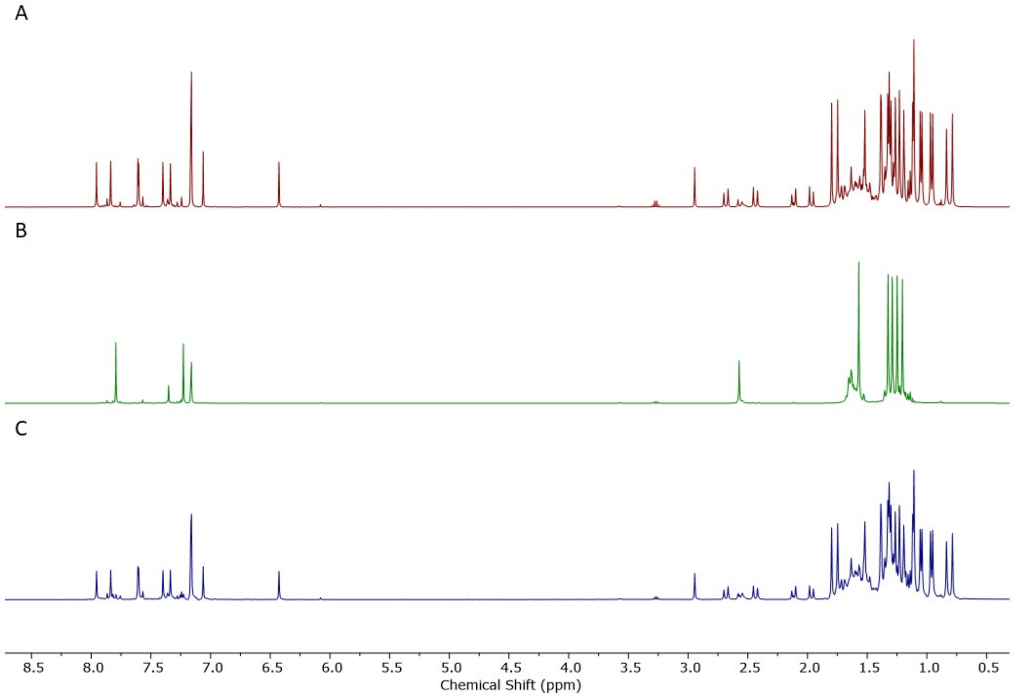

Figure S41. ^1H NMR spectrum (C_6D_6, 400 MHz) of (A) **7**, (B) **8**, generated *in situ* from a mixture of **7** and ^{12}CO, and (C) **7** generated from photolysis of **8**.

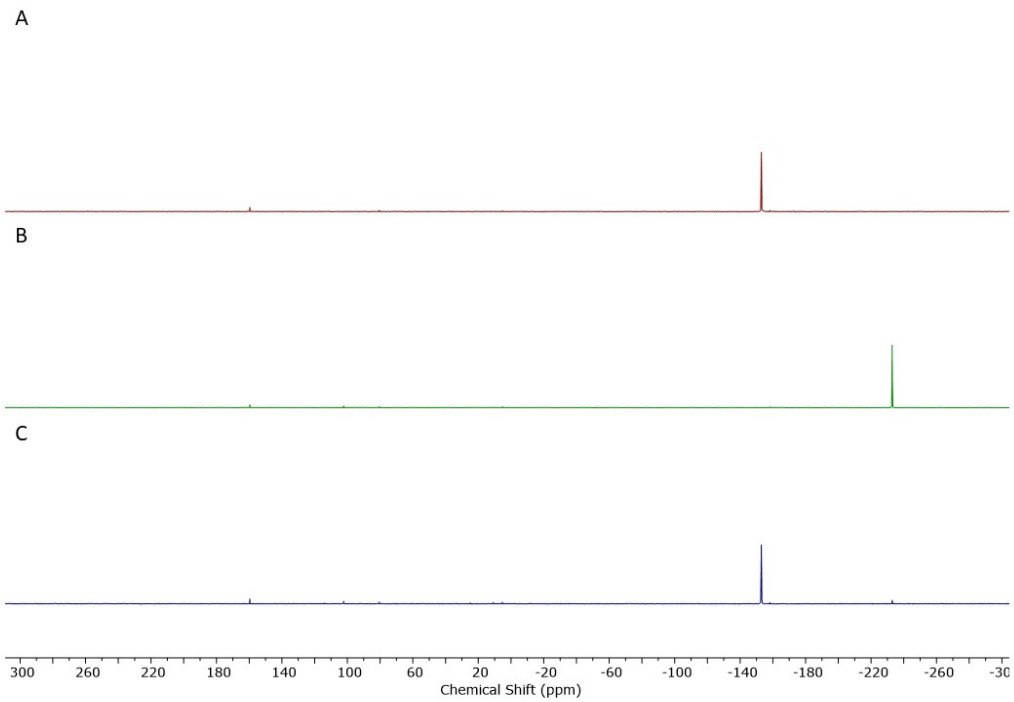

Figure S42. $^{31}P\{^{1}H\}$ NMR spectrum (C_6D_6, 162 MHz) of (A) **7**, (B) **8**, generated *in situ* from a mixture of **7** and ^{12}CO, and (C) **7** generated from photolysis of **8**.

3. Crystallographic Tables

Table S1. Crystallographic details for **4**, **5**, and ^{13}**6**.

**Compound**	**4**	**5**	^{13}**6**
**Empirical formula**	$C_{90}H_{126}KN_2O_6P$	$C_{90}H_{126}KN_2O_8P$	$C_{91}H_{126}KN_2O_8P$
**Formula Weight**	1401.99	1433.99	1446.00
**Temperature (K)**	150.00(10)	150.00(10)	149.9(3)
**Wavelength (Å)**	1.54184	1.54184	1.54184
**Crystal system**	Monoclinic	Monoclinic	Monoclinic
**Space group**	*C*2/*c*	*C*2/*c*	$P2_1/n$
a **(Å)**	27.4751(6)	27.5212(4)	14.7892(3)
b **(Å)**	22.2366(3)	22.4238(3)	21.8360(5)
c **(Å)**	29.9474(5)	30.0171(4)	27.0927(5)
**α (°)**			
**β (°)**	117.452(2)	117.540(2)	94.020(2)
**γ (°)**			
**Volume ($Å^3$)**	16236.2(6)	16425.4(5)	8727.7(3)
Z	8	8	4
**ρ_{calc} (Mg/m^3)**	1.147	1.160	1.074
**Crystal size (mm^3)**	0.188 × 0.146 × 0.141	0.2 × 0.15 × 0.11	0.26 × 0.19 × 0.06
**θ range (°)**	3.326 to 76.310	3.321 to 77.656	3.616 to 76.280
**Total reflections**	61439	61532	52306
**Unique reflections**	16656	16937	17997
**Parameters**	1111	1426	1159
**Completeness**	99.9	99.8	99.9
**R_{int}**	0.0231	0.0292	0.0340
**R_1 ($I > 2\sigma$)**	0.0787	0.0664	0.0655
**R_1 (all data)**	0.0909	0.0794	0.0826

**wR_2 (I > 2σ)**	0.2339	0.1915	0.1908
**wR_2 (all data)**	0.2511	0.2076	0.2083
**Goodness of fit, *S***	1.047	1.035	1.023
**Deposition Number (CCDC)**	2537504	2537507	2537506

Table S2. Crystallographic details for **7**•(toluene)$_{0.5}$ and ^{13}**8**.

**Compound**	**7**•(toluene)$_{0.5}$	^{13}**8**
**Empirical formula**	$C_{75.5}H_{93}P$	$C_{73}H_{89}OP$
**Formula Weight**	1031.98	1013.41
**Temperature (K)**	100.00(10)	149.97(16)
**Wavelength (Å)**	1.54184	1.54184
**Crystal system**	Monoclinic	Triclinic
**Space group**	$P2_1/n$	$P\bar{1}$
***a* (Å)**	15.2381(2)	13.7547(3)
***b* (Å)**	20.8779(2)	15.4460(3)
***c* (Å)**	21.5696(3	17.9250(4)
**α (°)**		102.078(2)
**β (°)**	97.3510(10)	91.454(2)
**γ (°)**		100.540(2)
**Volume (Å^3)**	6805.74(15)	3653.09(14)
Z	4	2
**ρ_{calc} (Mg/m^3)**	1.007	0.921
**Crystal size (mm^3)**	0.14 × 0.1 × 0.07	0.24 × 0.15 × 0.13
**θ range (°)**	2.958 to 67.078	3.983 to 67.074
**Total reflections**	115761	57966
**Unique reflections**	12036	13020
**Parameters**	1016	779

**Completeness**	99.2	99.7
**R_{int}**	0.0430	0.0587
**R_1 ($I > 2\sigma$)**	0.0775	0.0847
**R_1 (all data)**	0.0965	0.0996
**wR_2 ($I > 2\sigma$)**	0.2138	0.2353
**wR_2 (all data)**	0.2275	0.2551~~2~~
**Goodness of fit, *S***	1.018	1.021
**Deposition Number (CCDC)**	2537503	2537505

4. Computational Data

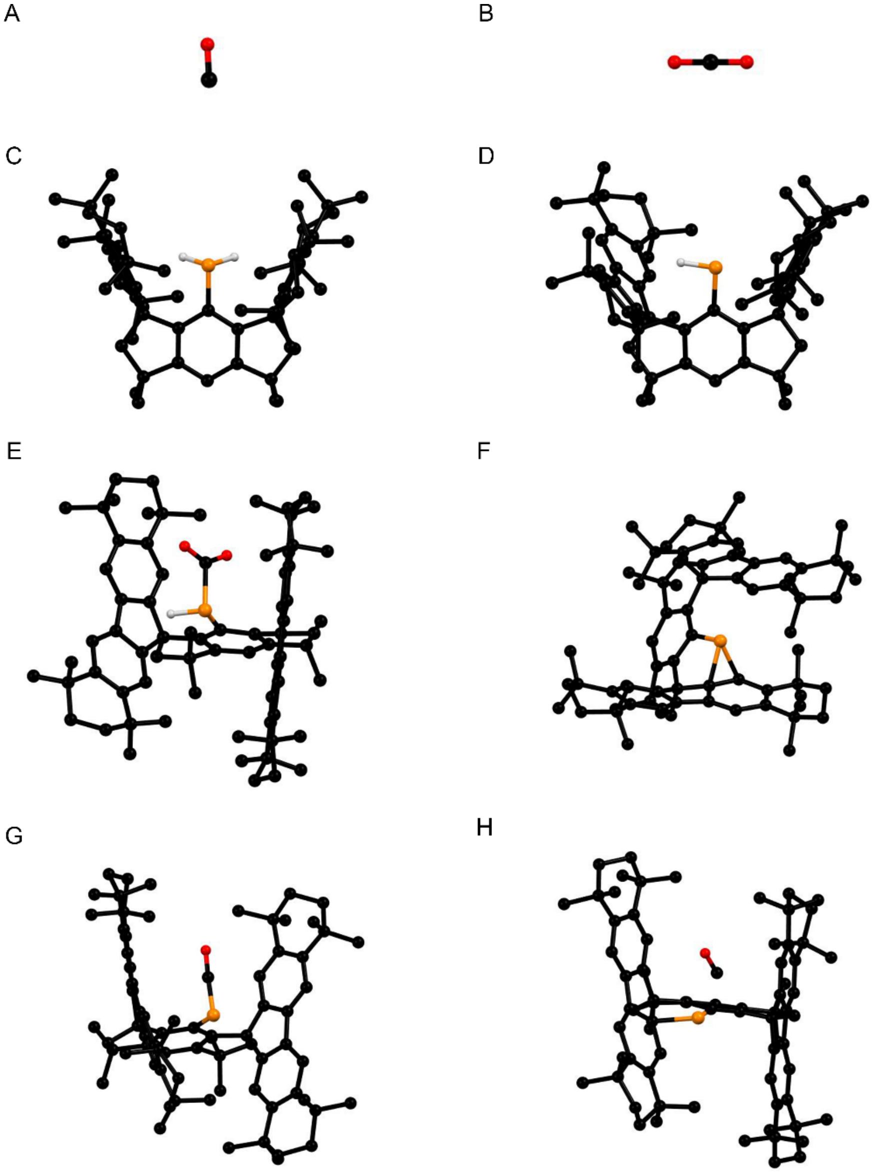

Figure S43. Ball-and-stick representation of geometry-optimized atomic coordinates (r^2SCAN-3c) of (A) CO, (B) CO_2, (C) **2**, (D) **4**–K(~~2.2.2.~~crypt)$^-$, (E) **6**–K(~~2.2.2.~~crypt)$^-$, (F) **7**, (G) **8**, and (H) **TS**. C-bound H atoms are omitted for clarity. Color code: P orange, O red, C black, H grey.

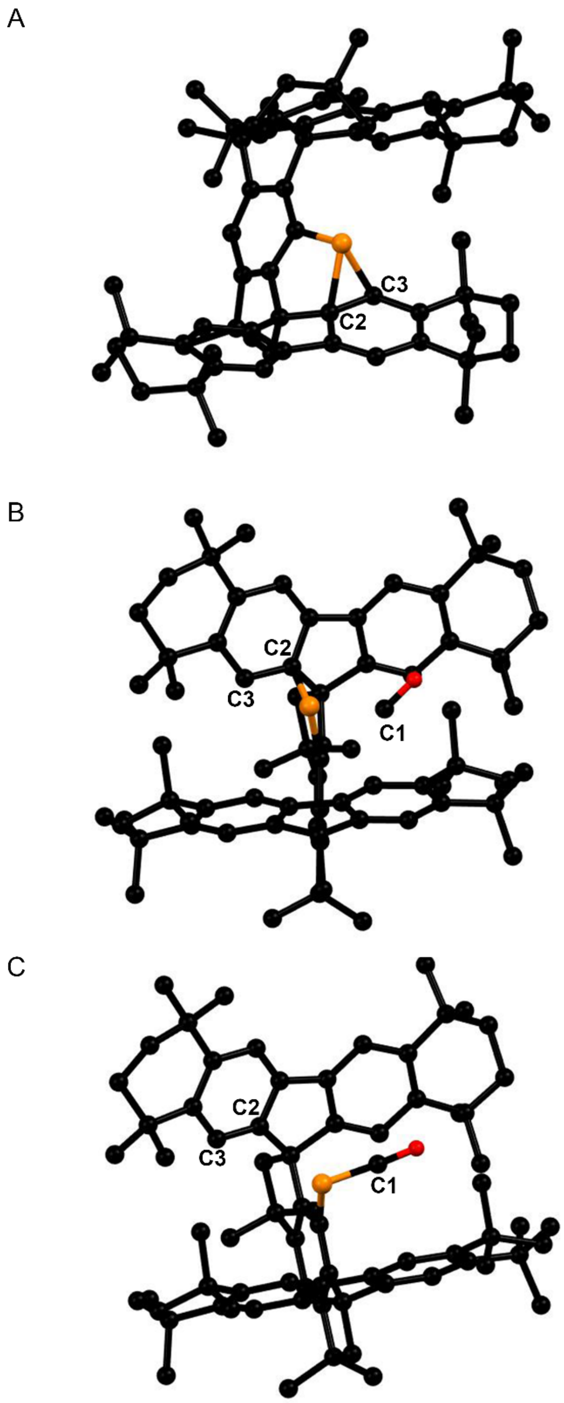

Figure S44. Ball-and-stick representation of geometry-optimized atomic coordinates (r^2SCAN-3c) of (A) **7**, (B) **TS**, and (C) **8**, in which C atoms referred to throughout as C1, C2 and C3 are labelled in each case. C-bound H atoms are omitted for clarity. Color code: P orange, O red, C black.

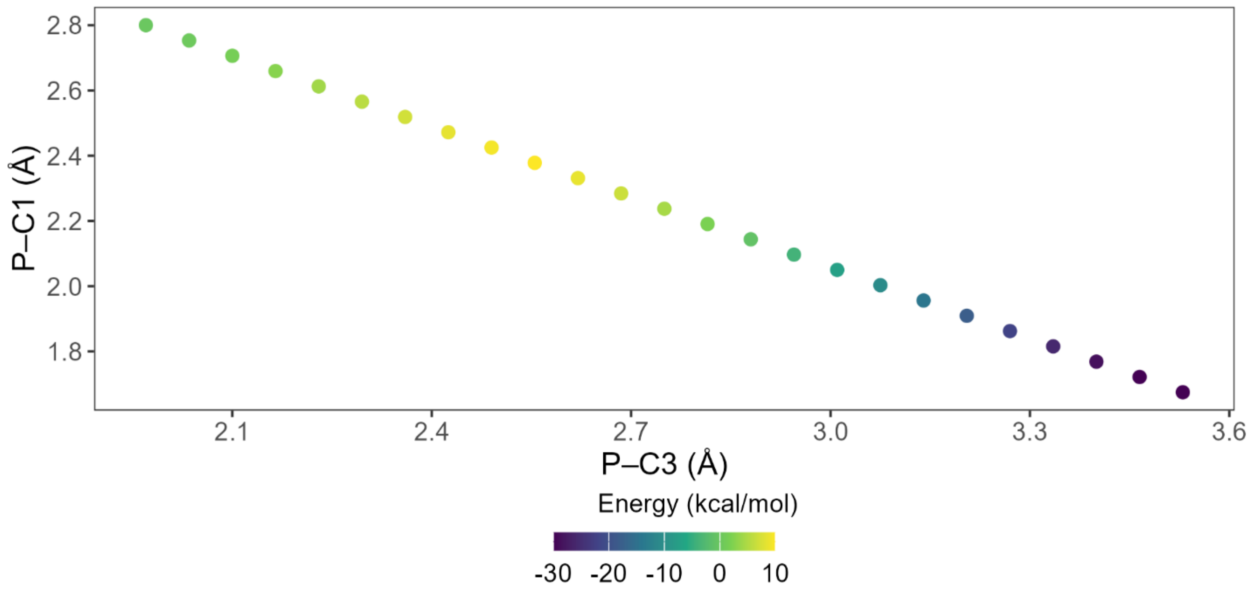

Figure S45. Simultaneous, two-dimensional relaxed surface scan (r^2SCAN-3c) along the proposed reaction coordinate for the conversion of **7**+CO to **8**, in which the P–C1 (where C1 is the carbonyl carbon) is extended from 1.675 Å to 2.8 Å, while the P–C3 distance (where C3 is the secondary fluorenyl carbon that binds P in **7**, as displayed in Figure 4) is contracted from 3.53 Å to 1.97 Å.

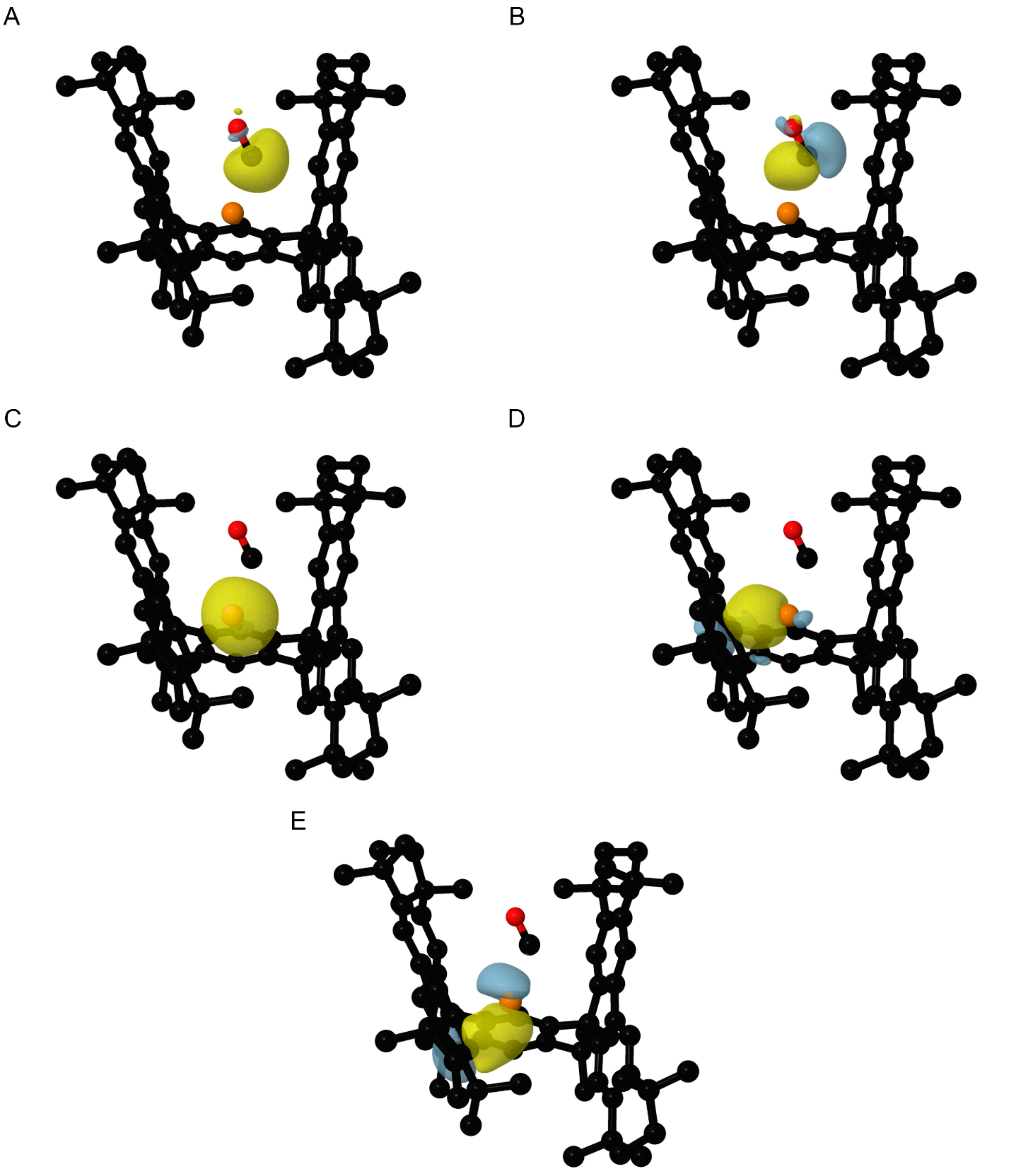

Figure S46. Surface plots (PBE0-D3BJ/def2-TZVP//r^{2}SCAN-3c) (isovalue = 0.06) depicting select NBOs identified in **TS**. (A) The C1-centered lone pair, (B) the C1-centered lone valence (or empty *p*-orbital), (C) the P-centered lone pair, (D) the P–C2 bonding orbital, and (E) the P–C3 bonding orbital. H atoms are omitted for clarity. Color code: P orange, O red, C black.

Table S3. Enthalpy of formation.[a]

Compound	ΔH_f (Eh)	ΔH_f (kcal/mol)
**4**–K(~~2.2.2.~~crypt)$^{-}$	-3137.18189576	-1968591.051
**6**–K(~~2.2.2.~~crypt)$^{-}$	-3325.76499639	-2086927.513
**2**	-3137.73347661	-1968937.17
**7**	-3136.51686703	-1968173.744
**8**	-3249.85296908	-2039292.488
**TS**	-3249.792869	-2039254.775
**CO**	-113.28531632	-71086.87585
**CO_2**	-188.5560041	-118319.4582

[a] Calculated at the r^2SCAN-3c level of theory.

Table S4. Gibbs Free Energy of formation.[a]

Compound	ΔG (Eh)	ΔG (kcal/mol)
**4**–K(~~2.2.2.~~crypt)$^{-}$	-3137.341412	-1968691.148
**6**–K(~~2.2.2.~~crypt)$^{-}$	-3325.9297715	-2087030.909
**2**	-3137.893826	-1969037.789
**7**	-3136.674194	-1968272.467
**8**	-3250.015843	-2039394.691
**TS**	-3249.955755	-2039356.986
**CO**	-113.3077484	-71100.95207
**CO_2**	-188.5749724	-118331.3609

[a] Calculated at the r^2SCAN-3c level of theory.

Table S5. Select bond lengths (Å).[a]

Bond	7	TS	8
P–C1	NA	2.226	1.675
P–C2	1.976	2.119	3.189
P–C3	1.970	2.331	3.530

[a] From theoretical coordinates (r^2SCAN-3c).

Table S6. Energies obtained for a simultaneous, two-dimensional relaxed surface scan along the proposed reaction coordinate for the conversion of **7**+CO to **8**, starting from the coordinates of **8**, in which the P–C1 (where C1 is the carbonyl carbon) is extended from 1.675 Å to 2.8 Å, while the P–C3 distance (where C3 is the secondary fluorenyl carbon that binds P in **7**, as displayed in Figure 4) is contracted from 3.53 Å to 1.97 Å.[a]

P–C3 (Å)	P–C1 (Å)	Energy (Eh)	Relative Energy (Eh)	Relative Energy (kcal/mol)
3.53	1.675	-3251.35483968	-0.05021200	-31.5082
3.465	1.721875	-3251.35364744	-0.04901976	-30.76
3.4	1.76875	-3251.35045820	-0.04583052	-28.7588
3.335	1.815625	-3251.34576595	-0.04113827	-25.8144
3.27	1.8625	-3251.33997081	-0.03534313	-22.1779
3.205	1.909375	-3251.33344181	-0.02881413	-18.081
3.14	1.95625	-3251.32750747	-0.02287979	-14.3571
3.075	2.003125	-3251.32177831	-0.01715063	-10.7621
3.01	2.05	-3251.31625662	-0.01162894	-7.29719
2.945	2.096875	-3251.31100120	-0.00637352	-3.9994
2.88	2.14375	-3251.30607557	-0.00144789	-0.90856
2.815	2.190625	-3251.30154539	0.00308229	1.934146
2.75	2.2375	-3251.29747716	0.00715052	4.486973
2.685	2.284375	-3251.29392523	0.01070245	6.715819
2.62	2.33125	-3251.29096398	0.01366370	8.574013
2.555	2.378125	-3251.28880919	0.01581849	9.92615
2.49[b]	2.425 [b]	-3251.28965930	0.01496838	9.392703
2.425	2.471875	-3251.29120938	0.01341830	8.420024
2.36	2.51875	-3251.29334626	0.01128142	7.079125
2.295	2.565625	-3251.29568925	0.00893843	5.608892
2.23	2.6125	-3251.29809073	0.00653695	4.101956
2.165	2.659375	-3251.30039242	0.00423526	2.657638
2.1	2.70625	-3251.30241504	0.00221264	1.388438
2.035	2.753125	-3251.30393426	0.00069342	0.435123
1.97	2.8	-3251.30462768	0.00000000	0

[a] Calculated at the r^2SCAN-3c level of theory. [b] Optimized structure used for input for transition state search that identified the transition state, **TS**.

Table S7. NBO analysis of **TS.** [a]

NBO	Population	% atom 1	% atom 2	%s character 1	%p character 1	%s character 2	%p character 2
P–C2 σ	1.714	43.98	56.02	9.31	90.32	7.18	92.67
P–C3 σ	1.577	47.14	52.86	0.80	98.77	2.42	97.47
LP P	1.909	N/A	N/A	74.76	25.21	N/A	N/A
LP C1	1.777	N/A	N/A	76.76	23.20	N/A	N/A
LV C1	0.647	N/A	N/A	0.62	99.26	N/A	N/A
P–C2 σ*	0.240	56.02	43.98	9.31	90.32	7.18	92.67
P–C3 σ*	0.447	52.86	47.14	0.80	98.77	2.42	97.47

[a] Calculated at the PBE0-D3BJ/def2-TZVP//r²SCAN-3c level of theory.

Table S8. Thermochemistry of select reactions.[a]

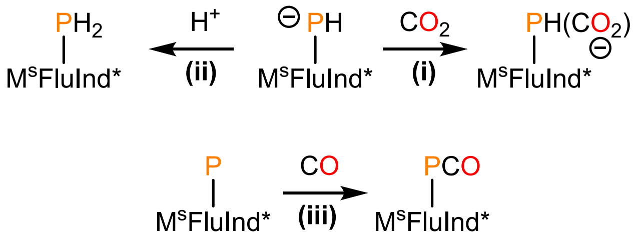

Reaction	ΔH (kcal/mol)	ΔG (kcal/mol)
**i**	–17.00	–8.40
**ii**	–346.12	–346.64
**iii**	–31.85	–21.27

[a] Calculated at the r²SCAN-3c level of theory.

Table S9. Optimized coordinates of CO (r²-SCAN-3c).

O	3.187288	5.739877	12.04109
C	2.341459	6.381006	12.43022

Table S10. Optimized coordinates of CO_2 (r^2-SCAN-3c).

O	5.959308	14.56434	17.5015
O	6.329426	16.56327	18.63348
C	6.14442	15.56387	18.06737

Table S11. Optimized coordinates of **2** (r^2-SCAN-3c).

P	5.470456	16.92317	14.1386
C	4.304873	16.42945	12.79402
C	4.208329	17.15427	11.59056
C	4.931994	18.44251	11.20556
C	4.305259	18.79931	9.810438
H	3.654116	19.6702	9.934533
H	5.087648	19.07857	9.098023
C	3.471788	17.58969	9.315584
C	3.423995	16.68996	10.53166
C	2.721317	15.4953	10.62412
H	2.124194	15.13372	9.788933
C	2.812797	14.76667	11.80187
C	2.1509	13.43155	12.07635
C	2.76797	13.02528	13.43834
H	3.546001	12.27258	13.27341
H	2.031008	12.59339	14.12274
C	3.446587	14.29212	14.06967
C	3.584092	15.22477	12.87383
C	4.146569	16.87489	8.134518
H	5.16162	16.55761	8.390636
H	4.207242	17.54304	7.26735
H	3.579411	15.98496	7.839659
C	2.063315	18.0333	8.894898
H	1.465403	17.18245	8.549398
H	2.119251	18.75955	8.075337
H	1.536954	18.49992	9.7345
C	2.51468	12.40605	10.9922
H	2.078998	12.67895	10.0244
H	2.134406	11.41377	11.26242
H	3.600976	12.33928	10.87001
C	0.620998	13.55816	12.14392
H	0.3064	14.22559	12.94989
H	0.16776	12.57633	12.3246
H	0.220677	13.94843	11.20137
C	6.447973	18.29426	11.14234
C	7.202662	17.36018	10.46924

H	6.703813	16.55395	9.938783
C	8.606088	17.43644	10.45549
C	9.368236	16.40785	9.617971
C	10.83781	16.82758	9.458643
H	11.40582	15.98196	9.048896
H	10.89967	17.63241	8.713926
C	11.44657	17.29667	10.76731
H	12.51993	17.49327	10.64541
H	11.36141	16.50182	11.52054
C	10.76854	18.56769	11.30344
C	9.245989	18.44397	11.2064
C	8.464677	19.38701	11.88969
H	8.948129	20.17551	12.46006
C	7.081775	19.32488	11.85139
C	6.056333	20.16315	12.46564
C	6.145143	21.29112	13.26392
H	7.123297	21.6954	13.50904
C	4.989995	21.91686	13.75589
C	5.163514	23.15988	14.63263
C	3.828767	23.9073	14.7786
H	3.931853	24.6633	15.56837
H	3.625888	24.45746	13.84996
C	2.672005	22.97375	15.08481
H	1.752433	23.54645	15.26402
H	2.877088	22.42171	16.01185
C	2.413437	21.96894	13.95184
C	3.727142	21.36577	13.45273
C	3.656279	20.22842	12.62915
H	2.688405	19.79956	12.37979
C	4.799187	19.63331	12.14474
C	9.279601	15.03032	10.29916
H	9.799966	14.272	9.700928
H	8.235389	14.72052	10.40995
H	9.719825	15.0466	11.29969
C	8.762529	16.29646	8.206401
H	8.69569	17.28126	7.731673
H	7.762433	15.85289	8.214153
H	9.399027	15.65641	7.583626
C	11.21004	18.74494	12.76752
H	10.90031	19.70765	13.18434
H	12.30392	18.6978	12.82987
H	10.79345	17.95173	13.39725
C	11.23181	19.79604	10.49692
H	10.96506	19.70979	9.43915

H	12.31983	19.91331	10.57222
H	10.76297	20.70889	10.87853
C	1.484538	20.87553	14.50688
H	2.006926	20.26342	15.2494
H	1.107171	20.2095	13.72629
H	0.614681	21.33634	14.99064
C	1.705741	22.66843	12.77426
H	0.743946	23.08389	13.09915
H	1.515419	21.95872	11.96254
H	2.313745	23.48019	12.36356
C	5.684102	22.72654	16.01711
H	5.824555	23.60219	16.66267
H	6.645982	22.2118	15.92424
H	4.992723	22.03752	16.51157
C	6.174435	24.14097	14.01278
H	5.902699	24.37894	12.97886
H	7.195522	23.74914	14.01524
H	6.181641	25.07313	14.59024
C	4.700195	13.86852	14.81765
C	5.879193	13.36408	14.31724
H	6.011804	13.29528	13.24001
C	6.917584	12.96745	15.17805
C	8.208934	12.44549	14.54758
C	9.336198	12.38975	15.59019
H	10.17526	11.81731	15.17298
H	9.711585	13.40709	15.7638
C	8.877974	11.7812	16.90309
H	9.724416	11.67323	17.59421
H	8.493076	10.76805	16.72599
C	7.785554	12.61853	17.58686
C	6.722793	13.04849	16.57259
C	5.520434	13.57573	17.066
H	5.372808	13.66774	18.13849
C	4.518975	13.98619	16.20286
C	3.221551	14.60927	16.44806
C	2.599043	15.00248	17.62126
H	3.083303	14.80482	18.57343
C	1.36183	15.66287	17.59394
C	0.720652	16.05171	18.92843
C	-0.39613	17.08354	18.70521
H	-0.96395	17.19781	19.63816
H	0.059966	18.06129	18.49892
C	-1.32095	16.69838	17.56476
H	-2.15334	17.41017	17.48621

H	-1.77162	15.71818	17.76988
C	-0.59222	16.64318	16.21274
C	0.751826	15.9247	16.35008
C	1.411812	15.54199	15.1684
H	0.975932	15.79422	14.20602
C	2.612793	14.87042	15.21066
C	7.953007	11.04026	13.96785
H	7.644892	10.33194	14.74311
H	8.860386	10.65593	13.48583
H	7.155745	11.07206	13.21794
C	8.674107	13.36865	13.40908
H	8.006449	13.33417	12.54421
H	9.667923	13.05669	13.0652
H	8.731749	14.40946	13.74603
C	7.170873	11.75129	18.70009
H	6.484912	12.31264	19.34121
H	7.969094	11.3583	19.34108
H	6.623138	10.90358	18.27477
C	8.40594	13.87629	18.22584
H	8.875312	14.51925	17.47531
H	9.16389	13.59272	18.96625
H	7.639769	14.47263	18.73183
C	-1.51263	15.91015	15.21972
H	-1.60639	14.85095	15.4828
H	-1.15287	15.9754	14.1885
H	-2.51223	16.36052	15.24486
C	-0.34215	18.06884	15.6882
H	-1.29237	18.59759	15.54241
H	0.184196	18.0397	14.72902
H	0.275299	18.64944	16.37972
C	0.150204	14.78376	19.59354
H	-0.59825	14.29775	18.96076
H	-0.3151	15.03225	20.55543
H	0.945987	14.05408	19.77656
C	1.748674	16.6847	19.8826
H	2.494329	15.96557	20.23332
H	1.232847	17.07452	20.76819
H	2.274715	17.51386	19.3976
H	5.140351	18.30101	14.16061
H	4.624113	16.60257	15.22942

Table S12. Optimized coordinates of **4**–K(~~2.2.2.~~crypt)⁻ (r²-SCAN-3c).

P	1.743704	10.21127	10.03847
C	3.846269	9.025045	6.757041
C	2.795911	8.015263	6.636504
C	2.674175	7.361811	7.874277
C	3.798191	7.776897	8.814349
C	4.329976	8.991044	8.074223
C	1.888159	7.748431	5.624651
H	1.979323	8.270396	4.675617
C	5.183108	10.99898	6.328014
C	1.626737	6.499852	8.093731
H	1.500118	6.078083	9.086922
C	2.617611	8.902169	10.88296
C	5.164322	9.982748	8.531566
H	5.452115	9.978725	9.580573
C	3.445457	7.89808	10.2834
C	5.556502	11.034	7.686288
C	0.552891	10.3772	12.93812
C	4.832593	5.914162	10.15361
C	4.014165	6.856586	11.01437
C	4.303172	9.999625	5.884288
H	3.955721	10.00603	4.85366
C	2.522111	8.775263	12.30868
C	3.075948	7.718377	13.01709
C	0.6909	6.223958	7.082193
C	3.823342	6.719014	12.38852
H	4.256459	5.892128	12.94675
C	0.822024	6.858318	5.830836
C	5.688945	12.0243	5.311446
C	2.745892	11.07682	13.35875
C	4.941282	6.695497	8.826615
H	4.891723	6.048028	7.94316
H	5.90098	7.223716	8.796756
C	-0.60536	9.709307	12.61622
H	-0.57423	8.62677	12.51883
C	1.919357	9.798944	13.25706
C	0.560934	11.77631	13.01141
C	2.792057	7.789164	14.50476
C	-1.78707	10.41218	12.33483
C	1.929884	12.21355	13.26955
C	-1.79636	11.81663	12.45472
C	4.104984	11.21892	13.52068
H	4.726968	10.32898	13.52154
C	4.693298	12.48974	13.6404

C	-0.60803	12.48398	12.78823
H	-0.59636	13.56946	12.84729
C	4.11861	4.565116	9.967518
H	3.929053	4.093699	10.93877
H	4.732753	3.882796	9.365006
H	3.159223	4.693991	9.459467
C	-0.47475	5.297922	7.431556
C	3.871077	13.63399	13.58402
C	6.223456	5.660757	10.7501
H	6.726234	6.609434	10.96684
H	6.845609	5.086079	10.05137
H	6.155071	5.093139	11.68537
C	2.492504	13.47501	13.37647
H	1.857328	14.35187	13.27963
C	1.86362	9.024158	14.61832
H	2.133468	9.677273	15.45628
H	0.83165	8.694019	14.78033
C	-0.15533	6.619216	4.676247
C	-3.04438	12.66176	12.18771
C	6.304519	12.20742	8.321233
C	2.067803	6.52123	14.9834
H	1.770174	6.617055	16.03609
H	2.714467	5.640887	14.89137
H	1.169823	6.343759	14.38171
C	6.199123	12.56817	13.90373
C	-2.99876	9.603329	11.86885
C	4.079957	7.954904	15.32763
H	4.600117	8.881912	15.07051
H	4.76655	7.120936	15.14297
H	3.851027	7.981544	16.40104
C	6.244177	11.31516	4.062414
H	7.001206	10.57598	4.34553
H	6.709225	12.05001	3.392576
H	5.462947	10.79813	3.497159
C	4.536325	12.94971	4.87874
H	3.729048	12.36742	4.423166
H	4.885922	13.68713	4.144182
H	4.106118	13.48211	5.731831
C	-1.43989	6.057018	8.362869
H	-1.8456	6.952881	7.883065
H	-0.91631	6.392189	9.263167
H	-2.27527	5.411879	8.665718
C	4.420936	15.06119	13.67403
C	-1.20459	4.840716	6.159593

H	-0.60495	4.06425	5.664619
H	-2.15293	4.364867	6.445954
C	0.016429	4.03441	8.161288
H	0.412082	4.255373	9.156669
H	0.804308	3.534609	7.586643
H	-0.8187	3.333523	8.287014
C	-0.53904	7.936464	3.977817
H	-0.88738	8.674898	4.707435
H	-1.34658	7.749145	3.258723
H	0.296114	8.374726	3.423569
C	5.86082	15.06371	14.21339
H	6.302319	16.05439	14.03501
H	5.835247	14.93047	15.30345
C	-1.45634	5.983625	5.192895
H	-2.0559	6.759088	5.688395
H	-2.04654	5.636778	4.332784
C	6.720607	13.9784	13.58988
H	7.758642	14.06299	13.94144
H	6.74836	14.10707	12.49895
C	6.452327	12.21841	15.38487
H	5.901999	12.88343	16.05751
H	7.522413	12.28466	15.62243
H	6.116033	11.1968	15.59117
C	-2.86866	13.41983	10.85756
H	-2.00019	14.08449	10.90527
H	-3.75721	14.02723	10.63986
H	-2.69871	12.73288	10.02348
C	7.677358	11.74056	8.839041
H	7.570293	10.90801	9.541314
H	8.18672	12.56064	9.361374
H	8.318767	11.39871	8.019011
C	6.835965	12.84577	5.921975
H	7.734954	12.21491	5.971087
H	7.074542	13.68167	5.249401
C	6.995241	11.5785	13.03662
H	6.769623	10.53481	13.27289
H	8.069364	11.7284	13.20422
H	6.790786	11.73845	11.97401
C	-3.26717	13.68906	13.31223
H	-3.30109	13.19257	14.28817
H	-4.21976	14.21157	13.15527
H	-2.47741	14.44504	13.34492
C	0.511051	5.69539	3.637945
H	1.433943	6.149716	3.261924

H	-0.16144	5.522693	2.787306
H	0.778373	4.72737	4.073064
C	5.47891	12.75847	9.499128
H	4.480186	13.05878	9.166172
H	5.986965	13.63387	9.926101
H	5.3297	12.02579	10.29619
C	6.492857	13.35297	7.311247
H	5.56923	13.94397	7.263324
H	7.273198	14.0316	7.682171
C	4.389314	15.69726	12.27019
H	4.760393	16.72998	12.30694
H	3.368814	15.71046	11.87437
H	4.99982	15.12994	11.56174
C	3.574749	15.92752	14.62419
H	3.476979	15.44587	15.60321
H	2.568795	16.11206	14.23633
H	4.058168	16.90294	14.76452
C	-4.29393	11.76979	12.12525
H	-4.56944	11.47235	13.14665
H	-5.13417	12.36157	11.73551
C	-2.5847	8.606793	10.76916
H	-3.47902	8.1756	10.30047
H	-1.99166	7.778452	11.16663
H	-1.98018	9.1014	10.00157
C	-4.07238	10.52962	11.27728
H	-3.7665	10.82433	10.26476
H	-5.00764	9.963195	11.16706
C	-3.58051	8.812965	13.05646
H	-3.94034	9.477059	13.84947
H	-2.81532	8.164128	13.49511
H	-4.41549	8.181984	12.72417
H	2.184778	9.874152	8.732155

Table S13. Optimized coordinates of **6**–K(~~2.2.2.~~crypt)$^{-}$ (r^{2}-SCAN-3c).

P	7.388078	15.13383	17.19086
O	5.529638	13.09063	16.83345
C	8.150309	13.84298	14.83747
C	8.368065	13.92089	16.22761
C	9.259328	12.9699	16.76393
C	10.17169	14.02403	18.97292
C	9.546946	11.87333	14.61504
H	9.975249	11.07708	14.00798
O	5.273664	14.35675	18.70158

C	6.187938	15.57423	14.29431
C	8.702417	12.82639	14.05739
C	9.838598	11.9761	15.96993
C	7.500074	14.89094	13.93724
C	9.605783	14.07667	20.25645
C	9.78888	16.13287	13.61246
H	10.33991	15.19754	13.59497
C	8.280057	12.8947	12.60328
C	8.576338	13.04516	20.34385
C	9.678727	12.8016	18.21717
C	8.557256	12.33198	19.1369
C	8.425263	16.09891	13.80401
C	6.314429	16.96882	14.21509
C	11.04387	15.00389	18.56117
H	11.42063	14.97974	17.54038
C	10.47317	17.35243	13.47504
C	3.826181	15.81453	14.76477
C	4.956616	15.00893	14.54874
H	4.87974	13.93152	14.6436
C	5.211608	17.77741	14.42898
H	5.322433	18.85754	14.37397
C	10.91574	16.05693	20.74296
C	11.39646	16.06174	19.41933
C	10.74681	11.04848	16.75148
C	3.959988	17.21899	14.7224
C	7.706172	17.29539	13.92637
C	9.998833	15.07011	21.13723
H	9.573433	15.09641	22.13703
C	10.85496	11.76687	18.11758
H	11.79952	12.32093	18.15986
H	10.84071	11.07138	18.96356
C	11.97101	17.31191	13.16544
C	7.593624	12.82235	21.29279
H	7.57471	13.43139	22.19266
C	7.565791	11.40622	18.90036
H	7.507589	10.92748	17.92879
C	9.74687	18.55839	13.56039
C	6.573063	11.89199	21.06224
C	6.564984	11.1645	19.8536
C	7.329311	14.11865	12.58613
H	6.291673	13.77414	12.52997
H	7.50566	14.77806	11.72912
C	8.36731	18.50808	13.81084
H	7.806815	19.43171	13.93051

C	2.497109	15.12513	15.07839
C	9.473446	13.07225	11.6525
H	9.999346	14.01262	11.83948
H	9.130817	13.08065	10.61003
H	10.19076	12.2509	11.76617
C	5.804013	13.98466	17.64833
C	12.25126	17.18891	18.83624
C	11.33534	17.11159	21.76801
C	7.520387	11.61701	12.21122
H	8.18304	10.7437	12.23031
H	7.107982	11.70879	11.1982
H	6.697477	11.43431	12.90986
C	12.12631	10.92327	16.09026
H	12.05789	10.40499	15.12654
H	12.81043	10.35241	16.73082
H	12.5604	11.91348	15.91354
C	10.13032	9.647024	16.88673
H	9.177919	9.68216	17.42187
H	10.80718	8.983493	17.43986
H	9.949318	9.207526	15.8989
C	10.40572	19.93798	13.45833
C	2.786733	18.17951	14.94557
C	5.420029	11.84394	22.06874
C	5.507799	10.10254	19.54916
C	12.61229	18.6698	13.48731
H	12.67098	18.78004	14.57916
H	13.64645	18.67385	13.11559
C	12.15104	16.97357	11.67075
H	11.63306	17.69125	11.02752
H	11.73283	15.98428	11.45686
H	13.21528	16.96399	11.40064
C	12.70918	16.24044	13.98566
H	13.78775	16.31891	13.80071
H	12.40468	15.22392	13.72015
H	12.53684	16.37709	15.05743
C	2.511757	14.64041	16.54099
H	2.710663	15.4558	17.24186
H	3.303153	13.90104	16.69824
H	1.544919	14.18514	16.79563
C	1.565722	17.43899	15.51598
H	1.712345	17.28178	16.59208
H	0.683004	18.08591	15.41275
C	3.163679	19.28541	15.94739
H	2.275367	19.88372	16.18767

H	3.923505	19.96678	15.55277
H	3.549102	18.84764	16.87405
C	1.333126	16.0993	14.84173
H	0.407346	15.63952	15.2143
H	1.19811	16.24389	13.75975
C	2.272284	13.90633	14.16626
H	2.971378	13.09525	14.38663
H	2.377248	14.17989	13.10972
H	1.25841	13.51713	14.32343
C	2.414359	18.83491	13.60078
H	2.106625	18.08478	12.86531
H	3.270564	19.37293	13.18065
H	1.59137	19.54892	13.73626
C	4.229177	10.40231	20.34532
H	3.526095	9.567201	20.2165
H	3.754528	11.29084	19.90865
C	5.128215	10.06195	18.0601
H	5.931478	9.650676	17.43928
H	4.893385	11.06372	17.68421
H	4.256119	9.407814	17.93264
C	6.082764	8.725946	19.94403
H	5.343274	7.935191	19.75929
H	6.379065	8.688826	20.99722
H	6.974927	8.508309	19.346
C	4.508926	10.63112	21.82043
H	4.974835	9.733859	22.25063
H	3.569922	10.78313	22.3707
C	5.930346	11.7488	23.51638
H	5.079059	11.63138	24.1992
H	6.477508	12.64385	23.82688
H	6.592502	10.88407	23.64061
C	4.608482	13.14583	21.89917
H	3.729072	13.13534	22.5571
H	4.294918	13.28949	20.8609
H	5.221586	14.01635	22.15379
C	12.58961	17.84669	21.27045
H	12.80365	18.68617	21.94659
H	13.44803	17.16262	21.33574
C	10.17888	18.10386	21.99334
H	10.4685	18.87662	22.7174
H	9.298413	17.58158	22.38129
H	9.87692	18.59126	21.06191
C	11.68992	16.45926	23.11615
H	12.44314	15.67518	22.9823

H	10.81931	16.01211	23.60416
H	12.096	17.21809	23.7969
C	12.43088	18.33926	19.84383
H	13.29843	18.94224	19.54273
H	11.56276	19.008	19.78738
C	11.5561	17.77464	17.59271
H	10.55596	18.14182	17.84629
H	11.43297	17.04371	16.78944
H	12.14858	18.61356	17.20423
C	13.6324	16.63962	18.43242
H	14.23293	17.42727	17.95997
H	13.53723	15.81398	17.72031
H	14.17841	16.26174	19.30393
C	11.83152	19.82882	12.8926
H	11.77602	19.71175	11.80202
H	12.35524	20.77808	13.07247
C	9.610361	20.86655	12.52309
H	8.627015	21.12233	12.92787
H	9.461833	20.39675	11.54473
H	10.1615	21.80433	12.37732
C	10.44901	20.5744	14.86141
H	10.89277	21.57747	14.81547
H	11.03207	19.96494	15.55771
H	9.440506	20.66201	15.27782
H	8.008596	14.93412	18.43891

Table S14. Optimized coordinates of **7** (r^2-SCAN-3c).

P	3.731284	16.28576	11.85159
C	3.483933	14.4935	11.49255
C	3.008523	13.79725	12.59307
C	2.685471	14.54335	13.83713
C	3.08426	13.47619	14.91204
H	4.129877	13.64811	15.19645
H	2.471193	13.54486	15.81649
C	2.967685	12.06453	14.22036
C	3.071911	12.42396	12.74064
C	3.367731	11.65235	11.61631
H	3.402791	10.56514	11.6624
C	3.654433	12.32448	10.42065
C	3.924068	11.69096	9.06739
C	4.065071	12.9267	8.128139
H	3.146117	13.04346	7.543503
H	4.893495	12.81831	7.421089

C	4.239746	14.20549	9.015986
C	3.779154	13.71719	10.37055
C	4.144711	11.17173	14.63356
H	5.09816	11.62055	14.33542
H	4.072436	10.18308	14.166
H	4.152347	11.02907	15.72082
C	1.656374	11.34876	14.57375
H	1.640045	11.08139	15.63682
H	1.554735	10.42742	13.98934
H	0.786622	11.97754	14.37331
C	2.736948	10.81781	8.63338
H	2.625572	9.949481	9.292488
H	2.88518	10.44916	7.611265
H	1.80332	11.3895	8.663574
C	5.192623	10.82491	9.085478
H	6.07423	11.4086	9.362888
H	5.371902	10.38715	8.096458
H	5.091561	10.00488	9.805275
C	1.276148	15.09934	14.04574
C	0.048598	14.52429	13.80829
H	0.002176	13.55335	13.32555
C	-1.14505	15.19409	14.13173
C	-2.46518	14.49093	13.81292
C	-3.64207	15.47315	13.92343
H	-3.66018	16.11014	13.02899
H	-4.58022	14.9027	13.91466
C	-3.55764	16.3402	15.16675
H	-3.52644	15.70259	16.0605
H	-4.45647	16.96345	15.26317
C	-2.32098	17.2517	15.16051
C	-1.07758	16.4621	14.74459
C	0.175832	17.0601	14.9345
H	0.236182	18.06615	15.34063
C	1.342779	16.40446	14.57651
C	2.721158	16.87554	14.51596
C	3.362828	17.98654	14.93429
H	2.824393	18.75149	15.48446
C	4.7856	18.17617	14.67209
C	5.403641	19.45633	15.23398
C	6.938061	19.38004	15.20219
H	7.278784	18.74712	16.03286
H	7.34501	20.38242	15.39109
C	7.465135	18.82873	13.89048
H	8.563026	18.84982	13.87659

H	7.131249	19.46491	13.06035
C	6.988246	17.39191	13.63204
C	5.506567	17.25646	13.95822
C	4.858623	16.03117	13.4469
H	5.476143	15.13837	13.34922
C	3.440511	15.86131	13.75909
C	-2.46044	13.93283	12.37829
H	-2.20154	14.71572	11.65754
H	-3.45856	13.55224	12.13041
H	-1.75519	13.10627	12.25027
C	-2.6587	13.32004	14.79648
H	-1.83118	12.60679	14.71878
H	-3.59077	12.78554	14.57555
H	-2.69507	13.66559	15.83418
C	-2.52838	18.42132	14.17839
H	-2.66444	18.06927	13.15125
H	-1.65986	19.08795	14.1804
H	-3.40965	19.00863	14.46431
C	-2.15939	17.8252	16.57961
H	-3.10282	18.28298	16.90042
H	-1.38705	18.59786	16.63299
H	-1.90034	17.03539	17.29279
C	7.24741	17.07012	12.15132
H	6.666321	17.72474	11.49365
H	6.996184	16.03934	11.88804
H	8.31098	17.22215	11.93368
C	7.795104	16.40176	14.49833
H	8.860749	16.46626	14.24661
H	7.476167	15.36846	14.32521
H	7.676646	16.61071	15.56589
C	4.910189	20.65351	14.39827
H	5.23241	20.57193	13.35584
H	5.297882	21.59221	14.81259
H	3.816395	20.70745	14.39873
C	4.990709	19.67326	16.70246
H	3.932984	19.92766	16.81401
H	5.570707	20.5038	17.1218
H	5.192442	18.7783	17.301
C	5.653255	14.78118	8.984294
C	6.840163	14.22187	9.401793
H	6.828899	13.25701	9.899273
C	8.066247	14.88358	9.207026
C	9.340145	14.18589	9.687057
C	10.52231	15.16689	9.673263

H	10.4289	15.84699	10.53147
H	11.45329	14.6043	9.823383
C	10.58752	15.97272	8.388269
H	10.65906	15.29309	7.528787
H	11.49428	16.59177	8.366936
C	9.363036	16.88521	8.206451
C	8.075145	16.13298	8.555544
C	6.85264	16.72463	8.202289
H	6.84484	17.70952	7.742833
C	5.654174	16.06506	8.415014
C	4.270222	16.46239	8.164299
C	3.711062	17.65331	7.730856
H	4.359112	18.47841	7.448282
C	2.318467	17.81819	7.686134
C	1.768867	19.16645	7.214991
C	0.26026	19.06294	6.93969
H	-0.14335	20.07448	6.799371
H	0.106584	18.53391	5.989314
C	-0.48081	18.34272	8.051553
H	-1.56411	18.37027	7.87404
H	-0.31086	18.86092	9.004504
C	-0.03913	16.87749	8.193252
C	1.484225	16.76409	8.113723
C	2.068785	15.54766	8.502313
H	1.440437	14.7238	8.832834
C	3.436658	15.39807	8.527815
C	9.184687	13.66757	11.12822
H	8.897119	14.47761	11.80677
H	10.14105	13.25613	11.47308
H	8.438988	12.87125	11.21113
C	9.632816	12.98435	8.766297
H	8.80389	12.26896	8.790536
H	10.54201	12.46491	9.09304
H	9.764654	13.29453	7.725303
C	9.502668	18.13119	9.100474
H	9.617649	17.86041	10.15369
H	8.618317	18.77204	9.023161
H	10.37865	18.71826	8.798495
C	9.338318	17.33885	6.734971
H	10.31703	17.75101	6.460939
H	8.591297	18.11631	6.549815
H	9.122158	16.4942	6.072239
C	-0.55118	16.36598	9.55381
H	-0.0253	16.85542	10.38098

H	-0.4253	15.28541	9.669011
H	-1.62254	16.58378	9.645949
C	-0.6706	16.02491	7.075639
H	-1.76503	16.06415	7.138908
H	-0.36113	14.97832	7.164516
H	-0.3681	16.37285	6.082941
C	2.03878	20.22774	8.299528
H	1.574124	19.95864	9.252828
H	1.64935	21.20363	7.984042
H	3.113434	20.33102	8.481172
C	2.448635	19.61139	5.90817
H	3.507651	19.84777	6.046475
H	1.958377	20.51553	5.527514
H	2.371111	18.83035	5.144295

Table S15. Optimized coordinates of **8** (r^2-SCAN-3c).

P	3.120048	7.389568	11.92835
O	3.908088	4.741128	11.29164
C	1.50196	6.922181	12.74377
C	0.325767	6.807346	11.99387
C	0.174243	6.839087	10.48443
C	-1.3787	6.956455	10.30381
H	-1.61508	8.007237	10.10487
H	-1.72284	6.371807	9.444996
C	-2.05576	6.524234	11.63101
C	-0.9073	6.593214	12.61803
C	-0.99409	6.479671	13.99925
H	-1.95539	6.314738	14.48309
C	0.167705	6.602777	14.75325
C	0.262489	6.554756	16.26415
C	1.788318	6.690762	16.52172
H	2.189152	5.736264	16.87702
H	2.0103	7.440605	17.28736
C	2.494826	7.056584	15.17145
C	1.401993	6.825095	14.13891
C	-0.26576	5.217841	16.80446
H	-1.33878	5.107965	16.61069
H	-0.11153	5.154402	17.88806
H	0.253252	4.376982	16.33203
C	-0.53123	7.703973	16.90438
H	-0.16748	8.677511	16.56291
H	-0.43627	7.669566	17.99588

H	-1.59575	7.633205	16.65435
C	-2.6388	5.104557	11.55065
H	-3.47411	5.077297	10.84124
H	-3.01288	4.782622	12.52916
H	-1.89058	4.38144	11.21715
C	-3.1776	7.500423	12.0126
H	-2.78452	8.513912	12.14562
H	-3.66633	7.19765	12.94546
H	-3.94282	7.525957	11.22805
C	0.878036	7.95567	9.732988
C	0.875216	9.308117	9.993327
H	0.350046	9.6788	10.87073
C	1.586709	10.19974	9.17333
C	2.280885	9.69816	8.051322
C	2.25847	8.319924	7.793196
H	2.796256	7.924425	6.936378
C	1.582419	7.450572	8.632916
C	1.521224	5.992906	8.676792
C	2.135483	5.02328	7.902208
H	2.726596	5.324674	7.041706
C	2.032863	3.663936	8.233768
C	2.76444	2.652099	7.349504
C	2.303304	1.221995	7.671199
H	1.313724	1.056483	7.224073
H	2.984722	0.512928	7.182651
C	2.239127	0.955277	9.164344
H	3.225253	1.127737	9.615824
H	1.99065	-0.09691	9.356511
C	1.206424	1.844803	9.874203
C	1.326702	3.292339	9.395379
C	0.691114	4.287709	10.15705
H	0.174487	4.011586	11.07115
C	0.767506	5.613878	9.798236
C	-0.22046	1.338368	9.586018
H	-0.45491	1.371067	8.517595
H	-0.96104	1.959039	10.1019
H	-0.3377	0.305544	9.93688
C	1.470052	1.740268	11.3872
H	1.533384	0.684436	11.6771
H	0.675006	2.194947	11.98619
H	2.414371	2.227535	11.64771
C	2.476412	2.906942	5.859648
H	1.39805	2.928443	5.669134
H	2.916429	2.102759	5.257734

H	2.903322	3.849793	5.505785
C	4.281002	2.787249	7.589553
H	4.623846	3.796051	7.33769
H	4.831358	2.072774	6.96505
H	4.542259	2.610317	8.637163
C	3.793681	6.28302	15.01045
C	3.982422	4.921295	14.94338
H	3.117148	4.262177	14.95668
C	5.273839	4.374812	14.84371
C	5.405323	2.850718	14.80745
C	6.817371	2.43572	14.36565
H	6.947873	1.36418	14.56698
H	6.899414	2.557628	13.27753
C	7.902917	3.242152	15.05411
H	8.896078	2.867242	14.77306
H	7.822741	3.120303	16.14281
C	7.826269	4.738665	14.71391
C	6.386123	5.242944	14.83552
C	6.174775	6.628776	14.89936
H	7.025066	7.304393	14.878
C	4.895437	7.148847	14.9924
C	4.404567	8.523569	15.04912
C	5.057663	9.744861	15.00096
H	6.140761	9.768608	14.9213
C	4.339943	10.94975	15.04252
C	5.127538	12.25955	14.94885
C	4.253261	13.45398	15.36456
H	4.175426	13.47704	16.45955
H	4.763884	14.38123	15.07275
C	2.86322	13.40212	14.75513
H	2.297283	14.30664	15.01497
H	2.938719	13.38625	13.65957
C	2.06892	12.17022	15.21373
C	2.930479	10.9111	15.10416
C	2.284819	9.664402	15.13563
H	1.200068	9.62021	15.15759
C	3.005502	8.491102	15.12358
C	5.109054	2.299818	16.21657
H	5.807085	2.696978	16.95992
H	5.180627	1.205154	16.22139
H	4.098858	2.577485	16.53573
C	4.412233	2.224614	13.81402
H	3.37079	2.335887	14.1305
H	4.611382	1.149707	13.72451

H	4.517838	2.680483	12.82514
C	8.767534	5.478367	15.68067
H	8.385712	5.437035	16.70637
H	8.907416	6.528956	15.41038
H	9.756087	5.004188	15.66246
C	8.311737	4.983201	13.2714
H	9.343087	4.629646	13.14872
H	8.284922	6.051816	13.03375
H	7.678491	4.472096	12.53996
C	1.633631	12.3307	16.68513
H	1.020918	13.23246	16.80753
H	2.494255	12.40011	17.3569
H	1.040183	11.46668	17.00328
C	0.803041	12.0829	14.34423
H	0.097199	11.32547	14.69809
H	1.057628	11.8558	13.30457
H	0.280963	13.04689	14.3641
C	6.358641	12.24556	15.8718
H	7.119257	11.53006	15.54658
H	6.073433	11.99759	16.89977
H	6.824977	13.23796	15.87394
C	5.603775	12.43752	13.49315
H	6.180854	13.36481	13.39024
H	4.760865	12.47104	12.79628
H	6.242003	11.60064	13.19103
C	3.526798	5.80357	11.57542
C	3.023224	10.60286	7.063044
C	1.654258	11.66773	9.592888
C	3.287989	11.98735	7.676009
H	4.137168	11.9141	8.368027
H	3.601817	12.67162	6.876526
C	2.081333	12.54731	8.408286
H	1.235097	12.64456	7.714758
H	2.296241	13.55904	8.77789
C	4.386405	10.00968	6.664581
H	4.290538	9.104973	6.057489
H	4.943381	10.74247	6.068626
H	4.979652	9.765173	7.552066
C	2.15859	10.74486	5.794291
H	1.971924	9.764257	5.344355
H	1.185248	11.19163	6.018998
H	2.667736	11.373	5.052983
C	0.29451	12.1861	10.09062
H	-0.48814	12.01115	9.344405

H	-0.01672	11.71727	11.02838
H	0.359858	13.26502	10.27482
C	2.678189	11.78864	10.74041
H	2.3945	11.14832	11.58257
H	3.677561	11.47288	10.42676
H	2.733298	12.8263	11.09477

5. References.

1. D. Wang, C. Zhai, Y. Chen, Y. He, X.-d. Chen, S. Wang, L. Zhao, G. Frenking, X. Wang and G. Tan, *Nat. Chem.*, 2022, **15**(*2*), 200–205.
2. P. J. Bailey, R. A. Coxall, C. M. Dick, S. Fabre, L. C. Henderson, C. Herber, S. T. Liddle, D. Loroño-González, A. Parkin and S. Parsons, *Chem.–Eur. J.*, 2003, **9**(*19*), 4820–4828.
3. J. S. Wenger, N. Gaschik, W. J. Rowe, A. E. Crumpton, B. van IJzendoorn and M. Mehta, *Chem. Sci.*, 2026, DOI: 10.1039/d6sc00723f.
4. Rigaku Oxford Diffraction, *CrysAlis[Pro]*, 2020.
5. O. V. Dolomanov, L. J. Bourhis, R. J. Gildea, J. A. K. Howard and H. Puschmann, *J. Appl. Crystallogr.*, 2009, **42**(*2*), 339–341.
6. (a) G. M. Sheldrick, *Acta Crystallogr. Sect. A*, 2015, **71**(*1*), 3–8; (b) G. M. Sheldrick, *Acta Crystallogr. Sect. C*, 2015, **71**(*1*), 3–8; (c) P. Müller, *Crystallogr. Rev.*, 2009, **15**(*1*), 57–83.
7. F. Neese, *Wiley Interdiscip. Rev.: Comput. Mol. Sci.*, 2025, **15**(*2*).
8. (a) S. Grimme, A. Hansen, S. Ehlert and J.-M. Mewes, *J. Chem. Phys.*, 2021, **154**(*6*); (b) F. Neese, *J. Comput. Chem.*, 2003, **24**(*14*), 1740–1747; (c) D. Bykov, T. Petrenko, R. Izsák, S. Kossmann, U. Becker, E. Valeev and F. Neese, *Mol. Phys.*, 2015, **113**(*13-14*), 1961–1977; (d) E. Caldeweyher, C. Bannwarth and S. Grimme, *J. Chem. Phys.*, 2017, **147**(*3*); (e) S. Lehtola, C. Steigemann, M. J. T. Oliveira and M. A. L. Marques, *SoftwareX*, 2018, **7**, 1–5; (f) E. Caldeweyher, S. Ehlert, A. Hansen, H. Neugebauer, S. Spicher, C. Bannwarth and S. Grimme, *J. Chem. Phys.*, 2019, **150**(*15*); (g) E. Caldeweyher, J.-M. Mewes, S. Ehlert and S. Grimme, *Phys. Chem. Chem. Phys.*, 2020, **22**(*16*), 8499–8512; (h) F. Neese, *J. Comput. Chem.*, 2022, **44**(*3*), 381–396; (i) L. Wittmann, I. Gordiy, M. Friede, B. Helmich-Paris, S. Grimme, A. Hansen and M. Bursch, *Phys. Chem. Chem. Phys.*, 2024, **26**(*32*), 21379–21394.
9. (a) F. Neese, F. Wennmohs, A. Hansen and U. Becker, *Chem. Phys.*, 2009, **356**(*1-3*), 98–109; (b) S. Grimme, J. Antony, S. Ehrlich and H. Krieg, *J. Chem. Phys.*, 2010, **132**(*15*); (c) S. Grimme, S. Ehrlich and L. Goerigk, *J. Comput. Chem.*, 2011, **32**(*7*), 1456–1465; (d) B. Helmich-Paris, B. de Souza, F. Neese and R. Izsák, *J. Chem. Phys.*, 2021, **155**(*10*).

10. T. Lu and F. Chen, *J. Comput. Chem.*, 2012, **33**(*5*), 580–592.
11. E. D. Glendening, C. R. Landis and F. Weinhold, *J. Comput. Chem.*, 2019, **40**(*25*), 2234–2241.
12. J. A. Smith and K. D. Moeller, *Org. Lett.*, 2013, **15**(*22*), 5818–5821.
13. T. Matsuo, K. Suzuki, T. Fukawa, B. Li, M. Ito, Y. Shoji, T. Otani, L. Li, M. Kobayashi, M. Hachiya, Y. Tahara, D. Hashizume, T. Fukunaga, A. Fukazawa, Y. Li, H. Tsuji and K. Tamao, *Bull. Chem. Soc. Jpn.*, 2011, **84**(*11*), 1178–1191.
14. L. Wagner, *Org. Synth.*, 2025, **102**, 350–366.
15. L. J. Irwin, J. H. Reibenspies and S. A. Miller, *J. Am. Chem. Soc.*, 2004, **126**(*51*), 16716–16717.
